# Microbial signatures and continuum in endometrial cancer and benign patients

**DOI:** 10.1186/s40168-024-01821-0

**Published:** 2024-07-01

**Authors:** Anita Semertzidou, Eilbhe Whelan, Ann Smith, Sherrianne Ng, Lauren Roberts, Jan J. Brosens, Julian R. Marchesi, Phillip R. Bennett, David A. MacIntyre, Maria Kyrgiou

**Affiliations:** 1 Institute of Reproductive and Developmental Biology, Department of Digestion, Metabolism and Reproduction, Department of Surgery and Cancer, Imperial College Faculty of Medicine, Room 3006, 3rd Floor, Du Cane Road, London, W12 0NN UK; 2 Department of Obstetrics & Gynaecology, Imperial College Healthcare NHS Trust, London, W12 0HS UK; 3 Faculty of Health and Applied Sciences, University West of England, Glenside Campus, Bristol, BS16 1DD UK; 4 Division of Reproductive Health, Warwick Medical School, Clinical Sciences Research Laboratories, University Hospital, Coventry, CV2 2DX UK; 5 Division of Digestive Diseases, Department of Digestion, Metabolism and Reproduction, Imperial College London, London, W2 1NY UK

**Keywords:** Microbiome, Female genital tract, Endometrial cancer

## Abstract

**Background:**

Endometrial cancer is a multifactorial disease with inflammatory, metabolic and potentially microbial cues involved in disease pathogenesis. The endometrial cancer microbiome has been poorly characterised so far and studies have often overestimated bacterial biomass due to lack of integration of appropriate contamination controls. There is also a scarcity of evidence on the functionality of microbial microenvironments in endometrial cancer. This work addresses that knowledge gap by interrogating the genuine, contamination-free microbial signatures in the female genital tract and rectum of women with endometrial cancer and the mechanistic role of microbiome on carcinogenic processes.

**Results:**

Here we sampled different regions of the reproductive tract (vagina, cervix, endometrium, fallopian tubes and ovaries) and rectum of 61 patients (37 endometrial cancer; 24 benign controls). We performed 16S rRNA gene sequencing of the V1–V2 hypervariable regions and qPCR of the 16S rRNA gene to qualitatively and quantitatively assess microbial communities and used 3D benign and endometrial cancer organoids to evaluate the effect of microbial products of *L. crispatus*, which was found depleted in endometrial cancer patients following primary analysis, on endometrial cell proliferation and inflammation. We found that the upper genital tract of a subset of women with and without endometrial cancer harbour microbiota quantitatively and compositionally distinguishable from background contaminants. Endometrial cancer was associated with reduced cervicovaginal and rectal bacterial load together with depletion of *Lactobacillus* species relative abundance, including *L. crispatus,* increased bacterial diversity and enrichment of *Porphyromonas*, *Prevotella, Peptoniphilus* and *Anaerococcus* in the lower genital tract and endometrium. Treatment of benign and malignant endometrial organoids with *L. crispatus* conditioned media exerted an anti-proliferative effect at high concentrations but had minimal impact on cytokine and chemokine profiles.

**Conclusions:**

Our findings provide evidence that the upper female reproductive tract of some women contains detectable levels of bacteria, the composition of which is associated with endometrial cancer. Whether this is a cause or consequence of cancer pathophysiology and what is the functional significance of this finding remain to be elucidated to guide future screening tools and microbiome-based therapeutics﻿.

Video Abstract

**Supplementary Information:**

The online version contains supplementary material available at 10.1186/s40168-024-01821-0.

## Background

The incidence of endometrial cancer has increased by 55% since the early 1990s [1, 2]. This is partly attributable to the increased prevalence of obesity and diabetes [3–7]. Only a fraction (2–5%) of cases can be ascribed to inherited genetic predisposition [8], while epidemiological studies have also shown strong correlations with pelvic inflammatory disease (PID) caused by bacteria [9], suggesting a potential microbial link to endometrial carcinogenesis. Dysbiotic microenvironments have recently been associated with gynaecological precancer and cancer [10–15] and their role in endometrial oncogenesis warrants further investigation.

The sterile womb dogma has been recently challenged with the advent of sensitive molecular tools, including bacterial 16S rRNA gene profiling, which enables the identification of microbes that were considered uncultivable [16]. Although recent studies report the detection of metataxonomic signatures in 60–97% of non-malignant uteri [17–24], experimental design pitfalls have often failed to account for potential sources of contamination, including niche-to-niche contamination caused by transcervical sample collection or non-patient sources from airborne and “kitome” contaminants [25–27]. There is currently no consensus on a benign endometrial bacterial signature although commonly reported genera include *Lactobacillus*,* Prevotella*, *Gardnerella*, *Bifidobacterium*,* Staphylococcus*, and *Streptococcus *[18–24, 28–31]. Intra-individual microbial correlations between the lower and upper genital tract, consistent with anatomical contingency, have recently been reported by Chen and colleagues [28]. Of the few studies to explore the endometrial oncobiome, an enrichment of seventeen taxa in the genital tract of endometrial cancer patients has been described, with *P. somerae* having the strongest association with disease [10, 11].

In this study, we aimed to characterise the bacterial load and composition of the female genital tract (FGT) in women with and without endometrial cancer (EC). Where detectable above background contamination, we aimed to assess whether the composition of the upper genital tract (endometrium, fallopian tubes, ovaries) was correlated with the lower genital tract (vagina, cervix) composition. We next set out to compare the FGT and rectal microbiota in endometrial cancer patients in relation to benign controls. Finally, we attempted to investigate the impact of *L. crispatus*, the species most significantly depleted in women with endometrial cancer following primary analysis, on endometrial organoid proliferation and inflammation. Delineating the microbiota in endometrial cancer patients and potential divergence from non-malignant controls could shed light on pathogenetic mechanisms but also guide the development of screening tools for early detection and management of the disease.

## Methods

### Study population and sample collection

To assess the female genital tract and rectal microbiome of women with endometrial cancer in relation to the non-malignant state, we recruited women planned for laparoscopic or transabdominal hysterectomy for endometrial cancer or benign conditions (most commonly dysfunctional uterine bleeding and/or fibroids). Women were recruited irrespective of the surgical approach (transabdominal open or laparoscopic) or endometrial cancer histological type. We excluded women undergoing hysterectomy for other gynaecological malignancies or pelvic inflammatory disease. Women who undertook vaginal douching or had taken antibiotics within the previous two weeks and women who had had sexual intercourse in the 48 h prior to sampling were also excluded. All participants gave informed consent.

**Fig. 1:**
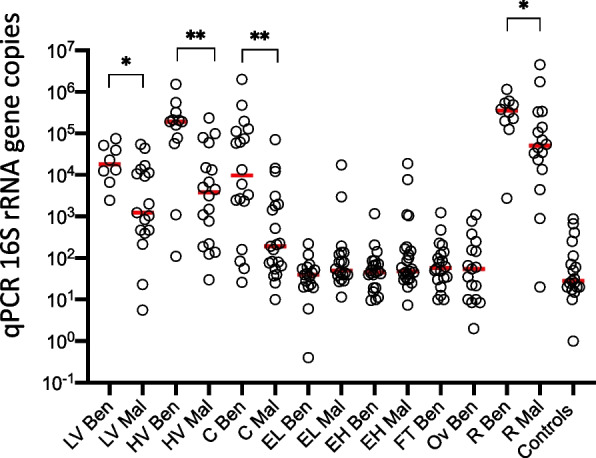
qPCR bacterial load at different locations in benign, endometrial cancer patients and controls. Red line represents the median. Ben: benign; Mal: malignant; LV: lower vagina; HV: higher vagina; C: cervix; EL: endometrium lower; EH: endometrium higher; FT: fallopian tube; Ov: ovary; R: Rectum. *p value < 0.05, **p value < 0.005

Microbiome swabs (VWR Swab Liquid Plastic Amies) were collected from seven distinct regions of the FGT and the rectum by rotating the swab five times against the sites of interest (Fig. 1A). FGT locations included the lower GT, i.e. lower two-thirds of the vagina, higher one-third of the vagina and cervical os and the higher GT, i.e. lower half of endometrium, fundal endometrium, fallopian tubes and ovaries only for benign samples. Vaginal, cervical and rectal swabs were collected pre-operatively in the operating room, using an unlubricated, disposable, sterile plastic speculum (Medscope Intraspec) before antibiotic administration. In addition to this, a sterile cotton vaginal swab was collected and stored at − − 80^°^C in 8% v/v DMSO for bacterial cryopreservation. The surgical specimen was placed in a sterile sealed bag inside a histology pot and stored at 4 °C before being transferred to the histopathology lab on the same day 1–6 hh post-extraction. Under aseptic conditions, the fimbrial end of the fallopian tube and the external surface of the ovary were swabbed, while endometrial samples were collected after longitudinal dissection of the uterus. Tissue samples from the cervix, endometrium and fallopian tube were also collected. All samples were stored at − − 80 °C^°^C within 30 min. For most cases, matched samples from the same patient along the genital tract were obtained. Two sets of technical controls were used in the pathology lab and were collected on a series of different days. A lysogeny broth (LB) agar plate was left open during sample collection and swabbed to account for airborne contaminants, while the packaged, non-sterile knife used for uterus dissection was also sampled. All specimens were processed in the same pathology lab.

### DNA2 extraction and 16S rRNA gene sequencing

Bacterial DNA from swabs and tissue was extracted using the QiAmp Mini DNA kit. The V1–V2 hypervariable regions of 16S rRNA genes were amplified for sequencing using forward and reverse fusion primers. The forward primer consisted of an Illumina i5 adapter (5′-AATGATACGGCGACCACCGAGATCTACAC-3′), an 8-base-pair (bp) bar code, a primer pad (forward, 5′-TATGGTAATT-3′) and the 28F primer (5′-GAGTTTGATCNTGGCTCAG-3′). The reverse fusion primer was constructed with an Illumina i7 adapter (5′-CAAGCAGAAGACGGCATACGAGAT-3′), an 8-bp bar code, a primer pad (reverse, 5′-AGTCAGTCAG-3′) and the 388R primer (5′-TGCTGCCTCCCGTAGGAGT-3′). Sequencing was performed in the Digestion, Metabolism and Reproduction Department (St Mary’s Hospital, London, UK) using an Illumina MiSeq platform (Illumina Inc.).

### 16S rRNA gene sequence analysis

Sequence data was processed in Mothur using the MiSeq SOP Pipeline [32]. OTUs were defined using a cut-off value of 97% and the result data was analysed using Vegan package within the R statistical package for assessment of microbial composition and diversity (R Development Core Team 2008). OTUs were subsequently randomly sub-sampled to the lowest common read count for each site to avoid sequencing bias. This technique retained 96% of OTU counts and still provided coverage of greater than 95% for all samples**.** OTU taxonomies (from Phylum to Genus) were determined using the ribosomal database project (RDP) MultiClassifier script to generate the RDP taxonomy, whereas species-level taxonomies of the OTUs were determined using the USEARCH algorithm (v.11) [33, 34] combined with the cultured representatives from the RDP and STIRRUPS databases. Alpha and beta indices were calculated from these datasets within Mothur and the Vegan package within the R environment.

### Removal of contaminating sequence reads

As low bacterial biomass samples are prone to environmental and kit contamination, we collected four different sets of negative controls to account for air, equipment (knife) and kit (DNA extraction and 16S rRNA gene sequencing) sources of contamination. Taxonomic units were identified as likely contaminants using the Decontam R package [35] (v1.6.0) with a prevalence-based threshold of 0.5 where a *p*-value calculated using chi-square/Fisher’s test below 0.5 was identified as a contaminant.

### Determining genuine signatures and microbiota continuum

Following the application of the Decontam R package to exclude likely contaminant OTUs, we additionally filtered our data by including only OTUs with at least 5 counts in 10% of samples and a low variance (IQR) of 5%. The remaining OTUs in both patient samples and controls were compared through hierarchical clustering analysis (HCA) at the genera level to determine taxa enriched in low biomass samples versus contaminant controls.

We defined as a continuum the presence of bacterial species at a relative abundance of at least 0.5% in matched samples collected from the lower and upper genital tract. Only patients with matched samples along the reproductive tract were selected for this analysis. In benign patients, this included the vagina, cervix, endometrium, fallopian tube and ovary. In EC patients, samples included the vagina, cervix and endometrium. For the EC cohort, samples of fallopian tubes and ovaries were not analysed as we speculated that the low-biomass microbiome in these locations would most likely not impact on carcinogenic events in the endometrium. In both patient cohorts, the vagina and rectum were sampled and matched samples were analysed. Venn diagrams were used to depict patterns of overlapping cooccurrence of taxa among sites. For this, high vaginal and fundal endometrial samples were used.

We used the Sorensen index (Sørensen–Dice index) to measure the similarity between microbiota at different anatomic sites of the same individual. The index was calculated as

$$DSC=\left(\frac{2\cdot\mathrm c}{S_1+S_2}\right)$$

where *S1* and *S2* are the number of OTUs in community 1 and community 2 respectively, and *c *is the number of OTUs shared by the two communities; *DSC* is the Sorensen coefficient, a similarity index indicator, which ranges from 0 to 1, with 1 signifying the greatest similarity.

### Compositional comparison of microbiota in endometrial cancer versus benign controls

Analysis of statistical differences between microbiota of women with and without endometrial malignancy was performed using the Statistical Analysis of Metagenomic Profiles (STAMP) package (v.2.1.3) [36], ClustVis [37] and Marker-gene Data Profiling (MDP) module of MicrobiomeAnalyst [38]. Data were subjected to multivariate analysis using hierarchical clustering analysis by Ward clustering and Euclidean distance with a density threshold of 0.75 and were rarefied to minimum library size prior to analysis. The most commonly identified genera were included, while the remaining OTUs were classified as others. Differences in mean α-diversity (Shannon index), which reflects richness and evenness within bacterial populations of each sample, were assessed using the Mann–Whitney/Kruskal–Wallis statistical test as appropriate, while β-diversity was calculated using the Bray–Curtis index and compared using PERMANOVA.

Linear discriminant analysis (LDA) effect size (LEfSe) analysis [39] was used to identify taxa significantly overrepresented in endometrial cancer patients when compared to benign controls at multiple taxonomic levels. This analysis was performed using taxonomic relative abundance, with per-sample normalisation and default settings for alpha values (0.05) for the factorial Kruskal–Wallis test among classes and pairwise Wilcoxon test between subclasses. A logarithmic LDA score >  2 was used to determine discriminative features.

### Quantitative real-time PCR (qPCR) of the 16S rRNA gene

Quantitative real-time PCR was carried out for quantification of the 16S rRNA gene copy number to determine the bacterial load at various anatomical sites of benign and endometrial cancer patients. Real-time qPCR was performed with universal BactQUANT 16S rRNA gene primers (Forward primer: 5′-CCTACGGGAGGCAGCA, Reverse primer: 5′-GGACTACCGGGTATCTAATC) (Sigma) with the FAM labeled BactQUANT probe ((6FAM) 5′-CAGCAGCCGCGGTA-3′ (MGBNFQ)) on the Applied Biosciences StepOne machine (Thermo Fisher Scientific, Ashford, UK) with StepOne software version 2.3 (Life Technologies). Each 20 μl reaction included lyophilised genomic DNA from *Escherichia coli (E. coli*) Strain B (Sigma, Dorset, UK) harbouring 7 copies of 16S rRNA gene, which was serially diluted in diethylpyrocarbonate (DEPC) water to make a ten-fold standard curve and Platinum Supermix UDG (including ROX) (ThermoFisher Scientific). Sterile water was used as a negative control. Cycling conditions were 50 ^o^C for 2 min, 95 ^o^C for 10 s, 95^o^C for 15 s, 60 ^o^C for 60 s, 95^o^C for 15 s for 40 more times. Total DNA amount for each sample was calculated by multiplying 16S rRNA gene quantity in each loaded sample (5 μL) by the total volume of extracted DNA (50 μL).

### Lactobacillus crispatus culture

*L. crispatus* isolated from the high vaginal swab of a benign patient and stored in 8% v/v DMSO at -80 °C or commercially sourced (*L. crispatus* strain ATCC 33820, DSMZ, Germany) was cultured anaerobically in MRS broth (Sigma-Aldrich) overnight at 37 °C to a density of ~  10^8^ CFU/ml. The culture supernatant was collected after 24 h following centrifugation and neutralised with a 1 M NaOH solution. *L. crispatus* isolated from the patient swab was identified using near full-length 16S rRNA gene sequencing and the USEARCH and BLASTn software.

### Human endometrial glandular organoid cultures in L. crispatus-conditioned media

Pipelle endometrial biopsies were used to generate endometrial glandular organoids following the Cambridge protocol [40]. Briefly, endometrial glands were separated from stromal cells and following digestion with collagenase V/DNAse I, the pellet was re-suspended in Matrigel and 20 μl droplets were deposited in pre-warmed 48-well plates. Organoids were grown in Advanced DMEM, supplemented with EGF, FGF10, HGF, Rspondin 1, Nicotinamide and Noggin at 37 °C and 5% CO_2_. The medium was changed every 2 days and organoids passed at a 1:2 ratio every 2–3 weeks. The purity of EC organoid cultures was tested through genetic and epigenetic comparison with parent tumours (data not shown). To explore the effect of *L. crispatus*-secreted biomolecules on endometrial organoid proliferation and inflammation, benign and malignant endometrial organoids were cultured in media conditioned with *L. crispatus* supernatant at increasing concentrations (10%, 20%, 30% v/v), with (benign organoids only) or without 1 μg/ml LPS (*E. coli* O111:B4). Controls were grown in plain MRS broth-supplemented medium containing 1 MM NaOH using 10%, 20% and 30% v/v concentrations.

### Organoid viability assay

To assess the effect of *L. crispatus* conditioned media on endometrial organoid proliferation, we performed the CellTiter-GLo® 3D cell viability assay, which quantifies intracellular ATP, according to manufacturer’s protocol (Promega). Briefly, the medium was removed following treatments and endometrial cells were lysed in 100 μl pre-warmed CellTiter-GLo 3D reagent. Samples were incubated for 30 min at room temperature and luminescence was recorded using a plate reader (PheraStar).

### Confocal microscopy

To confirm that the LPS receptor, TLR4, is expressed in endometrial organoids, we fixed them in 10% v/v formaldehyde, permeabilised in blocking buffer plus 0.3% Triton-X and incubated with the anti-TLR4 primary antibody (Thermo Fisher Scientific) at 4 °C overnight, followed by incubation with the fluorescent conjugated anti-rabbit Alexa Fluor secondary antibody (Thermo Fisher Scientific) for 2 h at room temperature. Both primary and secondary antibodies were diluted (1:100 primary, 1:200 secondary) in the blocking buffer. Negative controls were prepared by omitting the primary antibody. Organoids were imaged using the inverted Leica SP5 confocal system and software.

### Magnetic Luminex cytokine assay

To investigate if *L. crispatus*-conditioned medium influences cytokine and chemokine secretion by endometrial organoids, benign and malignant endometrial glandular organoids were stimulated with LPS (*E. coli* O111:B4) for 24 h or vehicle in the presence of *L. crispatus*-conditioned media at increasing concentrations (10%, 20%, 30% v/v). A total of 50 µl of undiluted supernatants were then collected and the concentrations of up to 12 different cytokines and chemokines were analysed using a Magnetic Multiplex Cytokine Array (R&D systems) following the manufacturer’s instructions with a limit of detection of 1–10 pgpg/ml. For endometrial cancer organoid supernatants, the following analytes were measured: CCL4/MIP1beta, CCL5/Rantes, G-CSF, GM-CSF, IL-1rα, IL-1β/IL-1F2, IL-2, IL-6, IL-8/CXCL8, IFNγ, TNFα and VEGF. For benign organoid culture supernatants, the concentrations of IL-1β/IL-1F2, IL-6, IL-8/CXCL8, IL-10, IFNγ and TNFα were measured. All samples were assayed in duplicate. Total protein concentration of supernatant in each well was determined using a Bradford protein assay (Quick Start™ Bradford Protein Assay kit 2, Bio-rad). To correct for different cell numbers making up the organoids in each well, a correction factor was calculated by dividing the total protein concentration of each well (μg/mL) by the total protein concentration of one of the samples (separately for benign and malignant organoids), which was then multiplied by the cytokine/chemokine concentration (pg/mL) in each well.

### Statistical analyses

Other statistical analyses described in this study were performed using the statistical package GraphPad Prism v.8.0.1 (GraphPad Software Inc., CA, USA). The normality of data was assessed by the Shapiro–Wilk test. Sorensen indices, viability and cytokine results were analysed by *t*-test, ANOVA Friedman test and two-tailed Mann–Whitney test. Dunn’s multiple comparisons test was used to adjust for multiple cytokine comparisons. A *p* value less than 0.05 was considered statistically significant.

## Results

### Patient demographics and characteristics

Sixty-one women undergoing hysterectomy were recruited; 37 had endometrial cancer (61%) and 24 were benign controls (39%). In total, 178 benign, 207 malignant and 51 technical control samples were sequenced and analysed. Patient and clinical characteristics are shown in Table 1. Key clinical characteristics of the two groups were largely comparable apart from age (endometrial cancer ≥  65, 56.8%; benign 50–64, 58.3%,* p* =  0.0077). Post-menopausal women were equally distributed in both cohorts and none of the patients reported current use of exogenous hormones. Most endometrial cancers were endometrioid tumours (30/37, 81%) and stage I (31/37, 83.8%).

**Table 1:** Patient demographic and clinical characteristics

**Patient characteristics**	**Benign** (***n*** = **24)**	**Endometrial ca** (***n*** = **37)**	**Total** (***n*** = **61)**	**OR (95% CI)/*P*-value**	*P* value* (benign vs cancer**)**
**Ethnicity, *n*/*N* (%)**					0.1156
White	17/24 (70.8)	20/37 (54.1)	37/61 (60.7)		
Asian	1/24 (4.2)	10/37 (27)	11/61 (18)	0.12 (0.01, 1.01)/0.035	
Black/African/Caribbean	5/24 (20.8)	5/37 (13.5)	10/61 (16.4)	1.18 (0.29, 4.76)/1.00	
Other	1/24 (4.2)	2/37 (5.4)	3/61 (4.9)	0.59 (0.05, 7.07)/1.00	
**Age (years), *n*/*N* (%)**					0.0077
18–**34**	1/24 (4.2)	0/37 (0)	1/61 (1.7)		
35–**49**	4/24 (16.7)	1/37 (2.7)	5/61 (8.2)	1.00 (0.02, 40.28)/1.00	
50–**64**	14/24 (58.3)	15/37 (40.5)	29/61 (47.5)	0.31 (0.01, 8.28)/0.484	
≥ **65**	5/24 (20.8)	21/37 (56.8)	26/61 (42.6)	0.09 (0.003, 2.39)/0.064	
**BMI status, *n*/*N* (%)**					0.8817
Underweight (< 18.5)	1/24 (4.2)	2/37 (5.4)	3/61 (4.9)	0.50 (0.04, 7.10)/1.00	
Normal (18.5–**24.9)**	6/24 (25)	6/37 (16.2)	12/61 (19.7)		
Overweight (25–**29.9)**	7/24 (29.1)	13/37 (35.2)	20/61 (32.8)	0.59 (0.05, 7.07)/0.473	
Obese (≥ **30)**	10/24 (41.7)	16/37 (43.2)	26/61 (42.6)	0.54 (0.13, 2.31)/1.00	
**Parity, *n*/*N* (%)**					0.583
Nulliparous	5/24 (20.8)	10/37 (27)	15/61 (24.6)		
Parous	19/24 (79.2)	27/37 (73)	46/61 (75.4)	1.41 (0.41, 4.78)	
**Menopausal status**^a^**, *n*/*N* (%)**					0.373
Premenopausal	3/24 (12.5)	2/37 (5.4)	5/61 (8.2)		
Postmenopausal	21/24 (87.5)	35/37 (94.6)	56/61 (91.8)	0.40 (0.06, 2.59)	
**Contraceptive pill use, *n*/*N* (%)**					0.735
Ever users	16/24 (66.7)	16/37 (43.2)	32/61 (52.5)	2.63 (0.90, 7.65)	
Never users	8/24 (33.3)	21/37 (56.8)	29/61 (47.5)		
**HRT use, *n*/*N* (%)**					0.065
Ever users	8/24 (33.3)	5/37 (13.5)	13/61 (21.3)	3.20 (0.90, 11.38)	
Never users	16/24 (66.7)	32/37 (86.5)	48/61 (78.7)		
**Diabetes status, *n*/*N* (%)**					0.751
Diabetic	5/24 (20.8)	9/37 (24.3)	14/61 (23)	0.82 (0.24, 2.83)	
Nondiabetic	19/24 (79.2)	28/37 (75.7)	47/61 (77)		
**Smoking, *n*/*N* (%**					0.291
Ever smokers	11/24 (45.8)	12/37 (32.4)	23/61 (37.7)	1.76 (0.61, 5.08)	
Never smokers	13/24 (54.2)	25/37 (67.6)	38/61 (62.3)		
**Current coffee consumption, *n*/*N* (%)**					0.297
Yes	13/24 (54.2)	15/37 (40.5)	28/61 (45.9)	1.73 (0.61, 4.89)	
No	11/24 (45.8)	22/37 (59.5)	33/61 (54.1)		
**Antibiotic use last 4** **weeks, *n*/*N* (%)**					0.298
Yes	5/24 (20.8)	4/37 (10.8)	9/61 (14.8)	2.17 (0.52, 9.08)	
No	19/24 (79.2)	33/37 (89.2)	52/61 (85.2)		
**Surgical approach**					
Laparoscopic	15/24 (62.5)	34/37 (91.9)	49/61 (80.3)		
Transabdominal	9/24 (37.5)	3/37 (8.1)	11/61 (18)		
**Histological type, *n*/*N* (%)**					NA
Endometrioid	NA	30/37 (81)			
Serous	NA	3/37 (8)			
Clear cell	NA	3/37 (8)			
Carcinosarcoma	NA	1/37 (3)			
**FIGO Grade, *n*/*N* (%)**					NA
I	NA	12/37 (32.4)			
II	NA	11/37 (29.7)			
III	NA	14/37 (37.8)			
**FIGO Stage 2009, *n*/*N* (%)**					NA
IA	NA	26/37 (70.3)			
IB	NA	5/37 (13.5)			
II	NA	3/37 (8.1)			
IIIA	NA	1/37 (2.7)			
IIIC	NA	1/37 (2.7)			
IV	NA	1/37 (2.7)			

^*^Statistical significance (*p* value) assessed by Fisher’s exact test

^a^Menopause was defined as permanent cessation of menses for at least 12 months

### Bacterial signatures above background contamination in low bacterial biomass sites

The presence of a microbial metataxonomic signature above background contamination in the endometrium, fallopian tubes and ovaries of benign patients and endometrium of endometrial cancer patients was performed in careful consideration of potential sources of contamination (Supplementary Table S1). Sequencing read data was obtained from all samples; however, 5 vaginal, 10 cervical and 25 endometrial samples were excluded from analysis due to low read counts (Supplementary Table S3). Exploration of negative control samples identified a total of 1037/6818 (15%) OTUs in the dataset that were likely contaminants (Supplementary Table S2). These were removed from further analyses. As expected, the effect of environmental and kit contaminant removal was more pronounced in low-biomass sites (Supplementary Figure S1).

**Fig. 2:**
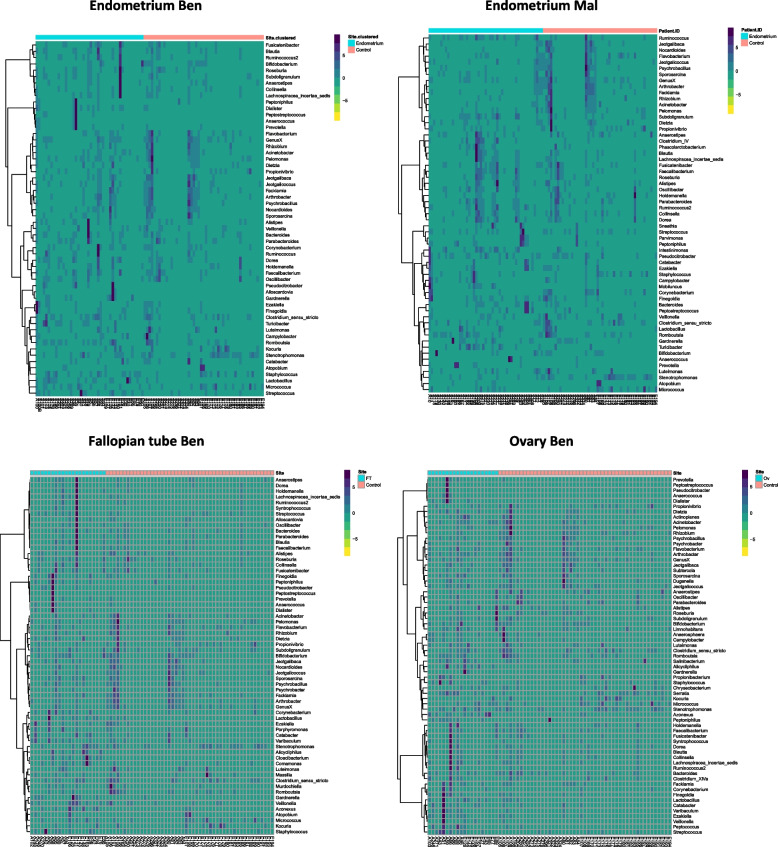
Prominent operational taxonomic units (OTUs) among low biomass patient samples and technical controls. Compositional comparison of microbial signatures between low-biomass sites (blue) of benign/endometrial cancer patients and technical controls (pink) demonstrates high overlap of malignant endometrium with controls, whereas low-biomass sites in benign patients exhibit distinct taxa that are not recovered from controls. Ben: benign; Mal: malignant; FT: fallopian tube; Ov: ovary

We next estimated bacterial copy numbers at different locations within the FGT and rectum in both benign and endometrial cancer samples. Low biomasses were observed in the endometrium, fallopian tubes and ovaries that were comparable to negative controls. These values were one to four orders of magnitude lower than the vagina, cervix and rectum samples (Fig. 1). Applying a cut-off above control counts at 700 bacterial copies based on sequencing reads, a microbial signature above background contamination was observed in 62% of benign endometrium, 50% of malignant endometrium, 85% of benign fallopian tube and 95% of benign ovary. We next compared the bacterial composition at the genus level between the low-biomass samples of the upper GT and controls. The compositional signatures of benign endometrium, fallopian tube and ovary exhibited taxa that were discernible from technical controls, whilst malignant endometrial samples displayed higher overlap with controls probably reflecting a less prominent microbial signature rather than increased contamination (Fig. 2).

Of note, a comparison of 10 paired swab-tissue samples (cervix, endometrium and fallopian tube) from benign and EC patients revealed compositional dissimilarity in most samples based on 16S rRNA gene amplicons (Supplementary Figure S2A). α- and β-diversity was not significantly different between tissue and swab samples (Supplementary Figure S2B).

Most recruited patients underwent laparoscopic hysterectomy (49/61, 80.3%). We assessed whether practices adopted in laparoscopic hysterectomy (15/24, 62.5% of benign cases and 34/37, 91.9% of malignant cases) introduce intra-patient contamination in low bacterial biomass sites. To do this, we compared the microbial composition of fallopian tubes and ovaries against higher vaginal samples intra-individually to test for contamination during vaginal retrieval of specimens and lower/higher endometrium against cervical samples to identify contamination during uterine manipulation. Transabdominal (*n* =  13) and laparoscopic (*n* =  13) hysterectomies performed for either benign indications or endometrial malignancy were compared at the species level to increase metataxonomic resolution (Supplementary Figure S3). In samples collected during transabdominal surgery, the microbiota of fallopian tubes and ovaries were either highly positively or negatively correlated with vaginal composition. In laparoscopic procedures, a spread of correlation was observable in 2/8 cases (25%). With regards to contamination of the lower endometrium by the adjacent cervix during instrumentation, correlation follows the same pattern in both surgical techniques. When comparing higher endometrium to matched cervical samples, a strong overlap of taxa was observed in around half of the patients in the transabdominal approach. In laparoscopy, a spread of correlation data is noted, suggestive of potential microbial transfer from the cervix into the fundal endometrium in one-third (4/12, 33%) of patients.

### Investigation of a microbial continuum in benign and endometrial cancer patients

**Fig. 3:**
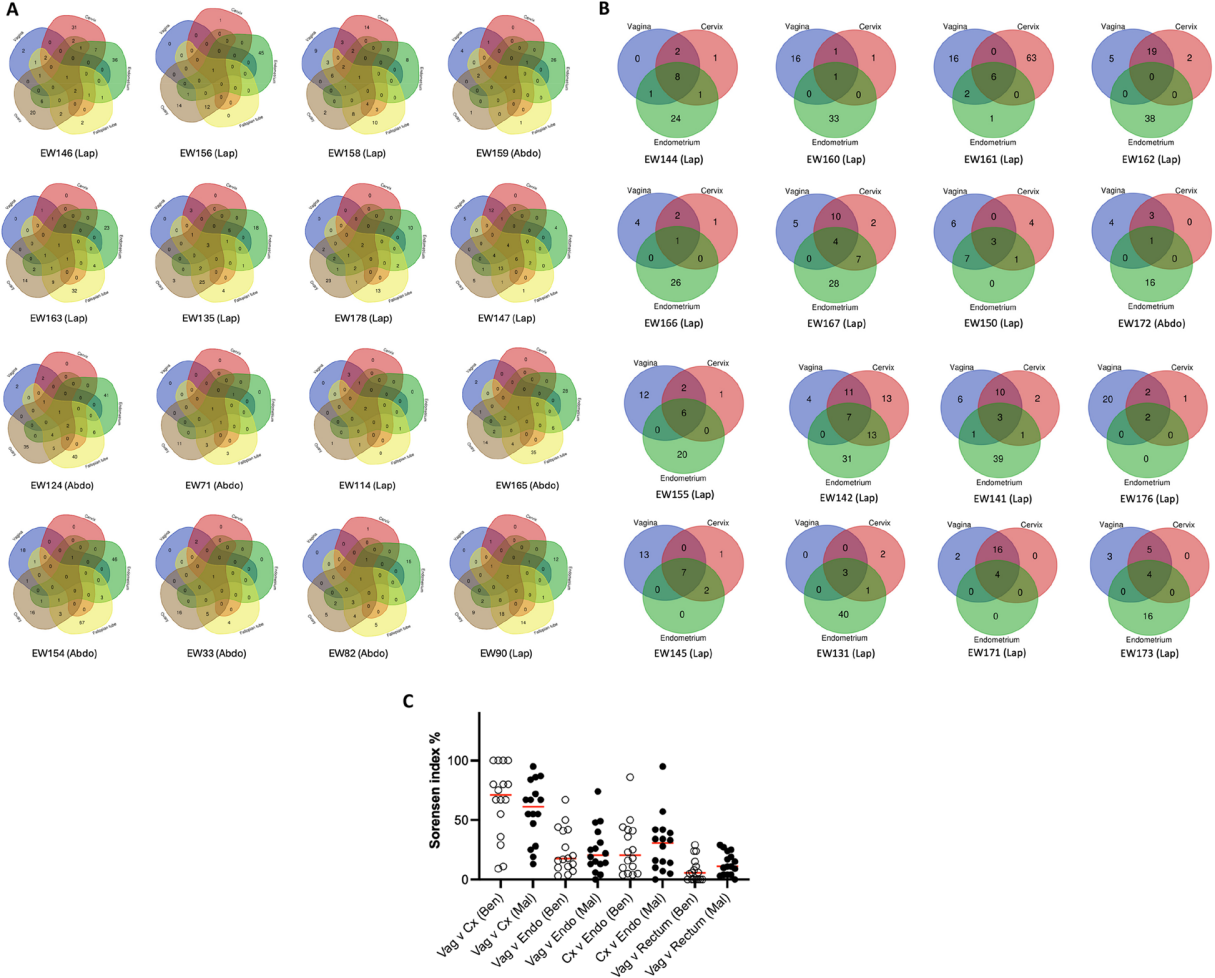
Microbial continuum along the female genital tract. **A** Venn diagram illustrating microbial species shared by all sites of the lower and upper genital tract (vagina, cervix, endometrium, fallopian tube, ovary) in 16 patients with benign pathology. **B** Venn diagrams illustrating microbial species shared by the vagina, cervix, and endometrium in 16 patients with endometrial cancer. **C** Similarity analysis at species level between the lower and upper genital tract and between the vagina and rectum in benign and endometrial cancer patients. Circles and dots represent individual Sorensen indices % for each patient (*n* = 16 benign, *n* = 16 EC). Red lines denote the median. Only species with at least 0.5% relative abundance were included. *Lap: laparoscopic; Abdo: abdominal; Ben: benign; Mal: malignant; Vag: vagina; Cx: cervix; Endo: endometrium; v: versus*

We next compared the microbial composition along the vagina, cervix, endometrium, fallopian tubes and ovaries in 16 benign and the vagina, cervix and endometrium in 16 endometrial cancer patients, for whom matched samples had been collected (Fig. 3A, B). Similarity analysis intra-individually at the species level, calculated by the Sorensen index, in women with and without endometrial cancer showed a high correlation between the vagina and cervix and a lower correlation between the vagina and endometrium or the cervix and endometrium in both populations. The microbial continuum along the genital tract did not differ significantly between the benign and EC cohorts (Fig. 3C). In the benign cohort, lower genital tract microbiota were poorly correlated with upper microbiota with fallopian tubes and ovaries displaying higher correlation (median of Sorensen index =  45%) (Supplementary Figure S4A). Of note, however, in 75% (12/16) of benign patients, the most abundant species of the lower GT (vagina, cervix) were recoverable from all sites of the upper GT (endometrium, fallopian tubes, ovaries). To explore geographical variations within the same organ, sub-analysis within the vagina (lower 2/3 versus higher 1/3 of vagina) and endometrium (lower 2/3 versus fundal endometrium) at species level revealed high intra-patient correlation (*R*^2≥ ^ 0.7) in 92% of benign and 76% of endometrial cancer patients along the vagina and only 57% of benign and 31% of malignant samples along the endometrium at genera level (Supplementary Figure S4B, C). The lower microbial correlation within the malignant endometrium compared to benign was statistically significant (genus *p* =  0.0007; species *p* =  0.0003).

The concept of the rectal microbiota seeding the female genital tract has been previously suggested in the literature [41, 42]. The correlation of vaginal with rectal microbiota was sought in 16 women with benign pathology and 26 women with endometrial cancer, for whom matched samples were available. The vaginal ecosystem of benign patients shared no microbiome with the rectum in 44% (7/16) of cases, in 25% (4/16) of patients only one bacterial species was shared, whereas in 31% (5/16) of cases ≥  2 bacterial species overlapped with a median of 4 OTUs. In endometrial cancer patients, 15% (4/26) of cases displayed no shared microbiome between the vagina and rectum, 27% (7/26) shared one bacterial species, while 58% (15/26) had ≥  2 bacterial species in common with a median of 5 OTUs (Supplementary Figure S5). Inter-individual Sorensen indices % showed low concordance between vaginal and rectal samples in benign (median 5%) and EC patients (median 11%), which was not significantly different between the two populations (*p* =  0.258) (Fig. 3C). Taken together this evidence suggests that vaginal microbiota are poorly correlated with rectal microbiota in both benign and endometrial cancer patients.

### The genital tract and rectal microbiota in endometrial cancer patients and benign controls

We compared the microbial composition of 24 benign and 37 endometrial cancer patients at different anatomical sites (vagina, cervix, endometrium) and observed high diversity, *Lactobacillus* depletion and enrichment of *Porphyromonas*, *Prevotella, Peptoniphilus* and *Anaerococcus* in endometrial malignancy when compared to benign controls.

**Fig. 4:**
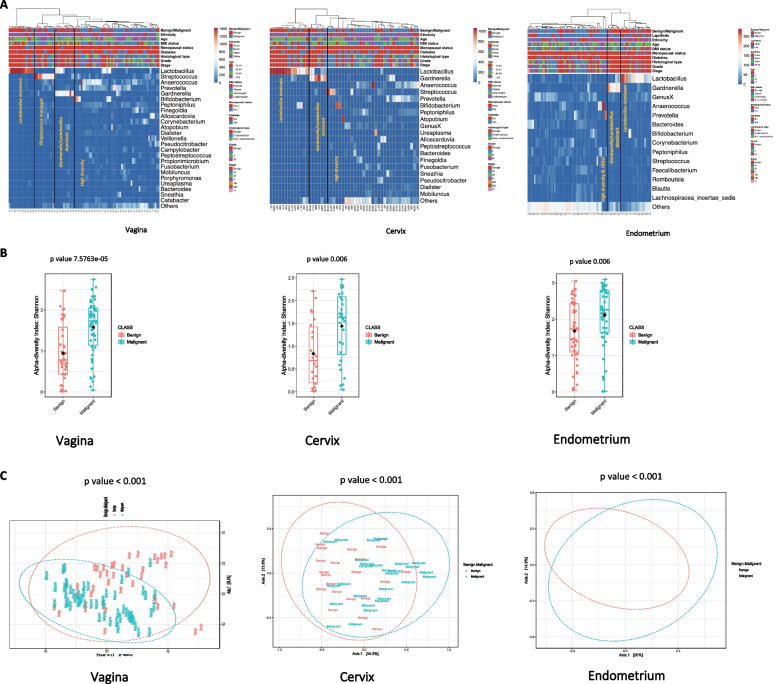
Vaginal, cervical and endometrial microbial composition at genera level according to the presence of endometrial malignancy. **A** Hierarchical clustering analysis per endometrial malignancy, ethnicity, age, BMI status, menopausal status, diabetes, histological type, grade and FIGO stage of disease using Ward linkage of the most commonly identified genera. Data were rarefied prior to analysis.** B** Microbiome Shannon α- and** C** β-diversity per anatomical site

Hierarchical clustering analysis (HCA) at the genera level revealed three distinct clusters of *Lactobacillus*-dominant (≥  75% relative abundance), *Gardnerella/Lactobacillus*-dominant (Gardnerella ≥  50% and Lactobacillus > > 40%) and High diversity/Other at all sites examined with an additional *Streptococcus* (≥  36%) cluster for vagina (Fig. 4A). High diversity/other cluster included samples with higher Shannon α-diversity than other clusters, but also a few samples dominated by one or two bacteria forming small clusters (Supplementary Figure S6). We examined the α- and β-diversity at genera level of the microbial community composition in patients with and without endometrial cancer (Fig. 4B, C). We found that patients with endometrial cancer display higher α-diversity than patients without endometrial cancer (vagina *p* =  7.5763e-05; cervix *p* =  0.006; endometrium* p* =  0.006). Similarly, β-diversity was significantly different between benign and endometrial cancer patients at all anatomical sites (vagina *p* <  0.001; cervix *p* <  0.001; endometrium *p* <  0.001).

The rates and frequency of different clusters were compared between patients with and without endometrial cancer (Table 2). *Lactobacillus*- and *Gardnerella/Lactobacillus-* dominant clusters were significantly higher among benign patients (vagina 30% and 22.5%, *p* =  0.000485; cervix 45.5% and 18.1%, *p* =  0.003821; Endometrium 41% and 20.6%, *p* <  0.00001 respectively), while high diversity/Other was the most prevalent cluster among endometrial cancer patients (vagina 72.2%, *p* =  0.000485; cervix 82.1%, *p* =  0.003821; endometrium 87.8%, *p* <  0.00001), which was also accompanied by *Lactobacillus* depletion.

**Table 2:** Genital tract microbial clusters in benign and endometrial cancer patients

Genus	*Lactobacillus* dominant *n*/*N* (%)	*Streptococcus* dominant *n*/*N* (%)	*Gardnerella/Lactobacillus* dominant *n*/*N* (%)	High diversity and other *n*/*N* (%)	*p* value
**Vagina**					
**Benign**	12/40 (30)	5/40 (12.5)	9/40 (22.5)	14/40 (35)	0.000485
**Endometrial cancer**	4/54 (7.4)	8/54 (14.8)	3/54 (5.6)	39/54 (72.2)	
**Total**	16/94 (17)	13/94 (13.8)	12/94 (12.8)		
**Cervix**					
**Benign**	10/22 (45.5)	-	4/22 (18.1)	8/22 (36.4)	0.003821
**Endometrial cancer**	3/28 (10.7)	-	2/28 (7.1)	23/28 (82.1)	
**Total**	13/50 (26)	-	6/50 (12)	31/50 (62)	
**Endometrium**					
**Benign**	18/44 (41)	-	9/44 (20.6)	17/44 (38.6)	< 0.00001
**Endometrial cancer**	5/49 (10.2)	-	1/49 (2)	43/49 (87.8)	
**Total**	23/93 (24.7)	-	10/93 (10.8)	60/93 (64.5)	

*p* value was calculated by chi-squared test

**Fig. 5:**
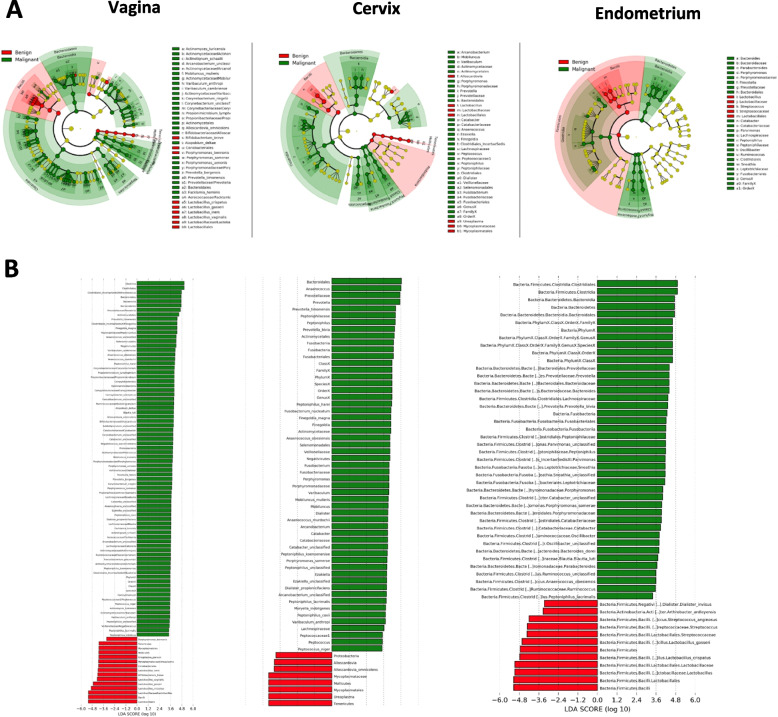
Significantly enriched taxa among patients with and without endometrial cancer in the upper and lower female genital tract. **A** Cladograms representing taxa with different abundances between the comparison groups per site. **B** Histograms of the LDA scores computed for features differentially abundant between endometrial cancer and benign patients. *LDA score: Linear discriminant analysis score*

Linear discriminant analysis (LDA) effect size (LEfSe) modelling was also used to identify differences in microbiota composition of endometrial cancer patients versus benign controls (Fig. 5A, B). In benign patients, we observed an over-representation of *Lactobacillus* species (*L. crispatus*, *L. gasseri*, *L. iners* and *L. vaginalis*) at all sites examined (vagina, cervix, endometrium) and *Bifidobacterium breve* in vagina and cervix. Conversely, in endometrial cancer patients, an enrichment of several microbes was observed at all sites, including *Porphyromonas*, *Prevotella*,* Peptoniphilus* and *Anaerococcus*.

We next sought to determine microbial differences based on histological type and grade of endometrial cancer. We included 30 endometrioid and 7 non-endometrioid endometrial tumours of different grades (grade 1: *n* =  12; grade 2: *n* =  11; grade 3: *n* =  14). According to histological type, Shannon a-diversity index was not significantly different between the endometrioid and non-endometrioid groups at genera level (vagina *p* =  0.084; cervix *p* =  0.365; endometrium *p* =  0.936), but β-diversity reached statistical significance in the vagina and endometrium (vagina *p* <  0.046; cervix *p* <  0.069; endometrium *p* <  0.001) (Supplementary Figure S7A). With regards to grade, α- and β-diversity were significantly different in the vagina (*p* =  0.006 and *p* <  0.007 respectively) but not in the cervix (*p* =  0.368 and *p* <  0.191 respectively) among different grades at the genera level. In the vagina, grade I lesions displayed the lowest α-diversity, whereas this was similar between grade II and III tumours. In the endometrium, no differences among grades were noted for Shannon α-diversity (*p* =  0.23) but β-diversity was significantly different (*p* <  0.001) (Supplementary Figure S7B). 

Given the high diversity observed in the rectum, we compared the rectal microbial makeup of patients with and without endometrial cancer at the phylum level. We noted that *Firmicutes* (*p* =  0.002), *Bacteroidetes* (*p* =  0.002), *Actinobacteria* (*p* =  0.002) and *Proteobacteria* (*p* =  0.007) were significantly depleted in endometrial cancer patients (Supplementary Figure S8A). On the other hand, rectal microbiota α- and β-diversity did not differ significantly between benign and endometrial cancer patients at the genera level (α-diversity: *p* =  0.455; β-diversity: *p* <  0.359) (Supplementary Figure S8B).

Finally, we inquired about microbial load differences in the genital tract and rectum of benign and endometrial cancer patients. Quantitative real-time PCR targeting the 16S rRNA gene revealed lower actual abundances by one-two orders of magnitude in the vagina, cervix and rectum of patients with endometrial malignancy versus benign controls, whilst no significant differences were noted in the endometrium, where bacterial quantities were comparable to the bacterial biomass of controls (Fig. 5B). Our findings indicate that endometrial cancer is marked by reduced bacterial colonisation of anatomical sites that normally display a high bacterial load.

### L. crispatus effect on endometrial organoid proliferation and inflammation

We tested the hypothesis that *L. crispatus*, which we found to dominate the vagina, cervix and endometrium of patients without endometrial cancer and was depleted in endometrial cancer patients, has an anti-inflammatory, anti-mitogenic effect on endometrial organoids. Organoids were derived from the endometrium of five benign and six endometrial cancer patients (Supplementary Table S4).

### Effect on proliferation

**Fig. 6:**
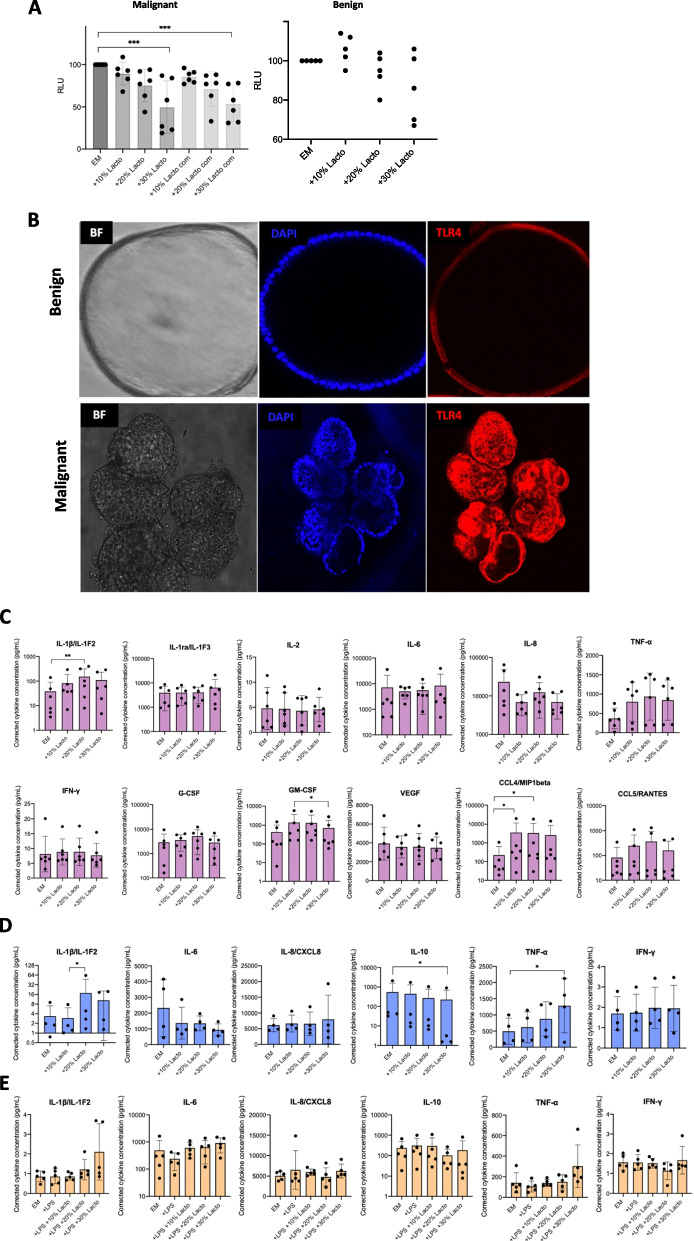
**A** Benign and endometrial cancer organoid viability in response to increasing *L. crispatus*- conditioned media concentrations (10%, 20%, 30% v/v) for 48 h. ***p value < 0.001 **B** Confocal microscopy in benign and malignant endometrial organoids stained for membrane-bound TLR-4 protein. **C** Cytokine expression by endometrial cancer organoids in response to increasing *L. crispatus*-conditioned media concentrations for 48 h. **D** Cytokine expression by benign endometrial organoids in response to increasing *L. crispatus*-conditioned media concentrations for 48 h. **E** Cytokine expression by benign endometrial organoids in response to LPS and increasing *L. crispatus*-conditioned media concentrations for 24 h. Assay LoD: 1-10 pg/ml. *EM: Expansion medium; Lacto: L. crispatus isolated from patient vaginal swab; Lacto com: commercial L. crispatus; BF: Brightfield.* *p value < 0.05 ** *p* value < 0.005; adjusted *p* values calculated by Dunn’s multiple comparisons test

Firstly, the viability of endometrial organoids was assessed at increasing concentrations of *L. crispatus-*conditioned (LCC) media (10%, 20%, 30%, 50% v/v). We noted that 50% v/v LCC was lethal across all endometrial organoid lines within 2 days (data not shown), and therefore used 10%, 20% and 30% v/v concentrations for further experiments. In EC organoids, proliferation showed an inverse relationship with increasing LCC concentrations at 48 h, irrespective of *L. crispatus* origin (*L. crispatus* vaginal isolate from a patient or commercial isolate), but this trend reached statistical significance only for the 30% v/v concentration (vaginal isolate: *p* =  0.0009; commercial isolate: *p* =  0.0003) (Fig. 6A). Benign endometrial organoids, on the other hand, demonstrated increased proliferation at 10% v/v LCC and decreased proliferation dynamics at 20% and 30% v/v concentrations, but relationships were not nominally significant (Fig. 6A).

### Effect on inflammation

We next investigated the impact of LCC media on the constitutive and LPS-induced cytokine secretion by endometrial organoids and found that *L. crispatus* supernatant does not significantly affect cytokine secretion in benign and malignant endometrial organoids. A set of ten cytokines (IL-1β, IL-1ra, IL-2, IL- 6, IL-8, G-CSF, GM-CSF, IFN-γ, TNF-α) and two chemokines (CCL4/MIP1beta, CCL5/RANTES), known to be secreted by endometrial tumours, were measured in endometrial cancer organoids and six cytokines (IL-1β, IL-6, IL-8, IL-10, IFN-γ, TNF-α), commonly secreted in response to inflammatory stimuli, were selected for benign organoids, which were left unstimulated or treated with LPS to mimic chronic inflammation that predisposes to endometrial carcinogenesis. Organoids were grown in an expansion medium supplemented with 10%, 20% and 30% v/v LCC medium and secreted cytokines were detected in culture supernatant after 48 h. Basal cytokine secretion of IL-1β, IL-6, IL-8 and TNF-α by malignant organoids was higher than benign organoids but only IFN-γ reached statistical significance (*p* =  0.0286) (Supplementary Figure S9B). For EC organoids, the addition of *L. crispatus* supernatant at any concentration did not significantly alter the basal secretion of IL-6, IL-8, TNF-α, IFN-γ, IL-1ra, IL-2, G-CSF, GM-CSF, VEGF and chemokine CCL5/RANTES, while a significant increase was noted for IL-1β and CCL4/MIP1beta at certain LCC concentrations (Fig. 6C). When endometrial cancer organoids were treated with a supernatant of commercially available *L. crispatus*, none of the cytokines/chemokines was significantly affected (Supplementary Figure S9C). Similarly, for benign endometrial organoids the secretion of IL-6, IL-8, IL-10, TNF-α and IFN-γ remained unaffected following LCC medium supplementation, whilst an increase of IL-1β and TNF-α and reduction of IL-10 production were observed at certain LCC concentrations (Fig. 6D). Finally, to mimic the chronic inflammatory signals predisposing to endometrial cancer, we simultaneously treated benign organoids, which express LPS receptor, TLR-4 (Toll-like receptor 4) (Fig. 6B), with *E. coli*- derived LPS (O111:B4) and increasing LCC media concentrations for 24 h. No significant changes were noted in the inducible secretion of IL-1β, IL-6, IL-8, IL-10, TNF-α and IFN-γ by benign organoids (Fig. 6E).

## Discussion

In this study, we sought to determine the presence of a genuine microbial fingerprint in the upper and lower female genital tract of benign and endometrial cancer patients highlighting potential differences and trying to elucidate the putative role of microbiota in endometrial cancer generation or suppression.

Contamination from the patient and non-patient sources may obscure actual microbial signatures from low-bacterial biomass sites. Our study integrated four different sets of technical controls for air and reagent/equipment contaminants during sample collection in the pathology laboratory, DNA extraction and 16S rRNA gene sequencing and their sequence reads (15% of total sequencing data) were removed to deduct background DNA contamination from further data analysis. Furthermore, we demonstrated potential intra-patient contamination in one-quarter to one-third of samples in laparoscopic procedures. A previous report described the high bacterial similarity of endometrial samples between transcervical and transuterine collection methods, indicating low contamination [28].

We calculated actual microbial abundances using quantitative real-time PCR and showed that endometrial, fallopian tube and ovarian microbiota are encountered in much lower quantities (2–4 log_10_) than vaginal and cervical microbiota and are similar to bacterial abundances of controls. Our findings are consistent with those of Mitchell and co-workers [18], who reported that mean bacterial quantities in the endometrium and upper endocervix are lower than vaginal levels by 2–4 log_10_ rRNA gene copies, which was further corroborated by the Chen study [28] showing that vaginal sites contained about four orders of magnitude more bacteria (10^10^–10^11^) than the endometrium and peritoneal fluid, still exhibiting much lower cycle threshold (Ct values) than negative controls. Another study confirmed high bacterial signals in the vagina and comparable bacterial load in the cervix and endometrium with most cervical and endometrial samples (60–72%) exceeding the bacterial copies of blank controls [17].

Overall, we observed a microbiota signature above background contamination in 62% of benign endometrium, 50% of malignant endometrium, 85% of benign fallopian tube and 95% of benign ovary based on sequencing reads. Winters and colleagues also reported a 60% (15/25) bacterial recovery above background DNA controls from the endometrium of women with benign pathology, mainly fibroids, undergoing transabdominal hysterectomy [17]. Mitchell and co-workers concluded that 95% (55/58) of non-cancer patients having total laparoscopic or laparoscopically-assisted vaginal hysterectomy without an intracervical manipulator had endometrial colonisation with at least one species [18], while Chen and colleagues enrolled 95 reproductive age women and isolated bacteria from the endometrium, fallopian tubes and peritoneal fluid during either laparotomy or laparoscopy [28]. With regards to bacterial composition, we identified a prominent microbial signature in benign endometrium, fallopian tube and ovary, while the microbiome of malignant endometrial samples and technical controls was largely shared. In the Mitchell and co-workers study, signature endometrial species were *L. iners* (45%), *Prevotella* spp. (33%) and *L. crispatus* (33%) [18], while in the Chen study *Lactobacillus, Vagococcus, Acinetobacter* and *Pseudomonas*, which were also identified in the fallopian tube[28]. Notably, our results are predominantly deduced from post-menopausal women >  50 years of age, while the aforementioned reports involved mainly premenopausal women <  50 years of age. The data suggests that a microbiota signature is detectable in the upper reproductive tract of a proportion of women regardless of age and menopausal status.

Investigation of a microbial continuum within the female genital tract revealed that in 75% of benign patients, the most abundant species of the lower genital tract were also recoverable from all sites of the upper genital tract, lending credence to the theory of bacterial colonisation through ascension for certain bacteria. The bacterial continuum displayed moderate to low cohesion for most patients in the endometrial cancer cohort. Intra-individual microbial correlations between the lower and upper genital tract consistent with anatomical contingency have been reported by Chen and colleagues [28], whereas Walsh and co-workers reported that the microbiome of the lower genital tract is correlated with the uterine microbiome in patients with endometrial cancer [10]. In addition to this, associations of vaginal with rectal microbiota have been suggested based on reports of bacterial species overlap between the two sites and the presence of enteric oestrobolome, where the gut microbiome metabolises oestrogens and indirectly facilitates vaginal *Lactobacillus* growth [41–47]. We found that vaginal microbiota correlated poorly with rectal microbiota in both benign and endometrial cancer patients displaying only 5–11% sharedness.

We subsequently took cross-sectional snapshots of the FGT microbial structure in thirty-seven women and twenty-four women without endometrial cancer. Our findings suggest that there is an unequivocal decrease of *Lactobacillus* abundance in the vagina, cervix and endometrium of endometrial cancer patients and an enrichment of *Anaerococcus*, *Porphyromonas*, *Prevotella, Fusobacterium, Bacteroides* and *Peptoniphilus*, which is in keeping with previous studies [10, 11]. *Lactobacillus* reduction lends support to the more broadly described association between *Lactobacillus* depletion and adverse gynaecological and obstetric outcomes*,* including cervical cancer and precancerous lesions [13] as well as miscarriages [48], preterm labour [49] and in vitro fertilisation failure [24]. The biological significance of *Lactobacillus* communities includes pathogen antagonism by lactic acid, hydrogen peroxide and bacteriocin production but also immune and metabolic pathways as well as epigenetic regulation[50–59]. Anti-cancer effects of *Lactobacillus* species have been reported for *L. casei*, *L. plantarum*, *L. rhamnosus* and *L. acidophilus* through natural killer cell activation, dendritic cell maturation or probiotic-derived ferrichrome release [60–68] inducing cancer cell apoptosis in vitro [61, 67, 69] and increasing anticancer drug efficacy [70, 71

We sought to determine whether the disparities observed between benign and endometrial cancer patient microbiota can be attributed to malignancy, suggesting a cause-effect relationship or other confounders impacted our findings. Ethnicity [70], menopausal status [10, 71], obesity [72], use of sex hormones [71], smoking [73], sexual behaviour [74] and vaginal pH [11] have all been recognised as modulators of microbial composition in the female genital tract. Our group has previously demonstrated that obesity drives the vaginal microbiome towards a highly diverse, *Lactobacillus*-depleted eco-structure characterised by higher levels of *Dialister*, *Anaerococcus vaginalis* and *Prevotella timonensis*, and lower levels of *Lactobacillus crispatus* compared to non-obese women [72]. Another study highlighted the importance of menopausal status in FGT composition showing that postmenopausal women exhibit increased diversity and enrichment of *Anaerococcus*, *Peptoniphilus* and *Porphyromonas* species [10]. Interestingly, almost half (8/17) of the microbes enriched in postmenopausal women were also associated with endometrial cancer [10]. From a mechanistic standpoint, the interplay between estrogens and *Lactobacilli* is well-documented in the vaginal mucosa with oestrogens stimulating glycogen-dependent metabolism that increases colonisation with lactobacilli, which in turn metabolise glycogen to lactic acid maintaining an acidic vaginal environment [75]. In our study, no variable showed systematic bias with respect to the two comparison groups (benign vs endometrial cancer) apart from age; endometrial cancer patients were predominantly ≥  65 years and menopausal for longer than a decade, while benign patients between 50 and 64 years of age and more recently menopausal. It is possible that Lactobacillus depletion observed in EC is partly due to differences in the age range and hormonal changes between cohorts. Even though we performed sub-analysis adjusted for age which confirmed microbial trends as presented in this manuscript, sample size in each cohort was further reduced and had a substantial impact on statistical significance tests.

To uncover a potential role of altered microbiota in endometrial carcinogenesis, we selected *L. crispatus* as a candidate health-promoting microbe, given that it is significantly depleted in endometrial cancer and substantial evidence suggests that commensal *Lactobacillus* species have anti-inflammatory and anti-cancer properties [54–69]. Endometrial cancer is thought to arise in an environment of chronic, low-grade inflammation characterised by an increase of pre-diagnostic circulating levels of CRP, IL-6, TNFα, sTNFR1 and sTNFR2 and IL-1Ra (EPIC cohort) [76, 77], which have also been linked to high BMI [78, 79], highlighting the interrelationship of the obesity- inflammation- endometrial carcinogenesis axis. Following the establishment of endometrial cancer, cytokines are secreted by cancer cells, the tumour microenvironment and infiltrating immune cells but their role in oncogenic processes remains ambiguous. We interrogated the effect of *L. crispatus* on endometrial cell proliferation and its intersection with inflammatory pathways and found that *L. crispatus*-conditioned medium reduced the viability of endometrial cancer organoids, whilst having minimal effect on studied cytokines/chemokines. It is not clear whether the LCC medium is actually cytotoxic or restricts proliferation and/or induces lethality of endometrial organoids due to dilutional effects or other molecular mechanisms.

A limitation of this study involves the use of women with benign pathology as controls instead of healthy individuals who may display differential microbial structure. Women undergoing risk-reducing surgery can provide an ethically acceptable source of healthy surgical specimens in future studies. Longitudinal studies monitoring the bacterial composition intra-individually over time to capture the transition from a healthy state to precancerous conditions (complex atypical hyperplasia) and invasive endometrial carcinoma are required to provide useful information on microbiota dynamics and temporal shifts within each patient. Furthermore, viral and fungal metagenomic studies could be useful to complement and expand our knowledge of the human-microbial and microbial-microbial cell interactions in health and disease. Finally, qPCR data of the 16S rRNA gene in our study were not normalised to 16S gene copies for each species because significant variation occurs even between bacterial strains and our study design lacked strain data that would allow such analysis.

## Conclusions

In conclusion, only a subset of women with and without endometrial cancer harbours a microbiome in the upper GT that is quantitatively and qualitatively distinct from background contamination. *Lactobacillus* depletion in the lower and upper GT is characteristic of endometrial malignancy but whether depletion precedes or follows the development of cancer is still unclear. *L. crispatus* does not significantly affect cytokine production in benign and malignant endometrial organoids but this observation does not preclude that *L. crispatus* or other bacterial species could influence metabolic or oestrogen signalling pathways locally or could have an impact on infiltrating immune cell cytokine secretion, which could subsequently exert an action on endometrium. Transitioning from reductionist cell models to more comprehensive, physiologically relevant cell systems that capture the cellular diversity of organs, including stromal, mesenchymal and immune cells, is mandatory to reliably assess the functional role of resident microbiota and devise ways of manipulation that could restore eubiosis. Unravelling microbial signatures in disease and the intricate mechanisms through which resident microbiota influence cellular functions bears a substantial translational value with regard to cancer prevention and early diagnosis as well as microbiome-based therapeutics. Knowledge from dysbiotic microbiota could identify patients at risk of developing endometrial cancer leading to increased surveillance, whilst modulation of microbiota with pre- and probiotics or microbiota transplantation could potentially reverse the carcinogenic process.

### Supplementary Information

**Additional file 1:  Supplementary Table S1.** Potential (de)contamination sources, samples affected and management.**Supplementary Table S2.** OTUs removed as contaminants. **Supplementary Table S3.** Samples with low read counts not included in analysis. **Supplementary Table S4.** Recruits for organoid experiments. **Supplementary Figure S1.** 16S rRNA gene sequence read counts pre- and post-removal of contaminant sequence reads. Lines represent mean with SD. **Supplementary Figure S2.** Swab-tissue pairwise comparison of microbial yield and composition in different anatomic sites of benign and endometrial cancer patients (genera). **Supplementary Figure S3**. Comparison between laparoscopic and transabdominal procedures to determine potential contamination of low-biomass sites in laparoscopy during transcervical insertion of uterine manipulator and vaginal retrieval of surgical specimen. **Supplementary Figure S4.** Microbiota continuum in lower and upper female genital tract. **Supplementary Figure S5.** Intra-individual correlation between vaginal-rectal microbiota. **Supplementary Figure S6.** Shannon α-diversity among microbial clusters identified in different anatomical sites (species). **Supplementary Figure S7.** Microbiome Shannon α- and β-diversity according to histological type and grade of endometrial cancer per anatomical site (genera). **Supplementary Figure S8.** Comparison of microbial composition in the rectum of women with and without endometrial cancer. **Supplementary Figure S9.** A. Benign organoid viability in response to LPS (1μg/mL, E. coli O111:B4) and L. crispatus- conditioned media for 24h or MRS broth for 48h. Organoid proliferation was significantly reduced in co-incubation of LPS and 30% LCC (*p*= 0.0097) but unaltered when LPS alone was used or combined with other LCC concentrations (10%, 20% v/v). MRS broth alone significantly decreased proliferation in the 30% v/v concentration (*p*= 0.027).  B. Comparison of basal cytokine secretion by benign and endometrial cancer organoids after 48h of culture. C. Cytokine secretion by endometrial cancer organoids in response to increasing L. crispatus-conditioned media concentrations for 48h. Assay LoD: 1-10pg/ml. RLU: Relative Light Unit; Lacto com: commercial L. crispatus; LPS: Lipopolysaccharide; MRS: MRS broth; Ben: Benign; Mal: Malignant.  * *p*-value < 0.05, ** *p*-value < 0.01; adjusted *p* values calculated by Dunn’s multiple comparisons test.

## Data Availability

All data generated or analysed during this study are included in this published article and its supplementary information files.
