# Supplementary material for: Microbial signatures and continuum in endometrial cancer and benign patients
Source: Microbiome. 2024 Jul 1;12:118. doi: 10.1186/s40168-024-01821-0 (PMC11218081; doi:10.1186/s40168-024-01821-0)
Supplement: Supplementary file 2 — Additional file 1: Supplementary Table S1. Potential (de)contamination sources, samples affected and management.Supplementary Table S2. OTUs removed as contaminants. Supplementary Table S3. Samples with low read counts not included in analysis. Supplementary Table S4. Recruits for organoid experiments. Supplementary Figure S1. 16S rRNA gene sequence read counts pre- and post-removal of contaminant sequence reads. Lines represent mean with SD. Supplementary Figure S2. Swab-tissue pairwise comparison of microbial yield and composition in different anatomic sites of benign and endometrial cancer patients (genera). Supplementary Figure S3. Comparison between laparoscopic and transabdominal procedures to determine potential contamination of low-biomass sites in laparoscopy during transcervical insertion of uterine manipulator and vaginal retrieval of surgical specimen. Supplementary Figure S4. Microbiota continuum in lower and upper female genital tract. Supplementary Figure S5. Intra-individual correlation between vaginal-rectal microbiota. Supplementary Figure S6. Shannon α-diversity among microbial clusters identified in different anatomical sites (species). Supplementary Figure S7. Microbiome Shannon α- and β-diversity according to histological type and grade of endometrial cancer per anatomical site (genera). Supplementary Figure S8. Comparison of microbial composition in the rectum of women with and without endometrial cancer. Supplementary Figure S9. A. Benign organoid viability in response to LPS (1μg/mL, E. coli O111:B4) and L. crispatus- conditioned media for 24h or MRS broth for 48h. Organoid proliferation was significantly reduced in co-incubation of LPS and 30% LCC (p= 0.0097) but unaltered when LPS alone was used or combined with other LCC concentrations (10%, 20% v/v). MRS broth alone significantly decreased proliferation in the 30% v/v concentration (p= 0.027). B. Comparison of basal cytokine secretion by benign and endometrial cancer organoids after 48h [file 40168_2024_1821_MOESM1_ESM.docx]

**Supplementary material**

**Table 1.** Potential (de)contamination sources, samples affected and management

| **Sources of (de)contamination** | **Samples affected** | **Management** |
| --- | --- | --- |
| Air | All | Controls included |
| Pre-op enema | Rectal | Unknown effect on rectal microbiome |
| **In theatre** | | |
| Intra-op antibiotics | Endometrial, fallopian tube, ovarian | Administered after collection of vaginal, cervical, rectal samples  Dead bacteria still picked up by 16S rRNA gene sequencing |
| Uterine manipulator in laparoscopic procedures | Endometrial | Comparison with transabdominal hysterectomies |
| Vaginal retrieval of specimen in laparoscopic procedures | Fallopian tube, ovarian | Vaginal disinfection pre-op  Comparison with transabdominal hysterectomies |
| **In the pathology lab** | | |
| Knife for uterus dissection | Endometrial | Controls included |
| **In the lab** | | |
| DNA extraction kit | All | Negative controls included |
| 16S rRNA gene sequencing | All | Negative controls included |
| Sample carryover during DNA extraction/sequencing | All | Layout of sample loading taken into account during analysis |

**Table 2.** OTUs removed as contaminants

| **OTU** | **Phylum** | **Class** | **Order** | **Family** | **Genera** | **Species** |
| --- | --- | --- | --- | --- | --- | --- |
| Otu00013 | Bacteroidetes | Bacteroidia | Bacteroidales | Prevotellaceae | Prevotella | Prevotella_copri |
| Otu00020 | Firmicutes | Bacilli | Lactobacillales | Lactobacillaceae | Lactobacillus | Lactobacillus_fornicalis |
| Otu00024 | Bacteroidetes | Bacteroidia | Bacteroidales | Prevotellaceae | Prevotella | Prevotella_copri |
| Otu00036 | Proteobacteria | Gammaproteobacteria | Pseudomonadales | Moraxellaceae | Psychrobacter | Psychrobacter_vallis |
| Otu00055 | Actinobacteria | Actinobacteria | Actinomycetales | Propionibacteriaceae | Propionibacterium | Propionibacterium_acnes |
| Otu00066 | Bacteroidetes | Bacteroidia | Bacteroidales | Prevotellaceae | Massiliprevotella | Massiliprevotella_massiliensis |
| Otu00070 | Firmicutes | Erysipelotrichia | Erysipelotrichales | Erysipelotrichaceae | Catenibacterium | Catenibacterium_mitsuokai |
| Otu00076 | Proteobacteria | Betaproteobacteria | Rhodocyclales | Rhodocyclaceae | Georgfuchsia | Georgfuchsia_unclassified |
| Otu00078 | Proteobacteria | Alphaproteobacteria | Rhizobiales | Methylobacteriaceae | Methylobacterium | Methylobacterium_extorquens |
| Otu00079 | Firmicutes | Clostridia | Clostridiales | Ruminococcaceae | Butyricicoccus | Butyricicoccus_unclassified |
| Otu00082 | Bacteroidetes | Bacteroidia | Bacteroidales | Prevotellaceae | Prevotella | Prevotella_copri |
| Otu00092 | Firmicutes | Bacilli | Bacillales | Staphylococcaceae | Staphylococcus | Staphylococcus_epidermidis |
| Otu00098 | Proteobacteria | Betaproteobacteria | Burkholderiales | Oxalobacteraceae | Janthinobacterium | Janthinobacterium_lividum |
| Otu00103 | Proteobacteria | Betaproteobacteria | Burkholderiales | Oxalobacteraceae | Duganella | Duganella_zoogloeoides |
| Otu00111 | Firmicutes | Clostridia | Clostridiales | Lachnospiraceae | Coprococcus | Coprococcus_eutactus |
| Otu00113 | Proteobacteria | Betaproteobacteria | Burkholderiales | Oxalobacteraceae | Duganella | Duganella_phyllosphaerae |
| Otu00124 | Bacteroidetes | Bacteroidia | Bacteroidales | Prevotellaceae | Prevotella | Prevotella_unclassified |
| Otu00134 | Firmicutes | Erysipelotrichia | Erysipelotrichales | Erysipelotrichaceae | Clostridium XVIII | Clostridium XVIII_unclassified |
| Otu00139 | Firmicutes | Clostridia | Clostridiales | Lachnospiraceae | Blautia | Blautia_faecis |
| Otu00149 | Proteobacteria | Alphaproteobacteria | Rhizobiales | Rhizobiaceae | Rhizobium | Rhizobium_radiobacter |
| Otu00150 | Firmicutes | Clostridia | Clostridiales | Ruminococcaceae | Gemmiger | Gemmiger_formicilis |
| Otu00152 | Proteobacteria | Betaproteobacteria | Burkholderiales | Comamonadaceae | Rhodoferax | Rhodoferax_ferrireducens |
| Otu00154 | Firmicutes | Clostridia | Clostridiales | Lachnospiraceae | Blautia | Blautia_luti |
| Otu00156 | Firmicutes | Bacilli | Bacillales | Planococcaceae | Sporosarcina | Sporosarcina_globispora |
| Otu00158 | Firmicutes | Clostridia | Clostridiales | Lachnospiraceae | Coprococcus | Coprococcus_unclassified |
| Otu00160 | Actinobacteria | Actinobacteria | Actinomycetales | Corynebacteriaceae | Corynebacterium | Corynebacterium_faecale |
| Otu00163 | Proteobacteria | Betaproteobacteria | Burkholderiales | Comamonadaceae | Rhodoferax | Rhodoferax_ferrireducens |
| Otu00169 | Proteobacteria | Betaproteobacteria | Burkholderiales | Comamonadaceae | Polaromonas | Polaromonas_jejuensis |
| Otu00179 | Actinobacteria | Actinobacteria | Actinomycetales | Streptomycetaceae | Streptomyces | Streptomyces_albidoflavus |
| Otu00187 | Proteobacteria | Alphaproteobacteria | Rhizobiales | Brucellaceae | Ochrobactrum | Ochrobactrum_pseudogrignonense |
| Otu00188 | Proteobacteria | Betaproteobacteria | Burkholderiales | Comamonadaceae | Delftia | Delftia_lacustris |
| Otu00191 | Actinobacteria | Actinobacteria | Bifidobacteriales | Bifidobacteriaceae | Bifidobacterium | Bifidobacterium_adolescentis |
| Otu00196 | Proteobacteria | Betaproteobacteria | Burkholderiales | Comamonadaceae | Polaromonas | Polaromonas_unclassified |
| Otu00198 | Proteobacteria | Gammaproteobacteria | Pseudomonadales | Moraxellaceae | Acinetobacter | Acinetobacter_pakistanensis |
| Otu00199 | Proteobacteria | Gammaproteobacteria | Pseudomonadales | Pseudomonadaceae | Pseudomonas | Pseudomonas_asturiensis |
| Otu00201 | Bacteroidetes | Sphingobacteriia | Sphingobacteriales | Sphingobacteriaceae | Pedobacter | Pedobacter_cryoconitis |
| Otu00205 | Proteobacteria | Betaproteobacteria | Burkholderiales | Sutterellaceae | Sutterella | Sutterella_wadsworthensis |
| Otu00206 | Firmicutes | Negativicutes | Selenomonadales | Veillonellaceae | Megamonas | Megamonas_funiformis |
| Otu00209 | Actinobacteria | Actinobacteria | Actinomycetales | Micrococcaceae | Arthrobacter | Arthrobacter_antarcticus |
| Otu00212 | Proteobacteria | Betaproteobacteria | Burkholderiales | Oxalobacteraceae | Herbaspirillum | Herbaspirillum_rubrisubalbicans |
| Otu00226 | Proteobacteria | Gammaproteobacteria | Pseudomonadales | Pseudomonadaceae | Rugamonas | Rugamonas_rubra |
| Otu00229 | Firmicutes | Clostridia | Clostridiales | Lachnospiraceae | Roseburia | Roseburia_intestinalis |
| Otu00230 | Firmicutes | Bacilli | Lactobacillales | Carnobacteriaceae | Trichococcus | Trichococcus_pasteurii |
| Otu00235 | Proteobacteria | Gammaproteobacteria | Pseudomonadales | Pseudomonadaceae | Pseudomonas | Pseudomonas_caeni |
| Otu00239 | Bacteroidetes | Bacteroidia | Bacteroidales | Bacteroidaceae | Bacteroides | Bacteroides_eggerthii |
| Otu00240 | Firmicutes | Clostridia | Clostridiales | Lachnospiraceae | Clostridium XlVa | Clostridium XlVa_unclassified |
| Otu00241 | Firmicutes | Clostridia | Clostridiales | Ruminococcaceae | Oscillibacter | Oscillibacter_unclassified |
| Otu00242 | Actinobacteria | Actinobacteria | Actinomycetales | Microbacteriaceae | Cryobacterium | Cryobacterium_arcticum |
| Otu00247 | Actinobacteria | Actinobacteria | Actinomycetales | Microbacteriaceae | Agreia | Agreia_pratensis |
| Otu00251 | Firmicutes | Clostridia | Clostridiales | Clostridiaceae 1 | Clostridium sensu stricto | Clostridium_tagluense |
| Otu00255 | Proteobacteria | Betaproteobacteria | Burkholderiales | Comamonadaceae | Acidovorax | Acidovorax_facilis |
| Otu00258 | Actinobacteria | Actinobacteria | Actinomycetales | Corynebacteriaceae | Corynebacterium | Corynebacterium_efficiens |
| Otu00260 | Firmicutes | Clostridia | Clostridiales | Lachnospiraceae | Clostridium XlVa | Clostridium XlVa_unclassified |
| Otu00265 | Proteobacteria | Gammaproteobacteria | Enterobacteriales | Enterobacteriaceae | Klebsiella | Kosakonia_sacchari |
| Otu00275 | Proteobacteria | Betaproteobacteria | Burkholderiales | Comamonadaceae | Pseudorhodoferax | Pseudorhodoferax_soli |
| Otu00283 | Firmicutes | Bacilli | Bacillales | Planococcaceae | Planococcus | Planococcus_maitriensis |
| Otu00294 | Proteobacteria | Betaproteobacteria | Burkholderiales | Comamonadaceae | Giesbergeria | Giesbergeria_giesbergeri |
| Otu00299 | Proteobacteria | Betaproteobacteria | Burkholderiales | Comamonadaceae | Simplicispira | Simplicispira_psychrophila |
| Otu00304 | Firmicutes | Clostridia | Clostridiales | Ruminococcaceae | Oscillibacter | Oscillibacter_unclassified |
| Otu00305 | Actinobacteria | Actinobacteria | Actinomycetales | Demequinaceae | Lysinimicrobium | Lysinimicrobium_unclassified |
| Otu00306 | Actinobacteria | Actinobacteria | Actinomycetales | Nocardiaceae | Rhodococcus | Rhodococcus_qingshengii |
| Otu00312 | Bacteroidetes | Bacteroidia | Bacteroidales | Bacteroidaceae | Bacteroides | Bacteroides_faecis |
| Otu00318 | Proteobacteria | Betaproteobacteria | Burkholderiales | Burkholderiales_incertae_sedis | Sphaerotilus | Sphaerotilus_montanus |
| Otu00323 | Firmicutes | Clostridia | Clostridiales | Lachnospiraceae | Eisenbergiella | Eisenbergiella_unclassified |
| Otu00329 | Bacteroidetes | Cytophagia | Cytophagales | Cytophagaceae | Dyadobacter | Dyadobacter_unclassified |
| Otu00331 | Firmicutes | Erysipelotrichia | Erysipelotrichales | Erysipelotrichaceae | Erysipelotrichaceae_incertae_sedis | Erysipelotrichaceae_incertae_sedis_unclassified |
| Otu00339 | Firmicutes | Bacilli | Lactobacillales | Aerococcaceae | Facklamia | Facklamia_unclassified |
| Otu00341 | Firmicutes | Bacilli | Bacillales | Planococcaceae | Planococcus | Planococcus_antarcticus |
| Otu00343 | Firmicutes | Clostridia | Clostridiales | Clostridiaceae 1 | Clostridium sensu stricto | Clostridium sensu stricto_unclassified |
| Otu00345 | Proteobacteria | Alphaproteobacteria | Rhizobiales | Hyphomicrobiaceae | Devosia | Devosia_yakushimensis |
| Otu00358 | Cyanobacteria/Chloroplast | Chloroplast | Chloroplast | Chloroplast | Bacillariophyta | Bacillariophyta_unclassified |
| Otu00359 | Bacteroidetes | Flavobacteriia | Flavobacteriales | Flavobacteriaceae | Flavobacterium | Flavobacterium_noncentrifugens |
| Otu00363 | Proteobacteria | Gammaproteobacteria | Pseudomonadales | Pseudomonadaceae | Serpens | Serpens_unclassified |
| Otu00380 | Firmicutes | Bacilli | Lactobacillales | Carnobacteriaceae | Trichococcus | Trichococcus_pasteurii |
| Otu00384 | Proteobacteria | Betaproteobacteria | Burkholderiales | Comamonadaceae | Hydrogenophaga | Hydrogenophaga_caeni |
| Otu00390 | Proteobacteria | Alphaproteobacteria | Caulobacterales | Caulobacteraceae | Brevundimonas | Brevundimonas_staleyi |
| Otu00393 | Proteobacteria | Betaproteobacteria | Burkholderiales | Comamonadaceae | Variovorax | Variovorax_ginsengisoli |
| Otu00405 | Bacteroidetes | Sphingobacteriia | Sphingobacteriales | Saprospiraceae | Portibacter | Portibacter_unclassified |
| Otu00413 | Actinobacteria | Actinobacteria | Bifidobacteriales | Bifidobacteriaceae | Bifidobacterium | Bifidobacterium_adolescentis |
| Otu00414 | Bacteroidetes | Flavobacteriia | Flavobacteriales | Flavobacteriaceae | Flavobacterium | Flavobacterium_aquatile |
| Otu00421 | Firmicutes | Bacilli | Bacillales | Bacillales_Incertae Sedis XI | Gemella | Gemella_haemolysans |
| Otu00423 | Proteobacteria | Betaproteobacteria | Burkholderiales | Comamonadaceae | Rhodoferax | Rhodoferax_saidenbachensis |
| Otu00425 | Firmicutes | Clostridia | Clostridiales | Lachnospiraceae | Blautia | Blautia_faecis |
| Otu00429 | Proteobacteria | Alphaproteobacteria | Caulobacterales | Caulobacteraceae | Caulobacter | Caulobacter_henricii |
| Otu00431 | Firmicutes | Clostridia | Clostridiales | Ruminococcaceae | Butyricicoccus | Butyricicoccus_unclassified |
| Otu00432 | Firmicutes | Bacilli | Lactobacillales | Lactobacillaceae | Lactobacillus | Lactobacillus_gasseri |
| Otu00433 | Proteobacteria | Alphaproteobacteria | Rhizobiales | Bradyrhizobiaceae | Bradyrhizobium | Bradyrhizobium_jicamae |
| Otu00440 | Firmicutes | Bacilli | Bacillales | Planococcaceae | Lysinibacillus | Lysinibacillus_fusiformis |
| Otu00441 | Bacteroidetes | Flavobacteriia | Flavobacteriales | Flavobacteriaceae | Flavobacterium | Flavobacterium_piscis |
| Otu00442 | Bacteroidetes | Sphingobacteriia | Sphingobacteriales | Sphingobacteriaceae | Pedobacter | Pedobacter_hartonius |
| Otu00448 | Proteobacteria | Betaproteobacteria | Burkholderiales | Burkholderiaceae | Ralstonia | Ralstonia_insidiosa |
| Otu00454 | Bacteroidetes | Flavobacteriia | Flavobacteriales | Flavobacteriaceae | Flavobacterium | Flavobacterium_unclassified |
| Otu00456 | Firmicutes | Clostridia | Clostridiales | Peptococcaceae 1 | Peptococcus | Peptococcus_unclassified |
| Otu00458 | Proteobacteria | Epsilonproteobacteria | Campylobacterales | Campylobacteraceae | Arcobacter | Arcobacter_venerupis |
| Otu00460 | Proteobacteria | Betaproteobacteria | Burkholderiales | Comamonadaceae | Rhodoferax | Rhodoferax_saidenbachensis |
| Otu00462 | Firmicutes | Bacilli | Lactobacillales | Carnobacteriaceae | Carnobacterium | Carnobacterium_jeotgali |
| Otu00465 | Firmicutes | Clostridia | Clostridiales | Clostridiaceae 1 | Clostridium sensu stricto | Clostridium_estertheticum |
| Otu00466 | Bacteroidetes | Flavobacteriia | Flavobacteriales | Flavobacteriaceae | Flavobacterium | Flavobacterium_piscis |
| Otu00467 | Proteobacteria | Epsilonproteobacteria | Campylobacterales | Campylobacteraceae | Arcobacter | Arcobacter_unclassified |
| Otu00475 | Proteobacteria | Gammaproteobacteria | Pseudomonadales | Pseudomonadaceae | Rhizobacter | Rhizobacter_dauci |
| Otu00484 | Proteobacteria | Gammaproteobacteria | Pseudomonadales | Pseudomonadaceae | Pseudomonas | Pseudomonas_mandelii |
| Otu00485 | Firmicutes | Clostridia | Clostridiales | Ruminococcaceae | Flavonifractor | Flavonifractor_unclassified |
| Otu00489 | Firmicutes | Clostridia | Clostridiales | Clostridiales_Incertae Sedis XIII | Anaerovorax | Anaerovorax_unclassified |
| Otu00490 | Proteobacteria | Epsilonproteobacteria | Campylobacterales | Campylobacteraceae | Arcobacter | Arcobacter_cryaerophilus |
| Otu00498 | Proteobacteria | Betaproteobacteria | Burkholderiales | Sutterellaceae | Sutterella | Sutterella_stercoricanis |
| Otu00502 | Proteobacteria | Alphaproteobacteria | Rhizobiales | Rhizobiaceae | Rhizobium | Rhizobium_lusitanum |
| Otu00511 | Proteobacteria | Gammaproteobacteria | Pseudomonadales | Pseudomonadaceae | Serpens | Serpens_unclassified |
| Otu00513 | Firmicutes | Bacilli | Lactobacillales | Streptococcaceae | Lactococcus | Lactococcus_lactis |
| Otu00519 | Actinobacteria | Actinobacteria | Actinomycetales | Microbacteriaceae | Agreia | Agreia_unclassified |
| Otu00523 | Firmicutes | Clostridia | Clostridiales | Lachnospiraceae | Lachnospiracea_incertae_sedis | Lachnospiracea_incertae_sedis_unclassified |
| Otu00530 | Actinobacteria | Actinobacteria | Actinomycetales | Microbacteriaceae | Conyzicola | Conyzicola_lurida |
| Otu00531 | Proteobacteria | Betaproteobacteria | Burkholderiales | Oxalobacteraceae | Janthinobacterium | Janthinobacterium_unclassified |
| Otu00533 | Firmicutes | Clostridia | Clostridiales | Lachnospiraceae | Roseburia | Roseburia_unclassified |
| Otu00538 | Bacteroidetes | Flavobacteriia | Flavobacteriales | Flavobacteriaceae | Flavobacterium | Flavobacterium_segetis |
| Otu00540 | Actinobacteria | Actinobacteria | Actinomycetales | Propionibacteriaceae | Propionibacterium | Propionibacterium_acnes |
| Otu00544 | Firmicutes | Bacilli | Bacillales | Staphylococcaceae | Jeotgalicoccus | Jeotgalicoccus_psychrophilus |
| Otu00549 | Fusobacteria | Fusobacteriia | Fusobacteriales | Fusobacteriaceae | Cetobacterium | Cetobacterium_unclassified |
| Otu00556 | Actinobacteria | Actinobacteria | Actinomycetales | Kineosporiaceae | Kineosporia | Kineosporia_rhamnosa |
| Otu00557 | Firmicutes | Bacilli | Lactobacillales | Lactobacillaceae | Lactobacillus | Lactobacillus_paracasei |
| Otu00559 | Proteobacteria | Betaproteobacteria | Burkholderiales | Comamonadaceae | Polaromonas | Polaromonas_jejuensis |
| Otu00561 | Proteobacteria | Alphaproteobacteria | Sphingomonadales | Sphingomonadaceae | Novosphingobium | Novosphingobium_aromaticivorans |
| Otu00564 | Proteobacteria | Gammaproteobacteria | Pseudomonadales | Pseudomonadaceae | Pseudomonas | Pseudomonas_caeni |
| Otu00567 | Firmicutes | Clostridia | Clostridiales | Clostridiales_Incertae Sedis XI | Anaerococcus | Anaerococcus_unclassified |
| Otu00568 | Firmicutes | Clostridia | Clostridiales | Clostridiales_Incertae Sedis XI | Parvimonas | Parvimonas_micra |
| Otu00569 | Firmicutes | Bacilli | Lactobacillales | Enterococcaceae | Enterococcus | Enterococcus_aquimarinus |
| Otu00573 | Proteobacteria | Alphaproteobacteria | Caulobacterales | Caulobacteraceae | Caulobacter | Caulobacter_vibrioides |
| Otu00575 | Bacteroidetes | Flavobacteriia | Flavobacteriales | Flavobacteriaceae | Flavobacterium | Flavobacterium_tiangeerense |
| Otu00578 | Proteobacteria | Alphaproteobacteria | Rhizobiales | Methylobacteriaceae | Methylobacterium | Methylobacterium_adhaesivum |
| Otu00581 | Bacteroidetes | Sphingobacteriia | Sphingobacteriales | Sphingobacteriaceae | Pedobacter | Pedobacter_alluvionis |
| Otu00589 | Proteobacteria | Betaproteobacteria | Burkholderiales | Oxalobacteraceae | Undibacterium | Undibacterium_parvum |
| Otu00592 | Firmicutes | Negativicutes | Selenomonadales | Veillonellaceae | Allisonella | Allisonella_histaminiformans |
| Otu00597 | Proteobacteria | Alphaproteobacteria | Sphingomonadales | Sphingomonadaceae | Sphingomonas | Sphingomonas_aurantiaca |
| Otu00601 | Proteobacteria | Betaproteobacteria | Burkholderiales | Oxalobacteraceae | Duganella | Duganella_phyllosphaerae |
| Otu00603 | Proteobacteria | Alphaproteobacteria | Sphingomonadales | Sphingomonadaceae | Sphingomonas | Sphingomonas_aerolata |
| Otu00614 | Proteobacteria | Alphaproteobacteria | Rhizobiales | Rhizobiaceae | Shinella | Shinella_curvata |
| Otu00620 | Bacteroidetes | Sphingobacteriia | Sphingobacteriales | Sphingobacteriaceae | Pedobacter | Pedobacter_duraquae |
| Otu00621 | Proteobacteria | Betaproteobacteria | Neisseriales | Neisseriaceae | Rivicola | Rivicola_unclassified |
| Otu00622 | Bacteroidetes | Flavobacteriia | Flavobacteriales | Flavobacteriaceae | Chryseobacterium | Chryseobacterium_psychrotolerans |
| Otu00624 | Actinobacteria | Actinobacteria | Bifidobacteriales | Bifidobacteriaceae | Bifidobacterium | Bifidobacterium_stercoris |
| Otu00626 | Actinobacteria | Actinobacteria | Actinomycetales | Nakamurellaceae | Nakamurella | Nakamurella_unclassified |
| Otu00627 | Bacteroidetes | Sphingobacteriia | Sphingobacteriales | Sphingobacteriaceae | Pedobacter | Pedobacter_alluvionis |
| Otu00629 | Firmicutes | Clostridia | Clostridiales | Clostridiaceae 1 | Clostridium sensu stricto | Clostridium_lacusfryxellense |
| Otu00630 | Proteobacteria | Epsilonproteobacteria | Campylobacterales | Campylobacteraceae | Arcobacter | Arcobacter_unclassified |
| Otu00631 | Actinobacteria | Actinobacteria | Coriobacteriales | Coriobacteriaceae | Slackia | Slackia_isoflavoniconvertens |
| Otu00632 | Proteobacteria | Betaproteobacteria | Neisseriales | Neisseriaceae | Neisseria | Neisseria_perflava |
| Otu00634 | Bacteroidetes | Sphingobacteriia | Sphingobacteriales | Sphingobacteriaceae | Mucilaginibacter | Mucilaginibacter_rigui |
| Otu00636 | Actinobacteria | Actinobacteria | Actinomycetales | Micromonosporaceae | Actinoplanes | Actinoplanes_nipponensis |
| Otu00642 | Actinobacteria | Actinobacteria | Actinomycetales | Nocardiaceae | Nocardia | Nocardia_coeliaca |
| Otu00644 | Bacteroidetes | Sphingobacteriia | Sphingobacteriales | Saprospiraceae | Portibacter | Portibacter_unclassified |
| Otu00645 | Firmicutes | Clostridia | Clostridiales | Peptostreptococcaceae | Clostridium XI | Clostridium_hiranonis |
| Otu00648 | Proteobacteria | Betaproteobacteria | Burkholderiales | Comamonadaceae | Limnohabitans | Limnohabitans_planktonicus |
| Otu00651 | Actinobacteria | Actinobacteria | Actinomycetales | Micrococcaceae | Arthrobacter | Arthrobacter_sulfureus |
| Otu00653 | Proteobacteria | Betaproteobacteria | Burkholderiales | Alcaligenaceae | Parapusillimonas | Parapusillimonas_unclassified |
| Otu00654 | Bacteroidetes | Sphingobacteriia | Sphingobacteriales | Sphingobacteriaceae | Mucilaginibacter | Mucilaginibacter_rigui |
| Otu00656 | Bacteroidetes | Flavobacteriia | Flavobacteriales | Flavobacteriaceae | Flavobacterium | Flavobacterium_unclassified |
| Otu00655 | Proteobacteria | Alphaproteobacteria | Rhodospirillales | Reyranella | Reyranella | Reyranella_massiliensis |
| Otu00660 | Proteobacteria | Alphaproteobacteria | Rhizobiales | Hyphomicrobiaceae | Devosia | Devosia_limi |
| Otu00664 | Proteobacteria | Alphaproteobacteria | Rhizobiales | Rhizobiaceae | Rhizobium | Rhizobium_vitis |
| Otu00663 | Proteobacteria | Alphaproteobacteria | Sphingomonadales | Sphingomonadaceae | Sphingomonas | Sphingomonas_oligophenolica |
| Otu00665 | Proteobacteria | Gammaproteobacteria | Pseudomonadales | Pseudomonadaceae | Rhizobacter | Rhizobacter_unclassified |
| Otu00670 | Proteobacteria | Betaproteobacteria | Burkholderiales | Oxalobacteraceae | Duganella | Duganella_zoogloeoides |
| Otu00677 | Actinobacteria | Actinobacteria | Actinomycetales | Corynebacteriaceae | Corynebacterium | Corynebacterium_unclassified |
| Otu00678 | Bacteroidetes | Flavobacteriia | Flavobacteriales | Flavobacteriaceae | Vitellibacter | Vitellibacter_unclassified |
| Otu00679 | Bacteroidetes | Sphingobacteriia | Sphingobacteriales | Sphingobacteriaceae | Pedobacter | Pedobacter_steynii |
| Otu00681 | Proteobacteria | Gammaproteobacteria | Pseudomonadales | Moraxellaceae | Psychrobacter | Psychrobacter_fulvigenes |
| Otu00688 | Bacteroidetes | Flavobacteriia | Flavobacteriales | Flavobacteriaceae | Flavobacterium | Flavobacterium_noncentrifugens |
| Otu00691 | Bacteroidetes | Flavobacteriia | Flavobacteriales | Flavobacteriaceae | Elizabethkingia | Elizabethkingia_miricola |
| Otu00692 | Proteobacteria | Gammaproteobacteria | Pseudomonadales | Moraxellaceae | Acinetobacter | Acinetobacter_baumannii |
| Otu00695 | Actinobacteria | Actinobacteria | Solirubrobacterales | Patulibacteraceae | Patulibacter | Patulibacter_unclassified |
| Otu00700 | Firmicutes | Bacilli | Lactobacillales | Streptococcaceae | Streptococcus | Streptococcus_alactolyticus |
| Otu00702 | Actinobacteria | Actinobacteria | Actinomycetales | Microbacteriaceae | Microbacterium | Microbacterium_arthrosphaerae |
| Otu00705 | Proteobacteria | Betaproteobacteria | Burkholderiales | Comamonadaceae | Acidovorax | Acidovorax_defluvii |
| Otu00708 | Firmicutes | Bacilli | Lactobacillales | Carnobacteriaceae | Allofustis | Allofustis_unclassified |
| Otu00711 | Proteobacteria | Alphaproteobacteria | Rhodobacterales | Rhodobacteraceae | Pseudorhodobacter | Pseudorhodobacter_collinsensis |
| Otu00713 | Proteobacteria | Betaproteobacteria | Burkholderiales | Comamonadaceae | Rhodoferax | Rhodoferax_unclassified |
| Otu00717 | Bacteroidetes | Sphingobacteriia | Sphingobacteriales | Saprospiraceae | Haliscomenobacter | Haliscomenobacter_unclassified |
| Otu00722 | Bacteroidetes | Flavobacteriia | Flavobacteriales | Flavobacteriaceae | Flavobacterium | Flavobacterium_unclassified |
| Otu00723 | Bacteroidetes | Cytophagia | Cytophagales | Cytophagaceae | Dyadobacter | Dyadobacter_hamtensis |
| Otu00725 | Bacteroidetes | Flavobacteriia | Flavobacteriales | Flavobacteriaceae | Flavobacterium | Flavobacterium_branchiarum |
| Otu00726 | Firmicutes | Bacilli | Lactobacillales | Carnobacteriaceae | Carnobacterium | Carnobacterium_viridans |
| Otu00727 | Firmicutes | Negativicutes | Selenomonadales | Veillonellaceae | Megasphaera | Megasphaera_indica |
| Otu00728 | Bacteroidetes | Bacteroidia | Bacteroidales | Porphyromonadaceae | Petrimonas | Petrimonas_unclassified |
| Otu00730 | Bacteroidetes | Flavobacteriia | Flavobacteriales | Flavobacteriaceae | Flavobacterium | Flavobacterium_segetis |
| Otu00733 | Proteobacteria | Alphaproteobacteria | Rhizobiales | Phyllobacteriaceae | Hoeflea | Hoeflea_alexandrii |
| Otu00734 | Bacteroidetes | Sphingobacteriia | Sphingobacteriales | Sphingobacteriaceae | Mucilaginibacter | Mucilaginibacter_soyangensis |
| Otu00737 | Bacteroidetes | Flavobacteriia | Flavobacteriales | Flavobacteriaceae | Chryseobacterium | Chryseobacterium_rigui |
| Otu00738 | Proteobacteria | Alphaproteobacteria | Sphingomonadales | Sphingomonadaceae | Sphingomonas | Sphingomonas_aerolata |
| Otu00740 | Bacteroidetes | Sphingobacteriia | Sphingobacteriales | Sphingobacteriaceae | Pedobacter | Pedobacter_duraquae |
| Otu00743 | Proteobacteria | Alphaproteobacteria | Rhizobiales | Hyphomicrobiaceae | Devosia | Devosia_chinhatensis |
| Otu00748 | Bacteroidetes | Flavobacteriia | Flavobacteriales | Flavobacteriaceae | Flavobacterium | Flavobacterium_sinopsychrotolerans |
| Otu00752 | Actinobacteria | Actinobacteria | Actinomycetales | Microbacteriaceae | Microbacterium | Microbacterium_unclassified |
| Otu00754 | Proteobacteria | Gammaproteobacteria | Pseudomonadales | Pseudomonadaceae | Pseudomonas | Pseudomonas_taiwanensis |
| Otu00756 | Proteobacteria | Alphaproteobacteria | Sphingomonadales | Sphingomonadaceae | Sphingomonas | Sphingomonas_faeni |
| Otu00757 | Proteobacteria | Betaproteobacteria | Burkholderiales | Comamonadaceae | Simplicispira | Simplicispira_unclassified |
| Otu00765 | Firmicutes | Bacilli | Lactobacillales | Carnobacteriaceae | Carnobacterium | Carnobacterium_gallinarum |
| Otu00766 | Bacteroidetes | Flavobacteriia | Flavobacteriales | Flavobacteriaceae | Flavobacterium | Flavobacterium_algicola |
| Otu00767 | Proteobacteria | Betaproteobacteria | Burkholderiales | Comamonadaceae | Polaromonas | Polaromonas_naphthalenivorans |
| Otu00771 | Bacteroidetes | Cytophagia | Cytophagales | Cytophagaceae | Dyadobacter | Dyadobacter_unclassified |
| Otu00775 | Bacteroidetes | Sphingobacteriia | Sphingobacteriales | Saprospiraceae | Portibacter | Portibacter_unclassified |
| Otu00776 | Firmicutes | Clostridia | Clostridiales | Lachnospiraceae | Clostridium XlVa | Clostridium XlVa_unclassified |
| Otu00777 | Proteobacteria | Betaproteobacteria | Burkholderiales | Alcaligenaceae | Parapusillimonas | Parapusillimonas_unclassified |
| Otu00778 | Proteobacteria | Alphaproteobacteria | Rhizobiales | Phyllobacteriaceae | Mesorhizobium | Mesorhizobium_amorphae |
| Otu00779 | Firmicutes | Erysipelotrichia | Erysipelotrichales | Erysipelotrichaceae | Clostridium XVIII | Clostridium_spiroforme |
| Otu00782 | Actinobacteria | Actinobacteria | Actinomycetales | Microbacteriaceae | Salinibacterium | Salinibacterium_unclassified |
| Otu00786 | Candidatus Saccharibacteria | Saccharibacteria_genera_incertae_sedis | Saccharibacteria_genera_incertae_sedis | Saccharibacteria_genera_incertae_sedis | Saccharibacteria_genera_incertae_sedis | Saccharibacteria_genera_incertae_sedis_unclassified |
| Otu00790 | Bacteroidetes | Flavobacteriia | Flavobacteriales | Flavobacteriaceae | Flavobacterium | Flavobacterium_algicola |
| Otu00791 | Bacteroidetes | Sphingobacteriia | Sphingobacteriales | Chitinophagaceae | Niastella | Niastella_unclassified |
| Otu00794 | Bacteroidetes | Sphingobacteriia | Sphingobacteriales | Sphingobacteriaceae | Pedobacter | Pedobacter_alluvionis |
| Otu00795 | Firmicutes | Bacilli | Lactobacillales | Carnobacteriaceae | Atopostipes | Atopostipes_unclassified |
| Otu00799 | Firmicutes | Clostridia | Clostridiales | Peptostreptococcaceae | Romboutsia | Romboutsia_unclassified |
| Otu00801 | Bacteroidetes | Flavobacteriia | Flavobacteriales | Flavobacteriaceae | Chryseobacterium | Chryseobacterium_chaponense |
| Otu00800 | Bacteroidetes | Flavobacteriia | Flavobacteriales | Flavobacteriaceae | Flavobacterium | Flavobacterium_unclassified |
| Otu00803 | Bacteroidetes | Sphingobacteriia | Sphingobacteriales | Sphingobacteriaceae | Pedobacter | Pedobacter_alluvionis |
| Otu00805 | Actinobacteria | Actinobacteria | Actinomycetales | Nocardioidaceae | Aeromicrobium | Aeromicrobium_fastidiosum |
| Otu00809 | Bacteroidetes | Sphingobacteriia | Sphingobacteriales | Sphingobacteriaceae | Pedobacter | Pedobacter_unclassified |
| Otu00811 | Bacteroidetes | Flavobacteriia | Flavobacteriales | Flavobacteriaceae | Flavobacterium | Flavobacterium_rivuli |
| Otu00816 | Bacteroidetes | Flavobacteriia | Flavobacteriales | Flavobacteriaceae | Flavobacterium | Flavobacterium_phocarum |
| Otu00817 | Proteobacteria | Alphaproteobacteria | Rhizobiales | Rhizobiaceae | Rhizobium | Rhizobium_taibaishanense |
| Otu00826 | Bacteroidetes | Flavobacteriia | Flavobacteriales | Flavobacteriaceae | Flavobacterium | Flavobacterium_unclassified |
| Otu00828 | Firmicutes | Bacilli | Lactobacillales | Aerococcaceae | Aerococcus | Aerococcus_urinaeequi |
| Otu00829 | Proteobacteria | Betaproteobacteria | Neisseriales | Neisseriaceae | Rivicola | Rivicola_unclassified |
| Otu00833 | Firmicutes | Bacilli | Bacillales | Planococcaceae | Planomicrobium | Planomicrobium_unclassified |
| Otu00835 | Firmicutes | Clostridia | Clostridiales | Lachnospiraceae | Clostridium XlVb | Clostridium XlVb_unclassified |
| Otu00836 | Proteobacteria | Betaproteobacteria | Burkholderiales | Comamonadaceae | Malikia | Malikia_spinosa |
| Otu00837 | Proteobacteria | Alphaproteobacteria | Rhodobacterales | Rhodobacteraceae | Pseudorhodobacter | Pseudorhodobacter_aquimaris |
| Otu00839 | Proteobacteria | Betaproteobacteria | Burkholderiales | Alcaligenaceae | Paenalcaligenes | Paenalcaligenes_unclassified |
| Otu00842 | Bacteroidetes | Sphingobacteriia | Sphingobacteriales | Sphingobacteriaceae | Pedobacter | Pedobacter_petrophilus |
| Otu00845 | Proteobacteria | Betaproteobacteria | Neisseriales | Neisseriaceae | Deefgea | Deefgea_rivuli |
| Otu00847 | Bacteroidetes | Sphingobacteriia | Sphingobacteriales | Sphingobacteriaceae | Sphingobacterium | Sphingobacterium_unclassified |
| Otu00849 | Proteobacteria | Betaproteobacteria | Burkholderiales | Comamonadaceae | Polaromonas | Polaromonas_unclassified |
| Otu00856 | Firmicutes | Clostridia | Clostridiales | Lachnospiraceae | Blautia | Blautia_luti |
| Otu00859 | Proteobacteria | Alphaproteobacteria | Rhizobiales | Rhizobiaceae | Rhizobium | Rhizobium_skierniewicense |
| Otu00860 | Proteobacteria | Gammaproteobacteria | Thiotrichales | Piscirickettsiaceae | Galenea | Galenea_unclassified |
| Otu00869 | Proteobacteria | Betaproteobacteria | Burkholderiales | Oxalobacteraceae | Undibacterium | Undibacterium_seohonense |
| Otu00872 | Proteobacteria | Deltaproteobacteria | Myxococcales | Polyangiaceae | Jahnella | Jahnella_unclassified |
| Otu00875 | Proteobacteria | Alphaproteobacteria | Sphingomonadales | Sphingomonadaceae | Hephaestia | Hephaestia_caeni |
| Otu00879 | Bacteroidetes | Flavobacteriia | Flavobacteriales | Flavobacteriaceae | Flavobacterium | Flavobacterium_unclassified |
| Otu00877 | Proteobacteria | Alphaproteobacteria | Sphingomonadales | Sphingomonadaceae | Novosphingobium | Novosphingobium_fluoreni |
| Otu00882 | Bacteroidetes | Flavobacteriia | Flavobacteriales | Flavobacteriaceae | Flavobacterium | Flavobacterium_unclassified |
| Otu00887 | Proteobacteria | Alphaproteobacteria | Caulobacterales | Caulobacteraceae | Asticcacaulis | Asticcacaulis_benevestitus |
| Otu00886 | Bacteroidetes | Flavobacteriia | Flavobacteriales | Flavobacteriaceae | Flavobacterium | Flavobacterium_psychrolimnae |
| Otu00888 | Actinobacteria | Actinobacteria | Actinomycetales | Microbacteriaceae | Agreia | Agreia_unclassified |
| Otu00895 | Proteobacteria | Alphaproteobacteria | Caulobacterales | Caulobacteraceae | Brevundimonas | Brevundimonas_mediterranea |
| Otu00896 | Proteobacteria | Betaproteobacteria | Rhodocyclales | Rhodocyclaceae | Zoogloea | Zoogloea_unclassified |
| Otu00897 | Actinobacteria | Actinobacteria | Actinomycetales | Microbacteriaceae | Clavibacter | Clavibacter_michiganensis |
| Otu00900 | Proteobacteria | Betaproteobacteria | Rhodocyclales | Rhodocyclaceae | Georgfuchsia | Georgfuchsia_unclassified |
| Otu00902 | Proteobacteria | Gammaproteobacteria | Pseudomonadales | Pseudomonadaceae | Pseudomonas | Pseudomonas_punonensis |
| Otu00918 | Proteobacteria | Betaproteobacteria | Burkholderiales | Burkholderiales_incertae_sedis | Methylibium | Methylibium_petroleiphilum |
| Otu00920 | Bacteroidetes | Flavobacteriia | Flavobacteriales | Flavobacteriaceae | Chryseobacterium | Chryseobacterium_vietnamense |
| Otu00922 | Candidatus Saccharibacteria | Saccharibacteria_genera_incertae_sedis | Saccharibacteria_genera_incertae_sedis | Saccharibacteria_genera_incertae_sedis | Saccharibacteria_genera_incertae_sedis | Saccharibacteria_genera_incertae_sedis_unclassified |
| Otu00921 | Firmicutes | Clostridia | Thermoanaerobacterales | Thermoanaerobacteraceae | Thermanaeromonas | Thermanaeromonas_unclassified |
| Otu00928 | Firmicutes | Bacilli | Lactobacillales | Carnobacteriaceae | Carnobacterium | Carnobacterium_inhibens |
| Otu00931 | Bacteroidetes | Sphingobacteriia | Sphingobacteriales | Saprospiraceae | Portibacter | Portibacter_unclassified |
| Otu00933 | Actinobacteria | Actinobacteria | Actinomycetales | Propionibacteriaceae | Propioniciclava | Propioniciclava_unclassified |
| Otu00936 | Bacteroidetes | Sphingobacteriia | Sphingobacteriales | Sphingobacteriaceae | Mucilaginibacter | Mucilaginibacter_polytrichastri |
| Otu00940 | Proteobacteria | Betaproteobacteria | Burkholderiales | Comamonadaceae | Xylophilus | Xylophilus_ampelinus |
| Otu00943 | Proteobacteria | Betaproteobacteria | Burkholderiales | Comamonadaceae | Caenimonas | Caenimonas_koreensis |
| Otu00941 | Proteobacteria | Betaproteobacteria | Burkholderiales | Comamonadaceae | Polaromonas | Polaromonas_jejuensis |
| Otu00945 | Proteobacteria | Deltaproteobacteria | Myxococcales | Polyangiaceae | Sorangium | Sorangium_unclassified |
| Otu00951 | Firmicutes | Bacilli | Lactobacillales | Aerococcaceae | Facklamia | Facklamia_unclassified |
| Otu00954 | Proteobacteria | Gammaproteobacteria | Pseudomonadales | Moraxellaceae | Acinetobacter | Acinetobacter_guangdongensis |
| Otu00952 | Actinobacteria | Actinobacteria | Acidimicrobiales | Iamiaceae | Aquihabitans | Aquihabitans_daechungensis |
| Otu00956 | Proteobacteria | Gammaproteobacteria | Pseudomonadales | Pseudomonadaceae | Pseudomonas | Pseudomonas_litoralis |
| Otu00958 | Firmicutes | Erysipelotrichia | Erysipelotrichales | Erysipelotrichaceae | Clostridium XVIII | Clostridium XVIII_unclassified |
| Otu00957 | Bacteroidetes | Flavobacteriia | Flavobacteriales | Flavobacteriaceae | Flavobacterium | Flavobacterium_unclassified |
| Otu00961 | Actinobacteria | Actinobacteria | Actinomycetales | Micrococcaceae | Arthrobacter | Arthrobacter_humicola |
| Otu00960 | Firmicutes | Clostridia | Clostridiales | Ruminococcaceae | Oscillibacter | Oscillibacter_unclassified |
| Otu00963 | Proteobacteria | Betaproteobacteria | Burkholderiales | Comamonadaceae | Polaromonas | Polaromonas_unclassified |
| Otu00965 | Proteobacteria | Alphaproteobacteria | Rhizobiales | Methylobacteriaceae | Methylobacterium | Methylobacterium_adhaesivum |
| Otu00966 | Bacteroidetes | Sphingobacteriia | Sphingobacteriales | Sphingobacteriaceae | Pseudosphingobacterium | Pseudosphingobacterium_unclassified |
| Otu00967 | Proteobacteria | Alphaproteobacteria | Sphingomonadales | Sphingomonadaceae | Sphingobium | Sphingobium_baderi |
| Otu00971 | Proteobacteria | Gammaproteobacteria | Pseudomonadales | Moraxellaceae | Acinetobacter | Acinetobacter_johnsonii |
| Otu00973 | Actinobacteria | Actinobacteria | Actinomycetales | Micromonosporaceae | Actinoplanes | Actinoplanes_nipponensis |
| Otu00978 | Firmicutes | Clostridia | Clostridiales | Lachnospiraceae | Blautia | Blautia_stercoris |
| Otu00980 | Proteobacteria | Alphaproteobacteria | Rhizobiales | Hyphomicrobiaceae | Devosia | Devosia_glacialis |
| Otu00983 | Proteobacteria | Gammaproteobacteria | Pseudomonadales | Pseudomonadaceae | Pseudomonas | Pseudomonas_bauzanensis |
| Otu00986 | Proteobacteria | Betaproteobacteria | Burkholderiales | Alcaligenaceae | Oligella | Oligella_ureolytica |
| Otu00990 | Bacteroidetes | Cytophagia | Cytophagales | Chryseolinea | Chryseolinea | Chryseolinea_unclassified |
| Otu00996 | Proteobacteria | Betaproteobacteria | Burkholderiales | Comamonadaceae | Giesbergeria | Giesbergeria_kuznetsovii |
| Otu00997 | Proteobacteria | Alphaproteobacteria | Rhizobiales | Brucellaceae | Ochrobactrum | Ochrobactrum_unclassified |
| Otu01000 | Proteobacteria | Gammaproteobacteria | Pseudomonadales | Moraxellaceae | Psychrobacter | Psychrobacter_lutiphocae |
| Otu00998 | Proteobacteria | Betaproteobacteria | Burkholderiales | Oxalobacteraceae | Undibacterium | Undibacterium_unclassified |
| Otu01002 | Bacteroidetes | Sphingobacteriia | Sphingobacteriales | Chitinophagaceae | Ferruginibacter | Ferruginibacter_lapsinanis |
| Otu01001 | Bacteroidetes | Flavobacteriia | Flavobacteriales | Flavobacteriaceae | Flavobacterium | Flavobacterium_fryxellicola |
| Otu01007 | Proteobacteria | Alphaproteobacteria | Rhizobiales | Xanthobacteraceae | Pseudolabrys | Pseudolabrys_unclassified |
| Otu01006 | Proteobacteria | Gammaproteobacteria | Xanthomonadales | Xanthomonadaceae | Thermomonas | Thermomonas_brevis |
| Otu01014 | Proteobacteria | Betaproteobacteria | Burkholderiales | Comamonadaceae | Comamonas | Comamonas_guangdongensis |
| Otu01018 | Actinobacteria | Actinobacteria | Actinomycetales | Microbacteriaceae | Plantibacter | Plantibacter_flavus |
| Otu01017 | Proteobacteria | Gammaproteobacteria | Pseudomonadales | Pseudomonadaceae | Serpens | Serpens_unclassified |
| Otu01023 | Bacteroidetes | Bacteroidia | Bacteroidales | Porphyromonadaceae | Butyricimonas | Butyricimonas_virosa |
| Otu01022 | Firmicutes | Bacilli | Bacillales | Planococcaceae | Planomicrobium | Planomicrobium_unclassified |
| Otu01027 | Bacteroidetes | Flavobacteriia | Flavobacteriales | Flavobacteriaceae | Flavobacterium | Flavobacterium_limicola |
| Otu01026 | Actinobacteria | Actinobacteria | Solirubrobacterales | Solirubrobacteraceae | Solirubrobacter | Solirubrobacter_unclassified |
| Otu01028 | Firmicutes | Negativicutes | Selenomonadales | Veillonellaceae | Pelosinus | Pelosinus_unclassified |
| Otu01030 | Actinobacteria | Actinobacteria | Actinomycetales | Microbacteriaceae | Lysinimonas | Lysinimonas_kribbensis |
| Otu01033 | Proteobacteria | Alphaproteobacteria | Sphingomonadales | Sphingomonadaceae | Novosphingobium | Novosphingobium_resinovorum |
| Otu01036 | Firmicutes | Bacilli | Bacillales | Planococcaceae | Sporosarcina | Sporosarcina_unclassified |
| Otu01038 | Bacteroidetes | Flavobacteriia | Flavobacteriales | Flavobacteriaceae | Chryseobacterium | Chryseobacterium_aahli |
| Otu01045 | Proteobacteria | Alphaproteobacteria | Sphingomonadales | Sphingomonadaceae | Sphingobium | Sphingobium_yanoikuyae |
| Otu01047 | Bacteroidetes | Flavobacteriia | Flavobacteriales | Flavobacteriaceae | Flavobacterium | Flavobacterium_branchiarum |
| Otu01049 | Proteobacteria | Betaproteobacteria | Burkholderiales | Oxalobacteraceae | Janthinobacterium | Janthinobacterium_svalbardensis |
| Otu01050 | Bacteroidetes | Flavobacteriia | Flavobacteriales | Flavobacteriaceae | Flavobacterium | Flavobacterium_unclassified |
| Otu01057 | Bacteroidetes | Flavobacteriia | Flavobacteriales | Flavobacteriaceae | Arenitalea | Arenitalea_unclassified |
| Otu01060 | Firmicutes | Clostridia | Clostridiales | Clostridiaceae 1 | Clostridium sensu stricto | Clostridium sensu stricto_unclassified |
| Otu01052 | Chloroflexi | Anaerolineae | Anaerolineales | Anaerolineaceae | Levilinea | Levilinea_unclassified |
| Otu01055 | Actinobacteria | Actinobacteria | Actinomycetales | Microbacteriaceae | Microbacterium | Microbacterium_hominis |
| Otu01063 | Bacteroidetes | Bacteroidia | Bacteroidales | Rikenellaceae | Mucinivorans | Mucinivorans_unclassified |
| Otu01067 | Bacteroidetes | Flavobacteriia | Flavobacteriales | Flavobacteriaceae | Flavobacterium | Flavobacterium_hercynium |
| Otu01069 | Firmicutes | Clostridia | Clostridiales | Ruminococcaceae | Oscillibacter | Oscillibacter_unclassified |
| Otu01072 | Bacteroidetes | Flavobacteriia | Flavobacteriales | Flavobacteriaceae | Flavobacterium | Flavobacterium_terrigena |
| Otu01071 | Proteobacteria | Betaproteobacteria | Burkholderiales | Oxalobacteraceae | Janthinobacterium | Janthinobacterium_unclassified |
| Otu01074 | Proteobacteria | Alphaproteobacteria | Sphingomonadales | Sphingomonadaceae | Polymorphobacter | Polymorphobacter_multimanifer |
| Otu01077 | Proteobacteria | Alphaproteobacteria | Rhizobiales | Hyphomicrobiaceae | Devosia | Devosia_soli |
| Otu01078 | Firmicutes | Clostridia | Clostridiales | Lachnospiraceae | Lachnospiracea_incertae_sedis | Lachnospiracea_incertae_sedis_unclassified |
| Otu01081 | Firmicutes | Clostridia | Clostridiales | Ruminococcaceae | Faecalibacterium | Faecalibacterium_prausnitzii |
| Otu01083 | Proteobacteria | Alphaproteobacteria | Sphingomonadales | Sphingomonadaceae | Sphingomonas | Sphingomonas_echinoides |
| Otu01086 | Proteobacteria | Betaproteobacteria | Burkholderiales | Comamonadaceae | Rhodoferax | Rhodoferax_unclassified |
| Otu01089 | Firmicutes | Bacilli | Bacillales | Planococcaceae | Sporosarcina | Sporosarcina_siberiensis |
| Otu01090 | Bacteroidetes | Flavobacteriia | Flavobacteriales | Flavobacteriaceae | Flavobacterium | Flavobacterium_araucananum |
| Otu01094 | Proteobacteria | Gammaproteobacteria | Pseudomonadales | Moraxellaceae | Alkanindiges | Alkanindiges_unclassified |
| Otu01097 | Firmicutes | Clostridia | Clostridiales | Clostridiaceae 1 | Clostridium sensu stricto | Clostridium sensu stricto_unclassified |
| Otu01096 | Proteobacteria | Alphaproteobacteria | Sphingomonadales | Sphingomonadaceae | Sphingomonas | Sphingomonas_desiccabilis |
| Otu01098 | Actinobacteria | Actinobacteria | Actinomycetales | Microbacteriaceae | Leucobacter | Leucobacter_exalbidus |
| Otu01099 | Proteobacteria | Betaproteobacteria | Burkholderiales | Oxalobacteraceae | Undibacterium | Undibacterium_unclassified |
| Otu01103 | Proteobacteria | Betaproteobacteria | Burkholderiales | Oxalobacteraceae | Undibacterium | Undibacterium_jejuense |
| Otu01108 | Proteobacteria | Epsilonproteobacteria | Campylobacterales | Campylobacteraceae | Arcobacter | Arcobacter_defluvii |
| Otu01109 | Proteobacteria | Alphaproteobacteria | Caulobacterales | Caulobacteraceae | Caulobacter | Caulobacter_fusiformis |
| Otu01104 | Actinobacteria | Actinobacteria | Actinomycetales | Microbacteriaceae | Salinibacterium | Salinibacterium_unclassified |
| Otu01113 | Proteobacteria | Alphaproteobacteria | Rhodobacterales | Rhodobacteraceae | Paracoccus | Paracoccus_marinus |
| Otu01115 | Proteobacteria | Betaproteobacteria | Burkholderiales | Oxalobacteraceae | Undibacterium | Undibacterium_unclassified |
| Otu01117 | Proteobacteria | Gammaproteobacteria | Pseudomonadales | Pseudomonadaceae | Cellvibrio | Cellvibrio_gandavensis |
| Otu01121 | Bacteroidetes | Sphingobacteriia | Sphingobacteriales | Chitinophagaceae | Chitinophaga | Chitinophaga_oryziterrae |
| Otu01122 | Proteobacteria | Betaproteobacteria | Burkholderiales | Oxalobacteraceae | Duganella | Duganella_zoogloeoides |
| Otu01125 | Bacteroidetes | Cytophagia | Cytophagales | Cytophagaceae | Arcicella | Arcicella_unclassified |
| Otu01124 | Bacteroidetes | Flavobacteriia | Flavobacteriales | Flavobacteriaceae | Flavobacterium | Flavobacterium_resistens |
| Otu01129 | Proteobacteria | Gammaproteobacteria | Pseudomonadales | Pseudomonadaceae | Cellvibrio | Cellvibrio_diazotrophicus |
| Otu01128 | Proteobacteria | Betaproteobacteria | Rhodocyclales | Rhodocyclaceae | Sulfurisoma | Sulfurisoma_unclassified |
| Otu01133 | Proteobacteria | Alphaproteobacteria | Rhodospirillales | Rhodospirillaceae | Dongia | Dongia_unclassified |
| Otu01132 | Actinobacteria | Actinobacteria | Actinomycetales | Microbacteriaceae | Frigoribacterium | Frigoribacterium_unclassified |
| Otu01131 | Firmicutes | Bacilli | Bacillales | Planococcaceae | Planomicrobium | Planomicrobium_unclassified |
| Otu01134 | Bacteroidetes | Cytophagia | Cytophagales | Cytophagaceae | Pseudarcicella | Pseudarcicella_unclassified |
| Otu01130 | Bacteroidetes | Flavobacteriia | Flavobacteriales | Flavobacteriaceae | Zhouia | Zhouia_unclassified |
| Otu01136 | Proteobacteria | Gammaproteobacteria | Legionellales | Coxiellaceae | Diplorickettsia | Diplorickettsia_unclassified |
| Otu01137 | Actinobacteria | Actinobacteria | Actinomycetales | Micromonosporaceae | Micromonospora | Micromonospora_olivasterospora |
| Otu01143 | Firmicutes | Bacilli | Lactobacillales | Aerococcaceae | Facklamia | Facklamia_unclassified |
| Otu01149 | Firmicutes | Erysipelotrichia | Erysipelotrichales | Erysipelotrichaceae | Coprobacillus | Coprobacillus_unclassified |
| Otu01148 | Firmicutes | Erysipelotrichia | Erysipelotrichales | Erysipelotrichaceae | Faecalicoccus | Faecalicoccus_unclassified |
| Otu01152 | Proteobacteria | Deltaproteobacteria | Bdellovibrionales | Bacteriovoracaceae | Bacteriovorax | Bacteriovorax_unclassified |
| Otu01151 | Acidobacteria | Acidobacteria_Gp4 | Gp4 | Gp4 | Gp4 | Gp4_unclassified |
| Otu01157 | Bacteroidetes | Sphingobacteriia | Sphingobacteriales | Sphingobacteriaceae | Mucilaginibacter | Mucilaginibacter_unclassified |
| Otu01154 | Proteobacteria | Deltaproteobacteria | Myxococcales | Polyangiaceae | Sorangium | Sorangium_unclassified |
| Otu01161 | Bacteroidetes | Flavobacteriia | Flavobacteriales | Flavobacteriaceae | Flavobacterium | Flavobacterium_unclassified |
| Otu01162 | Actinobacteria | Actinobacteria | Actinomycetales | Kineosporiaceae | Kineococcus | Kineococcus_unclassified |
| Otu01159 | Proteobacteria | Betaproteobacteria | Rhodocyclales | Rhodocyclaceae | Thauera | Thauera_terpenica |
| Otu01163 | Candidatus Saccharibacteria | Saccharibacteria_genera_incertae_sedis | Saccharibacteria_genera_incertae_sedis | Saccharibacteria_genera_incertae_sedis | Saccharibacteria_genera_incertae_sedis | Saccharibacteria_genera_incertae_sedis_unclassified |
| Otu01166 | Actinobacteria | Actinobacteria | Actinomycetales | Nocardioidaceae | Marmoricola | Marmoricola_aequoreus |
| Otu01169 | Firmicutes | Bacilli | Lactobacillales | Streptococcaceae | Lactococcus | Lactococcus_lactis |
| Otu01181 | Actinobacteria | Actinobacteria | Actinomycetales | Microbacteriaceae | Frigoribacterium | Frigoribacterium_unclassified |
| Otu01179 | Firmicutes | Bacilli | Bacillales | Planococcaceae | Sporosarcina | Sporosarcina_unclassified |
| Otu01184 | Proteobacteria | Gammaproteobacteria | Aeromonadales | Aeromonadaceae | Aeromonas | Aeromonas_dhakensis |
| Otu01185 | Bacteroidetes | Flavobacteriia | Flavobacteriales | Flavobacteriaceae | Flavobacterium | Flavobacterium_myungsuense |
| Otu01183 | Bacteroidetes | Flavobacteriia | Flavobacteriales | Flavobacteriaceae | Flavobacterium | Flavobacterium_unclassified |
| Otu01189 | Firmicutes | Bacilli | Lactobacillales | Carnobacteriaceae | Atopostipes | Atopostipes_unclassified |
| Otu01191 | Bacteroidetes | Flavobacteriia | Flavobacteriales | Flavobacteriaceae | Flavobacterium | Flavobacterium_luteum |
| Otu01193 | Proteobacteria | Deltaproteobacteria | Myxococcales | Labilitrichaceae | Labilithrix | Labilithrix_unclassified |
| Otu01203 | Proteobacteria | Betaproteobacteria | Burkholderiales | Burkholderiales_incertae_sedis | Aquabacterium | Aquabacterium_citratiphilum |
| Otu01201 | Firmicutes | Bacilli | Lactobacillales | Lactobacillaceae | Lactobacillus | Lactobacillus_unclassified |
| Otu01207 | Actinobacteria | Actinobacteria | Actinomycetales | Demequinaceae | Demequina | Demequina_aurantiaca |
| Otu01204 | Actinobacteria | Actinobacteria | Actinomycetales | Microbacteriaceae | Herbiconiux | Herbiconiux_unclassified |
| Otu01213 | Firmicutes | Bacilli | Lactobacillales | Enterococcaceae | Enterococcus | Enterococcus_cecorum |
| Otu01215 | Proteobacteria | Alphaproteobacteria | Sphingomonadales | Sphingomonadaceae | Novosphingobium | Novosphingobium_unclassified |
| Otu01218 | Firmicutes | Bacilli | Bacillales | Staphylococcaceae | Staphylococcus | Staphylococcus_argenteus |
| Otu01216 | Firmicutes | Bacilli | Lactobacillales | Carnobacteriaceae | Trichococcus | Trichococcus_palustris |
| Otu01221 | Bacteroidetes | Flavobacteriia | Flavobacteriales | Flavobacteriaceae | Flavobacterium | Flavobacterium_keumense |
| Otu01219 | Acidobacteria | Acidobacteria_Gp6 | Gp6 | Gp6 | Gp6 | Gp6_unclassified |
| Otu01222 | Proteobacteria | Alphaproteobacteria | Rhodospirillales | Acetobacteraceae | Rhodopila | Rhodopila_unclassified |
| Otu01226 | Proteobacteria | Epsilonproteobacteria | Campylobacterales | Helicobacteraceae | Sulfurimonas | Sulfurimonas_unclassified |
| Otu01228 | Bacteroidetes | Bacteroidia | Bacteroidales | Prevotellaceae | Prevotella | Prevotella_stercorea |
| Otu01232 | Actinobacteria | Actinobacteria | Actinomycetales | Intrasporangiaceae | Lapillicoccus | Lapillicoccus_unclassified |
| Otu01236 | Proteobacteria | Alphaproteobacteria | Sphingomonadales | Sphingomonadaceae | Sphingomonas | Sphingomonas_panacis |
| Otu01241 | Bacteroidetes | Flavobacteriia | Flavobacteriales | Flavobacteriaceae | Flavobacterium | Flavobacterium_unclassified |
| Otu01239 | Actinobacteria | Actinobacteria | Solirubrobacterales | Patulibacteraceae | Patulibacter | Patulibacter_unclassified |
| Otu01242 | Bacteroidetes | Sphingobacteriia | Sphingobacteriales | Saprospiraceae | Portibacter | Portibacter_unclassified |
| Otu01243 | Proteobacteria | Gammaproteobacteria | Pseudomonadales | Moraxellaceae | Acinetobacter | Acinetobacter_indicus |
| Otu01244 | Proteobacteria | Alphaproteobacteria | Sphingomonadales | Sphingomonadaceae | Sphingomonas | Sphingomonas_crusticola |
| Otu01248 | Firmicutes | Clostridia | Clostridiales | Clostridiaceae 1 | Clostridium sensu stricto | Clostridium_tagluense |
| Otu01247 | Bacteroidetes | Sphingobacteriia | Sphingobacteriales | Sphingobacteriaceae | Pedobacter | Pedobacter_aquatilis |
| Otu01249 | Bacteroidetes | Flavobacteriia | Flavobacteriales | Flavobacteriaceae | Flavobacterium | Flavobacterium_unclassified |
| Otu01250 | Proteobacteria | Alphaproteobacteria | Rhizobiales | Methylobacteriaceae | Methylobacterium | Methylobacterium_bullatum |
| Otu01252 | Bacteroidetes | Flavobacteriia | Flavobacteriales | Flavobacteriaceae | Mariniflexile | Mariniflexile_unclassified |
| Otu01256 | Proteobacteria | Betaproteobacteria | Burkholderiales | Burkholderiales_incertae_sedis | Piscinibacter | Piscinibacter_aquaticus |
| Otu01255 | Proteobacteria | Betaproteobacteria | Burkholderiales | Comamonadaceae | Polaromonas | Polaromonas_unclassified |
| Otu01258 | Proteobacteria | Betaproteobacteria | Burkholderiales | Comamonadaceae | Polaromonas | Polaromonas_unclassified |
| Otu01254 | Firmicutes | Bacilli | Lactobacillales | Leuconostocaceae | Weissella | Weissella_hellenica |
| Otu01260 | Proteobacteria | Alphaproteobacteria | Rhizobiales | Aurantimonadaceae | Aureimonas | Aureimonas_unclassified |
| Otu01266 | Firmicutes | Clostridia | Clostridiales | Lachnospiraceae | Clostridium XlVa | Clostridium_asparagiforme |
| Otu01265 | Bacteroidetes | Flavobacteriia | Flavobacteriales | Flavobacteriaceae | Flavobacterium | Flavobacterium_noncentrifugens |
| Otu01259 | Firmicutes | Bacilli | Lactobacillales | Leuconostocaceae | Leuconostoc | Leuconostoc_mesenteroides |
| Otu01268 | Firmicutes | Bacilli | Bacillales | Bacillaceae 1 | Anoxybacillus | Anoxybacillus_tepidamans |
| Otu01267 | Actinobacteria | Actinobacteria | Actinomycetales | Micrococcaceae | Arthrobacter | Arthrobacter_russicus |
| Otu01269 | Cyanobacteria/Chloroplast | Chloroplast | Chloroplast | Chloroplast | Cryptomonadaceae | Cryptomonadaceae_unclassified |
| Otu01271 | Bacteroidetes | Flavobacteriia | Flavobacteriales | Flavobacteriaceae | Flavobacterium | Flavobacterium_hercynium |
| Otu01281 | Bacteroidetes | Cytophagia | Cytophagales | Cytophagaceae | Arcicella | Arcicella_unclassified |
| Otu01272 | Proteobacteria | Betaproteobacteria | Burkholderiales | Comamonadaceae | Giesbergeria | Giesbergeria_unclassified |
| Otu01275 | Bacteroidetes | Sphingobacteriia | Sphingobacteriales | Sphingobacteriaceae | Mucilaginibacter | Mucilaginibacter_paludis |
| Otu01280 | Actinobacteria | Actinobacteria | Actinomycetales | Intrasporangiaceae | Oryzihumus | Oryzihumus_unclassified |
| Otu01274 | Proteobacteria | Betaproteobacteria | Burkholderiales | Comamonadaceae | Polaromonas | Polaromonas_unclassified |
| Otu01279 | Bacteroidetes | Flavobacteriia | Flavobacteriales | Cryomorphaceae | Salinirepens | Salinirepens_unclassified |
| Otu01276 | Proteobacteria | Gammaproteobacteria | Xanthomonadales | Xanthomonadaceae | Stenotrophomonas | Stenotrophomonas_rhizophila |
| Otu01282 | Proteobacteria | Gammaproteobacteria | Pseudomonadales | Moraxellaceae | Alkanindiges | Alkanindiges_unclassified |
| Otu01284 | Bacteroidetes | Flavobacteriia | Flavobacteriales | Flavobacteriaceae | Flavobacterium | Flavobacterium_unclassified |
| Otu01289 | Bacteroidetes | Sphingobacteriia | Sphingobacteriales | Chitinophagaceae | Taibaiella | Taibaiella_unclassified |
| Otu01294 | Proteobacteria | Gammaproteobacteria | Oceanospirillales | Oceanospirillaceae | Marinospirillum | Marinospirillum_unclassified |
| Otu01297 | Proteobacteria | Betaproteobacteria | Burkholderiales | Alcaligenaceae | Advenella | Advenella_alkanexedens |
| Otu01299 | Actinobacteria | Actinobacteria | Actinomycetales | Microbacteriaceae | Microbacterium | Microbacterium_ginsengisoli |
| Otu01296 | Bacteroidetes | Sphingobacteriia | Sphingobacteriales | Sphingobacteriaceae | Pedobacter | Pedobacter_agri |
| Otu01308 | Actinobacteria | Actinobacteria | Actinomycetales | Nocardioidaceae | Nocardioides | Nocardioides_unclassified |
| Otu01312 | Proteobacteria | Betaproteobacteria | Burkholderiales | Oxalobacteraceae | Collimonas | Collimonas_pratensis |
| Otu01315 | Proteobacteria | Alphaproteobacteria | Caulobacterales | Caulobacteraceae | Asticcacaulis | Asticcacaulis_biprosthecium |
| Otu01320 | Actinobacteria | Actinobacteria | Actinomycetales | Micrococcaceae | Arthrobacter | Arthrobacter_antarcticus |
| Otu01319 | Firmicutes | Erysipelotrichia | Erysipelotrichales | Erysipelotrichaceae | Holdemania | Holdemania_massiliensis |
| Otu01325 | Proteobacteria | Betaproteobacteria | Burkholderiales | Oxalobacteraceae | Janthinobacterium | Janthinobacterium_unclassified |
| Otu01323 | Proteobacteria | Alphaproteobacteria | Sphingomonadales | Sphingomonadaceae | Sphingorhabdus | Sphingorhabdus_arenilitoris |
| Otu01330 | Actinobacteria | Actinobacteria | Actinomycetales | Microbacteriaceae | Microbacterium | Microbacterium_liquefaciens |
| Otu01329 | Firmicutes | Clostridia | Clostridiales | Clostridiales_Incertae Sedis XI | Tissierella | Tissierella_unclassified |
| Otu01332 | Actinobacteria | Actinobacteria | Actinomycetales | Micromonosporaceae | Actinoplanes | Actinoplanes_nipponensis |
| Otu01335 | Bacteroidetes | Sphingobacteriia | Sphingobacteriales | Sphingobacteriaceae | Pedobacter | Pedobacter_westerhofensis |
| Otu01339 | Proteobacteria | Alphaproteobacteria | Rhizobiales | Bradyrhizobiaceae | Bosea | Bosea_massiliensis |
| Otu01348 | Bacteroidetes | Sphingobacteriia | Sphingobacteriales | Sphingobacteriaceae | Arcticibacter | Arcticibacter_unclassified |
| Otu01345 | Proteobacteria | Alphaproteobacteria | Sphingomonadales | Erythrobacteraceae | Erythrobacter | Erythrobacter_atlanticus |
| Otu01347 | Proteobacteria | Alphaproteobacteria | Rhizobiales | Phyllobacteriaceae | Phyllobacterium | Phyllobacterium_trifolii |
| Otu01346 | Proteobacteria | Alphaproteobacteria | Rhizobiales | Rhizobiaceae | Rhizobium | Rhizobium_cauense |
| Otu01353 | Bacteroidetes | Flavobacteriia | Flavobacteriales | Flavobacteriaceae | Flavobacterium | Flavobacterium_unclassified |
| Otu01354 | Bacteroidetes | Flavobacteriia | Flavobacteriales | Flavobacteriaceae | Flavobacterium | Flavobacterium_unclassified |
| Otu01355 | Proteobacteria | Gammaproteobacteria | Pseudomonadales | Pseudomonadaceae | Pseudomonas | Pseudomonas_deceptionensis |
| Otu01356 | Proteobacteria | Gammaproteobacteria | Pseudomonadales | Moraxellaceae | Psychrobacter | Psychrobacter_sanguinis |
| Otu01357 | Firmicutes | Clostridia | Clostridiales | Lachnospiraceae | Blautia | Blautia_unclassified |
| Otu01359 | Bacteroidetes | Flavobacteriia | Flavobacteriales | Flavobacteriaceae | Flavobacterium | Flavobacterium_araucananum |
| Otu01360 | Proteobacteria | Deltaproteobacteria | Myxococcales | Polyangiaceae | Sorangium | Sorangium_unclassified |
| Otu01361 | Bacteroidetes | Flavobacteriia | Flavobacteriales | Cryomorphaceae | Fluviicola | Fluviicola_unclassified |
| Otu01362 | Firmicutes | Bacilli | Bacillales | Planococcaceae | Sporosarcina | Sporosarcina_unclassified |
| Otu01369 | Proteobacteria | Betaproteobacteria | Burkholderiales | Oxalobacteraceae | Duganella | Duganella_zoogloeoides |
| Otu01371 | Bacteroidetes | Sphingobacteriia | Sphingobacteriales | Sphingobacteriaceae | Pedobacter | Pedobacter_nutrimenti |
| Otu01370 | Proteobacteria | Alphaproteobacteria | Sphingomonadales | Sphingomonadaceae | Rhizorhapis | Sphingomonas_changbaiensis |
| Otu01378 | Firmicutes | Clostridia | Clostridiales | Lachnospiraceae | Blautia | Blautia_hydrogenotrophica |
| Otu01387 | Firmicutes | Clostridia | Clostridiales | Clostridiaceae 1 | Clostridium sensu stricto | Clostridium sensu stricto_unclassified |
| Otu01376 | Bacteroidetes | Sphingobacteriia | Sphingobacteriales | Sphingobacteriaceae | Mucilaginibacter | Mucilaginibacter_unclassified |
| Otu01385 | Proteobacteria | Betaproteobacteria | Sulfuricellales | Sulfuricellaceae | Sulfuricella | Sulfuricella_unclassified |
| Otu01392 | Firmicutes | Bacilli | Lactobacillales | Aerococcaceae | Facklamia | Facklamia_unclassified |
| Otu01388 | Bacteroidetes | Sphingobacteriia | Sphingobacteriales | Chitinophagaceae | Ferruginibacter | Ferruginibacter_unclassified |
| Otu01390 | Proteobacteria | Betaproteobacteria | Burkholderiales | Comamonadaceae | Simplicispira | Simplicispira_metamorpha |
| Otu01398 | Proteobacteria | Gammaproteobacteria | Gammaproteobacteria_incertae_sedis | Eionea | Eionea | Eionea_unclassified |
| Otu01397 | Firmicutes | Bacilli | Bacillales | Planococcaceae | Planomicrobium | Planomicrobium_unclassified |
| Otu01406 | Actinobacteria | Actinobacteria | Actinomycetales | Micrococcaceae | Arthrobacter | Arthrobacter_arilaitensis |
| Otu01407 | Bacteroidetes | Bacteroidia | Bacteroidales | Porphyromonadaceae | Dysgonomonas | Dysgonomonas_unclassified |
| Otu01415 | Actinobacteria | Actinobacteria | Acidimicrobiales | Iamiaceae | Aquihabitans | Aquihabitans_unclassified |
| Otu01416 | Proteobacteria | Gammaproteobacteria | Legionellales | Coxiellaceae | Diplorickettsia | Diplorickettsia_unclassified |
| Otu01410 | Bacteroidetes | Flavobacteriia | Flavobacteriales | Flavobacteriaceae | Flavobacterium | Flavobacterium_unclassified |
| Otu01411 | Proteobacteria | Alphaproteobacteria | Rhizobiales | Hyphomicrobiaceae | Rhodoplanes | Rhodoplanes_unclassified |
| Otu01417 | Proteobacteria | Betaproteobacteria | Burkholderiales | Oxalobacteraceae | Duganella | Duganella_zoogloeoides |
| Otu01418 | Bacteroidetes | Flavobacteriia | Flavobacteriales | Flavobacteriaceae | Flavobacterium | Flavobacterium_flevense |
| Otu01424 | Proteobacteria | Gammaproteobacteria | Xanthomonadales | Sinobacteraceae | Povalibacter | Povalibacter_unclassified |
| Otu01426 | Proteobacteria | Alphaproteobacteria | Rhodobacterales | Rhodobacteraceae | Haematobacter | Haematobacter_unclassified |
| Otu01440 | Bacteroidetes | Flavobacteriia | Flavobacteriales | Flavobacteriaceae | Chryseobacterium | Chryseobacterium_hominis |
| Otu01436 | Bacteroidetes | Cytophagia | Cytophagales | Cytophagaceae | Dyadobacter | Dyadobacter_unclassified |
| Otu01439 | Proteobacteria | Betaproteobacteria | Burkholderiales | Oxalobacteraceae | Janthinobacterium | Janthinobacterium_lividum |
| Otu01437 | Firmicutes | Bacilli | Bacillales | Thermoactinomycetaceae 1 | Lihuaxuella | Lihuaxuella_unclassified |
| Otu01431 | Proteobacteria | Alphaproteobacteria | Sphingomonadales | Sphingomonadaceae | Sphingomonas | Sphingomonas_qilianensis |
| Otu01442 | Proteobacteria | Betaproteobacteria | Neisseriales | Neisseriaceae | Aquaspirillum | Aquaspirillum_arcticum |
| Otu01446 | Bacteroidetes | Flavobacteriia | Flavobacteriales | Flavobacteriaceae | Flavobacterium | Flavobacterium_unclassified |
| Otu01441 | Bacteroidetes | Sphingobacteriia | Sphingobacteriales | Sphingobacteriaceae | Pedobacter | Pedobacter_unclassified |
| Otu01445 | Proteobacteria | Betaproteobacteria | Rhodocyclales | Rhodocyclaceae | Zoogloea | Zoogloea_oleivorans |
| Otu01452 | Proteobacteria | Deltaproteobacteria | Myxococcales | Polyangiaceae | Byssovorax | Byssovorax_unclassified |
| Otu01457 | Bacteroidetes | Bacteroidia | Bacteroidales | Porphyromonadaceae | Paludibacter | Paludibacter_unclassified |
| Otu01463 | Proteobacteria | Betaproteobacteria | Rhodocyclales | Rhodocyclaceae | Ferribacterium | Ferribacterium_unclassified |
| Otu01461 | Bacteroidetes | Flavobacteriia | Flavobacteriales | Flavobacteriaceae | Flavobacterium | Flavobacterium_algicola |
| Otu01462 | Bacteroidetes | Sphingobacteriia | Sphingobacteriales | Sphingobacteriaceae | Pedobacter | Pedobacter_petrophilus |
| Otu01459 | Proteobacteria | Alphaproteobacteria | Rhizobiales | Rhizobiaceae | Rhizobium | Rhizobium_flavum |
| Otu01460 | Proteobacteria | Betaproteobacteria | Burkholderiales | Comamonadaceae | Rhodoferax | Rhodoferax_unclassified |
| Otu01468 | Actinobacteria | Actinobacteria | Actinomycetales | Microbacteriaceae | Leucobacter | Leucobacter_unclassified |
| Otu01467 | Proteobacteria | Betaproteobacteria | Burkholderiales | Comamonadaceae | Polaromonas | Polaromonas_naphthalenivorans |
| Otu01469 | Proteobacteria | Alphaproteobacteria | Sphingomonadales | Sphingomonadaceae | Sphingobium | Sphingobium_qiguonii |
| Otu01476 | Actinobacteria | Actinobacteria | Actinomycetales | Dermabacteraceae | Devriesea | Devriesea_unclassified |
| Otu01478 | Bacteroidetes | Cytophagia | Cytophagales | Cytophagaceae | Emticicia | Emticicia_oligotrophica |
| Otu01477 | Proteobacteria | Betaproteobacteria | Rhodocyclales | Rhodocyclaceae | Ferribacterium | Ferribacterium_unclassified |
| Otu01472 | Bacteroidetes | Sphingobacteriia | Sphingobacteriales | Saprospiraceae | Haliscomenobacter | Haliscomenobacter_unclassified |
| Otu01475 | Proteobacteria | Betaproteobacteria | Burkholderiales | Burkholderiales_incertae_sedis | Sphaerotilus | Sphaerotilus_montanus |
| Otu01487 | Proteobacteria | Betaproteobacteria | Burkholderiales | Oxalobacteraceae | Janthinobacterium | Janthinobacterium_unclassified |
| Otu01481 | Proteobacteria | Betaproteobacteria | Burkholderiales | Comamonadaceae | Pseudorhodoferax | Pseudorhodoferax_unclassified |
| Otu01494 | Actinobacteria | Actinobacteria | Coriobacteriales | Coriobacteriaceae | Enterorhabdus | Enterorhabdus_unclassified |
| Otu01501 | Bacteroidetes | Bacteroidia | Bacteroidales | Bacteroidaceae | Bacteroides | Bacteroides_unclassified |
| Otu01500 | Firmicutes | Clostridia | Clostridiales | Lachnospiraceae | Blautia | Blautia_unclassified |
| Otu01502 | Proteobacteria | Alphaproteobacteria | Rhizobiales | Methylobacteriaceae | Methylobacterium | Methylobacterium_longum |
| Otu01495 | Bacteroidetes | Cytophagia | Cytophagales | Cyclobacteriaceae | Nitritalea | Nitritalea_unclassified |
| Otu01497 | Bacteroidetes | Cytophagia | Cytophagales | Cytophagaceae | Spirosoma | Spirosoma_unclassified |
| Otu01510 | Proteobacteria | Gammaproteobacteria | Pseudomonadales | Moraxellaceae | Acinetobacter | Acinetobacter_lwoffii |
| Otu01506 | Bacteroidetes | Flavobacteriia | Flavobacteriales | Flavobacteriaceae | Flavobacterium | Flavobacterium_unclassified |
| Otu01511 | Proteobacteria | Betaproteobacteria | Burkholderiales | Oxalobacteraceae | Janthinobacterium | Janthinobacterium_unclassified |
| Otu01504 | Proteobacteria | Alphaproteobacteria | Caulobacterales | Caulobacteraceae | Phenylobacterium | Phenylobacterium_lituiforme |
| Otu01505 | Proteobacteria | Alphaproteobacteria | Sphingomonadales | Erythrobacteraceae | Porphyrobacter | Porphyrobacter_donghaensis |
| Otu01517 | Firmicutes | Negativicutes | Selenomonadales | Veillonellaceae | Mitsuokella | Mitsuokella_multacida |
| Otu01513 | Bacteroidetes | Sphingobacteriia | Sphingobacteriales | Sphingobacteriaceae | Mucilaginibacter | Mucilaginibacter_soyangensis |
| Otu01526 | Bacteroidetes | Flavobacteriia | Flavobacteriales | Flavobacteriaceae | Flavobacterium | Flavobacterium_unclassified |
| Otu01525 | Proteobacteria | Alphaproteobacteria | Rhizobiales | Hyphomicrobiaceae | Hyphomicrobium | Hyphomicrobium_denitrificans |
| Otu01527 | Bacteroidetes | Sphingobacteriia | Sphingobacteriales | Sphingobacteriaceae | Mucilaginibacter | Mucilaginibacter_unclassified |
| Otu01528 | Proteobacteria | Betaproteobacteria | Rhodocyclales | Rhodocyclaceae | Dechloromonas | Dechloromonas_unclassified |
| Otu01535 | Proteobacteria | Gammaproteobacteria | Pseudomonadales | Moraxellaceae | Acinetobacter | Acinetobacter_harbinensis |
| Otu01536 | Actinobacteria | Actinobacteria | Actinomycetales | Microbacteriaceae | Salinibacterium | Salinibacterium_unclassified |
| Otu01548 | Actinobacteria | Actinobacteria | Actinomycetales | Micrococcaceae | Arthrobacter | Arthrobacter_unclassified |
| Otu01544 | Proteobacteria | Betaproteobacteria | Burkholderiales | Comamonadaceae | Simplicispira | Simplicispira_psychrophila |
| Otu01540 | Proteobacteria | Alphaproteobacteria | Sphingomonadales | Sphingomonadaceae | Sphingomonas | Sphingomonas_hengshuiensis |
| Otu01559 | Proteobacteria | Gammaproteobacteria | Pseudomonadales | Moraxellaceae | Acinetobacter | Acinetobacter_albensis |
| Otu01554 | Actinobacteria | Actinobacteria | Actinomycetales | Micromonosporaceae | Actinoplanes | Actinoplanes_unclassified |
| Otu01558 | Firmicutes | Bacilli | Lactobacillales | Aerococcaceae | Facklamia | Facklamia_unclassified |
| Otu01556 | Bacteroidetes | Cytophagia | Cytophagales | Cytophagaceae | Flectobacillus | Flectobacillus_lacus |
| Otu01552 | Actinobacteria | Actinobacteria | Actinomycetales | Microbacteriaceae | Microbacterium | Microbacterium_lacticum |
| Otu01555 | Actinobacteria | Actinobacteria | Actinomycetales | Microbacteriaceae | Naasia | Naasia_unclassified |
| Otu01551 | Proteobacteria | Betaproteobacteria | Burkholderiales | Comamonadaceae | Ramlibacter | Ramlibacter_ginsenosidimutans |
| Otu01557 | Bacteroidetes | Cytophagia | Cytophagales | Cytophagaceae | Runella | Runella_unclassified |
| Otu01561 | Chloroflexi | Anaerolineae | Anaerolineales | Anaerolineaceae | Bellilinea | Bellilinea_unclassified |
| Otu01563 | Bacteroidetes | Cytophagia | Cytophagales | Chryseolinea | Chryseolinea | Chryseolinea_unclassified |
| Otu01564 | Bacteroidetes | Bacteroidia | Bacteroidales | Porphyromonadaceae | Dysgonomonas | Dysgonomonas_unclassified |
| Otu01568 | Proteobacteria | Alphaproteobacteria | Caulobacterales | Caulobacteraceae | Brevundimonas | Brevundimonas_unclassified |
| Otu01566 | Proteobacteria | Deltaproteobacteria | Desulfuromonadales | Geobacteraceae | Geobacter | Geobacter_psychrophilus |
| Otu01565 | Actinobacteria | Actinobacteria | Actinomycetales | Microbacteriaceae | Microbacterium | Microbacterium_unclassified |
| Otu01569 | Proteobacteria | Gammaproteobacteria | Pseudomonadales | Pseudomonadaceae | Pseudomonas | Pseudomonas_caeni |
| Otu01582 | Firmicutes | Clostridia | Clostridiales | Clostridiaceae 1 | Clostridium sensu stricto | Clostridium sensu stricto_unclassified |
| Otu01579 | Firmicutes | Clostridia | Clostridiales | Clostridiaceae 1 | Clostridium sensu stricto | Clostridium_bowmanii |
| Otu01577 | Bacteroidetes | Cytophagia | Cytophagales | Cytophagaceae | Dyadobacter | Dyadobacter_hamtensis |
| Otu01584 | Bacteroidetes | Flavobacteriia | Flavobacteriales | Flavobacteriaceae | Flavobacterium | Flavobacterium_unclassified |
| Otu01576 | Proteobacteria | Betaproteobacteria | Rhodocyclales | Rhodocyclaceae | Sulfurisoma | Sulfurisoma_unclassified |
| Otu01589 | Bacteroidetes | Flavobacteriia | Flavobacteriales | Flavobacteriaceae | Chryseobacterium | Chryseobacterium_jeonii |
| Otu01588 | Firmicutes | Bacilli | Lactobacillales | Aerococcaceae | Facklamia | Facklamia_unclassified |
| Otu01592 | Proteobacteria | Deltaproteobacteria | Myxococcales | Nannocystaceae | Pseudenhygromyxa | Pseudenhygromyxa_unclassified |
| Otu01598 | Bacteroidetes | Flavobacteriia | Flavobacteriales | Flavobacteriaceae | Flavobacterium | Flavobacterium_unclassified |
| Otu01594 | Bacteroidetes | Flavobacteriia | Flavobacteriales | Flavobacteriaceae | Galbibacter | Galbibacter_unclassified |
| Otu01596 | Proteobacteria | Alphaproteobacteria | Rhizobiales | Xanthobacteraceae | Xanthobacter | Xanthobacter_autotrophicus |
| Otu01614 | Actinobacteria | Actinobacteria | Solirubrobacterales | Conexibacteraceae | Conexibacter | Conexibacter_unclassified |
| Otu01615 | Bacteroidetes | Flavobacteriia | Flavobacteriales | Flavobacteriaceae | Flavobacterium | Flavobacterium_rivuli |
| Otu01624 | Actinobacteria | Actinobacteria | Actinomycetales | Micromonosporaceae | Actinoplanes | Actinoplanes_utahensis |
| Otu01634 | Proteobacteria | Gammaproteobacteria | Enterobacteriales | Yersiniaceae | Ewingella | Ewingella_americana |
| Otu01629 | Bacteroidetes | Flavobacteriia | Flavobacteriales | Flavobacteriaceae | Flavobacterium | Flavobacterium_swingsii |
| Otu01637 | Proteobacteria | Betaproteobacteria | Burkholderiales | Comamonadaceae | Pseudorhodoferax | Pseudorhodoferax_unclassified |
| Otu01642 | Bacteroidetes | Cytophagia | Cytophagales | Cytophagaceae | Cytophaga | Cytophaga_hutchinsonii |
| Otu01639 | Proteobacteria | Betaproteobacteria | Burkholderiales | Oxalobacteraceae | Duganella | Duganella_unclassified |
| Otu01641 | Proteobacteria | Betaproteobacteria | Burkholderiales | Alcaligenaceae | Eoetvoesia | Eoetvoesia_caeni |
| Otu01647 | Bacteroidetes | Flavobacteriia | Flavobacteriales | Flavobacteriaceae | Flavobacterium | Flavobacterium_cheniae |
| Otu01646 | Bacteroidetes | Flavobacteriia | Flavobacteriales | Flavobacteriaceae | Flavobacterium | Flavobacterium_unclassified |
| Otu01638 | Proteobacteria | Betaproteobacteria | Burkholderiales | Burkholderiales_incertae_sedis | Piscinibacter | Piscinibacter_aquaticus |
| Otu01655 | Actinobacteria | Actinobacteria | Actinomycetales | Corynebacteriaceae | Corynebacterium | Corynebacterium_glutamicum |
| Otu01649 | Bacteroidetes | Sphingobacteriia | Sphingobacteriales | Chitinophagaceae | Crenotalea | Crenotalea_unclassified |
| Otu01653 | Firmicutes | Bacilli | Bacillales | Planococcaceae | Jeotgalibacillus | Jeotgalibacillus_unclassified |
| Otu01658 | Proteobacteria | Gammaproteobacteria | Xanthomonadales | Xanthomonadaceae | Luteibacter | Luteibacter_yeojuensis |
| Otu01654 | Proteobacteria | Alphaproteobacteria | Alphaproteobacteria_incertae_sedis | Rhizomicrobium | Rhizomicrobium | Rhizomicrobium_unclassified |
| Otu01666 | Proteobacteria | Gammaproteobacteria | Pseudomonadales | Moraxellaceae | Acinetobacter | Acinetobacter_unclassified |
| Otu01669 | Bacteroidetes | Flavobacteriia | Flavobacteriales | Flavobacteriaceae | Flavobacterium | Flavobacterium_unclassified |
| Otu01677 | Bacteroidetes | Cytophagia | Cytophagales | Cytophagaceae | Flectobacillus | Flectobacillus_unclassified |
| Otu01672 | Bacteroidetes | Sphingobacteriia | Sphingobacteriales | Sphingobacteriaceae | Pedobacter | Pedobacter_kyungheensis |
| Otu01674 | Proteobacteria | Alphaproteobacteria | Rhodospirillales | Reyranella | Reyranella | Reyranella_terrae |
| Otu01671 | Proteobacteria | Alphaproteobacteria | Sphingomonadales | Sphingomonadaceae | Sphingopyxis | Sphingopyxis_italica |
| Otu01682 | Actinobacteria | Actinobacteria | Actinomycetales | Microbacteriaceae | Subtercola | Subtercola_unclassified |
| Otu01687 | Bacteroidetes | Cytophagia | Cytophagales | Cytophagaceae | Cytophaga | Cytophaga_unclassified |
| Otu01692 | Bacteroidetes | Sphingobacteriia | Sphingobacteriales | Saprospiraceae | Haliscomenobacter | Haliscomenobacter_unclassified |
| Otu01684 | Actinobacteria | Actinobacteria | Actinomycetales | Microbacteriaceae | Herbiconiux | Herbiconiux_unclassified |
| Otu01690 | Proteobacteria | Alphaproteobacteria | Sphingomonadales | Sphingomonadaceae | Novosphingobium | Novosphingobium_panipatense |
| Otu01688 | Firmicutes | Bacilli | Lactobacillales | Leuconostocaceae | Weissella | Weissella_paramesenteroides |
| Otu01701 | Proteobacteria | Betaproteobacteria | Rhodocyclales | Rhodocyclaceae | Ferribacterium | Ferribacterium_unclassified |
| Otu01702 | Bacteroidetes | Flavobacteriia | Flavobacteriales | Flavobacteriaceae | Flavobacterium | Flavobacterium_unclassified |
| Otu01700 | Proteobacteria | Betaproteobacteria | Methylophilales | Methylophilaceae | Methylotenera | Methylotenera_versatilis |
| Otu01706 | Firmicutes | Negativicutes | Selenomonadales | Veillonellaceae | Pelosinus | Pelosinus_fermentans |
| Otu01725 | Actinobacteria | Actinobacteria | Actinomycetales | Microbacteriaceae | Agreia | Agreia_unclassified |
| Otu01722 | Firmicutes | Bacilli | Bacillales | Planococcaceae | Caryophanon | Caryophanon_tenue |
| Otu01730 | Actinobacteria | Actinobacteria | Actinomycetales | Microbacteriaceae | Microbacterium | Microbacterium_aerolatum |
| Otu01728 | Proteobacteria | Betaproteobacteria | Burkholderiales | Oxalobacteraceae | Oxalobacter | Oxalobacter_unclassified |
| Otu01733 | Actinobacteria | Actinobacteria | Actinomycetales | Propionibacteriaceae | Propionicimonas | Propionicimonas_unclassified |
| Otu01737 | Armatimonadetes | Chthonomonadetes | Chthonomonadales | Chthonomonadaceae | Chthonomonas/Armatimonadetes_gp3 | Chthonomonas/Armatimonadetes_gp3_unclassified |
| Otu01741 | Bacteroidetes | Flavobacteriia | Flavobacteriales | Flavobacteriaceae | Flavobacterium | Flavobacterium_unclassified |
| Otu01738 | Proteobacteria | Alphaproteobacteria | Caulobacterales | Caulobacteraceae | Phenylobacterium | Phenylobacterium_zucineum |
| Otu01748 | Bacteroidetes | Flavobacteriia | Flavobacteriales | Flavobacteriaceae | Flavobacterium | Flavobacterium_hercynium |
| Otu01747 | Actinobacteria | Actinobacteria | Actinomycetales | Jonesiaceae | Jonesia | Jonesia_unclassified |
| Otu01751 | Bacteroidetes | Sphingobacteriia | Sphingobacteriales | Sphingobacteriaceae | Mucilaginibacter | Mucilaginibacter_calamicampi |
| Otu01758 | Bacteroidetes | Bacteroidia | Bacteroidales | Porphyromonadaceae | Paludibacter | Paludibacter_unclassified |
| Otu01746 | Proteobacteria | Alphaproteobacteria | Rhodobacterales | Rhodobacteraceae | Paracoccus | Paracoccus_alcaliphilus |
| Otu01759 | Spirochaetes | Spirochaetia | Spirochaetales | Spirochaetaceae | Salinispira | Salinispira_unclassified |
| Otu01775 | Bacteroidetes | Flavobacteriia | Flavobacteriales | Flavobacteriaceae | Flavobacterium | Flavobacterium_succinicans |
| Otu01770 | Bacteroidetes | Flavobacteriia | Flavobacteriales | Flavobacteriaceae | Flavobacterium | Flavobacterium_unclassified |
| Otu01773 | Proteobacteria | Betaproteobacteria | Rhodocyclales | Rhodocyclaceae | Georgfuchsia | Georgfuchsia_unclassified |
| Otu01767 | Acidobacteria | Acidobacteria_Gp5 | Gp5 | Gp5 | Gp5 | Gp5_unclassified |
| Otu01768 | Bacteroidetes | Flavobacteriia | Flavobacteriales | Flavobacteriaceae | Zhouia | Zhouia_unclassified |
| Otu01782 | Fusobacteria | Fusobacteriia | Fusobacteriales | Fusobacteriaceae | Cetobacterium | Cetobacterium_unclassified |
| Otu01779 | Actinobacteria | Actinobacteria | Actinomycetales | Actinomycetaceae | Flaviflexus | Flaviflexus_unclassified |
| Otu01784 | Proteobacteria | Betaproteobacteria | Burkholderiales | Oxalobacteraceae | Undibacterium | Undibacterium_parvum |
| Otu01794 | Proteobacteria | Gammaproteobacteria | Aeromonadales | Aeromonadaceae | Aeromonas | Aeromonas_bestiarum |
| Otu01796 | Proteobacteria | Alphaproteobacteria | Rhizobiales | Hyphomicrobiaceae | Devosia | Devosia_unclassified |
| Otu01788 | Proteobacteria | Betaproteobacteria | Methylophilales | Methylophilaceae | Methylotenera | Methylotenera_versatilis |
| Otu01789 | Proteobacteria | Betaproteobacteria | Burkholderiales | Comamonadaceae | Polaromonas | Polaromonas_jejuensis |
| Otu01807 | Firmicutes | Bacilli | Lactobacillales | Carnobacteriaceae | Atopostipes | Atopostipes_suicloacalis |
| Otu01798 | Bacteroidetes | Flavobacteriia | Flavobacteriales | Flavobacteriaceae | Chryseobacterium | Chryseobacterium_chaponense |
| Otu01805 | Proteobacteria | Deltaproteobacteria | Bdellovibrionales | Bacteriovoracaceae | Peredibacter | Peredibacter_unclassified |
| Otu01804 | Spirochaetes | Spirochaetia | Spirochaetales | Spirochaetaceae | Sphaerochaeta | Sphaerochaeta_unclassified |
| Otu01817 | Proteobacteria | Alphaproteobacteria | Caulobacterales | Caulobacteraceae | Caulobacter | Caulobacter_unclassified |
| Otu01812 | Bacteroidetes | Sphingobacteriia | Sphingobacteriales | Chitinophagaceae | Flavihumibacter | Flavihumibacter_unclassified |
| Otu01821 | Actinobacteria | Actinobacteria | Actinomycetales | Nocardioidaceae | Marmoricola | Marmoricola_aequoreus |
| Otu01814 | Proteobacteria | Alphaproteobacteria | Sphingomonadales | Sphingomonadaceae | Novosphingobium | Novosphingobium_lentum |
| Otu01815 | Proteobacteria | Alphaproteobacteria | Sphingomonadales | Sphingomonadaceae | Sphingomonas | Sphingomonas_oligophenolica |
| Otu01826 | Acidobacteria | Acidobacteria_Gp6 | Gp6 | Gp6 | Gp6 | Gp6_unclassified |
| Otu01831 | Proteobacteria | Betaproteobacteria | Burkholderiales | Comamonadaceae | Ottowia | Ottowia_unclassified |
| Otu01829 | Bacteroidetes | Sphingobacteriia | Sphingobacteriales | Sphingobacteriaceae | Pedobacter | Pedobacter_boryungensis |
| Otu01852 | Bacteroidetes | Cytophagia | Cytophagales | Chryseolinea | Chryseolinea | Chryseolinea_unclassified |
| Otu01851 | Actinobacteria | Actinobacteria | Actinomycetales | Corynebacteriaceae | Corynebacterium | Corynebacterium_faecale |
| Otu01838 | Firmicutes | Bacilli | Lactobacillales | Aerococcaceae | Facklamia | Facklamia_unclassified |
| Otu01849 | Actinobacteria | Actinobacteria | Gaiellales | Gaiellaceae | Gaiella | Gaiella_occulta |
| Otu01836 | Proteobacteria | Gammaproteobacteria | Legionellales | Legionellaceae | Legionella | Legionella_unclassified |
| Otu01864 | Actinobacteria | Actinobacteria | Actinomycetales | Micrococcaceae | Arthrobacter | Arthrobacter_oxydans |
| Otu01873 | Firmicutes | Bacilli | Bacillales | Bacillaceae 1 | Bacillus | Bacillus_unclassified |
| Otu01860 | Proteobacteria | Betaproteobacteria | Burkholderiales | Comamonadaceae | Comamonas | Comamonas_jiangduensis |
| Otu01876 | Proteobacteria | Betaproteobacteria | Burkholderiales | Oxalobacteraceae | Duganella | Duganella_unclassified |
| Otu01874 | Bacteroidetes | Cytophagia | Cytophagales | Cytophagaceae | Dyadobacter | Dyadobacter_ginsengisoli |
| Otu01869 | Bacteroidetes | Flavobacteriia | Flavobacteriales | Flavobacteriaceae | Flavobacterium | Flavobacterium_unclassified |
| Otu01867 | Actinobacteria | Actinobacteria | Gaiellales | Gaiellaceae | Gaiella | Gaiella_unclassified |
| Otu01871 | Spirochaetes | Spirochaetia | Spirochaetales | Spirochaetaceae | Sphaerochaeta | Sphaerochaeta_globosa |
| Otu01858 | Proteobacteria | Alphaproteobacteria | Sphingomonadales | Sphingomonadaceae | Sphingomonas | Sphingomonas_indica |
| Otu01870 | Actinobacteria | Actinobacteria | Actinomycetales | Microbacteriaceae | Subtercola | Subtercola_boreus |
| Otu01896 | Bacteroidetes | Cytophagia | Cytophagales | Cytophagaceae | Emticicia | Emticicia_paludis |
| Otu01893 | Bacteroidetes | Flavobacteriia | Flavobacteriales | Flavobacteriaceae | Flavobacterium | Flavobacterium_unclassified |
| Otu01891 | Proteobacteria | Deltaproteobacteria | Myxococcales | Polyangiaceae | Jahnella | Jahnella_unclassified |
| Otu01884 | Bacteroidetes | Sphingobacteriia | Sphingobacteriales | Sphingobacteriaceae | Mucilaginibacter | Mucilaginibacter_unclassified |
| Otu01886 | Proteobacteria | Alphaproteobacteria | Rhizobiales | Hyphomicrobiaceae | Pelagibacterium | Pelagibacterium_unclassified |
| Otu01895 | Proteobacteria | Alphaproteobacteria | Rhizobiales | Rhizobiaceae | Rhizobium | Rhizobium_huautlense |
| Otu01900 | Proteobacteria | Alphaproteobacteria | Rhizobiales | Hyphomicrobiaceae | Hyphomicrobium | Hyphomicrobium_unclassified |
| Otu01911 | Proteobacteria | Deltaproteobacteria | Myxococcales | Polyangiaceae | Jahnella | Jahnella_unclassified |
| Otu01902 | Actinobacteria | Actinobacteria | Actinomycetales | Microbacteriaceae | Leucobacter | Leucobacter_unclassified |
| Otu01905 | Proteobacteria | Betaproteobacteria | Methylophilales | Methylophilaceae | Methylotenera | Methylotenera_versatilis |
| Otu01899 | Bacteroidetes | Sphingobacteriia | Sphingobacteriales | Sphingobacteriaceae | Pedobacter | Pedobacter_koreensis |
| Otu01907 | Proteobacteria | Alphaproteobacteria | Rhizobiales | Bradyrhizobiaceae | Rhodopseudomonas | Rhodopseudomonas_palustris |
| Otu01922 | Bacteroidetes | Cytophagia | Cytophagales | Cytophagaceae | Arcicella | Arcicella_rigui |
| Otu01925 | Firmicutes | Clostridia | Clostridiales | Lachnospiraceae | Eisenbergiella | Eisenbergiella_unclassified |
| Otu01932 | Proteobacteria | Betaproteobacteria | Burkholderiales | Alcaligenaceae | Parapusillimonas | Parapusillimonas_unclassified |
| Otu01921 | Chloroflexi | Anaerolineae | Anaerolineales | Anaerolineaceae | Pelolinea | Pelolinea_unclassified |
| Otu01942 | Bacteroidetes | Flavobacteriia | Flavobacteriales | Flavobacteriaceae | Chryseobacterium | Chryseobacterium_hominis |
| Otu01947 | Proteobacteria | Deltaproteobacteria | Myxococcales | Polyangiaceae | Sorangium | Sorangium_unclassified |
| Otu01968 | Bacteroidetes | Sphingobacteriia | Sphingobacteriales | Chitinophagaceae | Ferruginibacter | Ferruginibacter_alkalilentus |
| Otu01979 | Actinobacteria | Actinobacteria | Actinomycetales | Jonesiaceae | Jonesia | Jonesia_unclassified |
| Otu01976 | Proteobacteria | Betaproteobacteria | Methylophilales | Methylophilaceae | Methylotenera | Methylotenera_unclassified |
| Otu01969 | Bacteroidetes | Sphingobacteriia | Sphingobacteriales | Sphingobacteriaceae | Mucilaginibacter | Mucilaginibacter_psychrotolerans |
| Otu01957 | Bacteroidetes | Cytophagia | Cytophagales | Cyclobacteriaceae | Nitritalea | Nitritalea_unclassified |
| Otu01964 | Proteobacteria | Betaproteobacteria | Burkholderiales | Comamonadaceae | Polaromonas | Polaromonas_unclassified |
| Otu01954 | Candidatus Saccharibacteria | Saccharibacteria_genera_incertae_sedis | Saccharibacteria_genera_incertae_sedis | Saccharibacteria_genera_incertae_sedis | Saccharibacteria_genera_incertae_sedis | Saccharibacteria_genera_incertae_sedis_unclassified |
| Otu01958 | Firmicutes | Bacilli | Bacillales | Thermoactinomycetaceae 1 | Thermoflavimicrobium | Thermoflavimicrobium_unclassified |
| Otu01972 | Proteobacteria | Betaproteobacteria | Burkholderiales | Oxalobacteraceae | Undibacterium | Undibacterium_terreum |
| Otu01975 | Bacteroidetes | Flavobacteriia | Flavobacteriales | Flavobacteriaceae | Zhouia | Zhouia_unclassified |
| Otu01995 | Firmicutes | Clostridia | Clostridiales | Lachnospiraceae | Anaerostipes | Anaerostipes_hadrus |
| Otu01997 | Firmicutes | Clostridia | Clostridiales | Lachnospiraceae | Blautia | Blautia_unclassified |
| Otu01989 | Firmicutes | Clostridia | Clostridiales | Clostridiaceae 1 | Clostridium sensu stricto | Clostridium sensu stricto_unclassified |
| Otu01981 | Bacteroidetes | Flavobacteriia | Flavobacteriales | Flavobacteriaceae | Flavobacterium | Flavobacterium_unclassified |
| Otu01991 | Bacteroidetes | Flavobacteriia | Flavobacteriales | Flavobacteriaceae | Flavobacterium | Flavobacterium_unclassified |
| Otu01994 | Proteobacteria | Gammaproteobacteria | Legionellales | Legionellaceae | Legionella | Legionella_unclassified |
| Otu01982 | Actinobacteria | Actinobacteria | Actinomycetales | Nocardioidaceae | Nocardioides | Nocardioides_alpinus |
| Otu01986 | Proteobacteria | Betaproteobacteria | Burkholderiales | Oxalobacteraceae | Noviherbaspirillum | Noviherbaspirillum_suwonense |
| Otu02020 | Proteobacteria | Betaproteobacteria | Burkholderiales | Alcaligenaceae | Basilea | Basilea_unclassified |
| Otu02023 | Firmicutes | Clostridia | Clostridiales | Clostridiaceae 1 | Clostridium sensu stricto | Clostridium_tagluense |
| Otu02009 | Actinobacteria | Actinobacteria | Actinomycetales | Micromonosporaceae | Micromonospora | Micromonospora_sonneratiae |
| Otu02013 | Proteobacteria | Betaproteobacteria | Burkholderiales | Comamonadaceae | Ottowia | Ottowia_unclassified |
| Otu02008 | Proteobacteria | Alphaproteobacteria | Sphingomonadales | Sphingomonadaceae | Rhizorhabdus | Rhizorhabdus_unclassified |
| Otu02022 | Proteobacteria | Epsilonproteobacteria | Campylobacterales | Helicobacteraceae | Sulfuricurvum | Sulfuricurvum_unclassified |
| Otu02035 | Proteobacteria | Gammaproteobacteria | Pseudomonadales | Moraxellaceae | Alkanindiges | Alkanindiges_unclassified |
| Otu02028 | Acidobacteria | Acidobacteria_Gp1 | Bryocella | Bryocella | Bryocella | Bryocella_unclassified |
| Otu02034 | Chloroflexi | Anaerolineae | Anaerolineales | Anaerolineaceae | Ornatilinea | Ornatilinea_unclassified |
| Otu02040 | Actinobacteria | Actinobacteria | Actinomycetales | Microbacteriaceae | Schumannella | Schumannella_unclassified |
| Otu02048 | Proteobacteria | Alphaproteobacteria | Rhodospirillales | Acetobacteraceae | Humitalea | Humitalea_unclassified |
| Otu02046 | Proteobacteria | Alphaproteobacteria | Rhodobacterales | Rhodobacteraceae | Jannaschia | Jannaschia_unclassified |
| Otu02057 | Chloroflexi | Chloroflexia | Kallotenuales | Kallotenuaceae | Kallotenue | Kallotenue_unclassified |
| Otu02052 | Candidatus Saccharibacteria | Saccharibacteria_genera_incertae_sedis | Saccharibacteria_genera_incertae_sedis | Saccharibacteria_genera_incertae_sedis | Saccharibacteria_genera_incertae_sedis | Saccharibacteria_genera_incertae_sedis_unclassified |
| Otu02060 | Actinobacteria | Actinobacteria | Actinomycetales | Micromonosporaceae | Verrucosispora | Verrucosispora_maris |
| Otu02078 | Proteobacteria | Alphaproteobacteria | Caulobacterales | Caulobacteraceae | Asticcacaulis | Asticcacaulis_biprosthecium |
| Otu02073 | Actinobacteria | Actinobacteria | Actinomycetales | Dietziaceae | Dietzia | Dietzia_alimentaria |
| Otu02067 | Bacteroidetes | Flavobacteriia | Flavobacteriales | Flavobacteriaceae | Flavobacterium | Flavobacterium_unclassified |
| Otu02076 | Actinobacteria | Actinobacteria | Gaiellales | Gaiellaceae | Gaiella | Gaiella_unclassified |
| Otu02062 | Actinobacteria | Actinobacteria | Actinomycetales | Microbacteriaceae | Leucobacter | Leucobacter_unclassified |
| Otu02070 | Proteobacteria | Gammaproteobacteria | Oceanospirillales | Oceanospirillaceae | Marinospirillum | Marinospirillum_unclassified |
| Otu02064 | Proteobacteria | Alphaproteobacteria | Sphingomonadales | Sphingomonadaceae | Novosphingobium | Novosphingobium_aromaticivorans |
| Otu02066 | Bacteroidetes | Sphingobacteriia | Sphingobacteriales | Sphingobacteriaceae | Pedobacter | Pedobacter_unclassified |
| Otu02065 | Proteobacteria | Alphaproteobacteria | Rhodobacterales | Rhodobacteraceae | Pseudorhodobacter | Pseudorhodobacter_unclassified |
| Otu02074 | Actinobacteria | Actinobacteria | Actinomycetales | Streptosporangiaceae | Thermocatellispora | Thermocatellispora_unclassified |
| Otu02080 | Acidobacteria | Acidobacteria_Gp6 | Gp6 | Gp6 | Gp6 | Gp6_unclassified |
| Otu02093 | Chloroflexi | Anaerolineae | Anaerolineales | Anaerolineaceae | Longilinea | Longilinea_unclassified |
| Otu02094 | Bacteroidetes | Flavobacteriia | Flavobacteriales | Flavobacteriaceae | Myroides | Myroides_unclassified |
| Otu02092 | Proteobacteria | Alphaproteobacteria | Sphingomonadales | Sphingomonadaceae | Novosphingobium | Novosphingobium_aromaticivorans |
| Otu02088 | Proteobacteria | Alphaproteobacteria | Sphingomonadales | Sphingomonadaceae | Novosphingobium | Novosphingobium_capsulatum |
| Otu02112 | Proteobacteria | Betaproteobacteria | Burkholderiales | Burkholderiales_incertae_sedis | Aquabacterium | Aquabacterium_unclassified |
| Otu02110 | Actinobacteria | Actinobacteria | Actinomycetales | Micromonosporaceae | Asanoa | Asanoa_siamensis |
| Otu02107 | Acidobacteria | Acidobacteria_Gp3 | Candidatus Solibacter | Candidatus Solibacter | Candidatus Solibacter | Candidatus Solibacter_unclassified |
| Otu02109 | Actinobacteria | Actinobacteria | Actinomycetales | Corynebacteriaceae | Corynebacterium | Corynebacterium_stationis |
| Otu02108 | Actinobacteria | Actinobacteria | Actinomycetales | Cryptosporangiaceae | Cryptosporangium | Cryptosporangium_unclassified |
| Otu02102 | Firmicutes | Clostridia | Clostridiales | Ruminococcaceae | Faecalibacterium | Faecalibacterium_prausnitzii |
| Otu02116 | Proteobacteria | Gammaproteobacteria | Enterobacteriales | Enterobacteriaceae | Hafnia | Hafnia_unclassified |
| Otu02123 | Bacteroidetes | Cytophagia | Cytophagales | Flammeovirgaceae | Imperialibacter | Imperialibacter_unclassified |
| Otu02124 | Proteobacteria | Alphaproteobacteria | Rhizobiales | Rhizobiales_incertae_sedis | Variibacter | Variibacter_unclassified |
| Otu02141 | Proteobacteria | Alphaproteobacteria | Rhizobiales | Bradyrhizobiaceae | Afipia | Afipia_unclassified |
| Otu02148 | Bacteroidetes | Flavobacteriia | Flavobacteriales | Flavobacteriaceae | Flavobacterium | Flavobacterium_unclassified |
| Otu02143 | Actinobacteria | Actinobacteria | Acidimicrobiales | Iamiaceae | Iamia | Iamia_unclassified |
| Otu02137 | Proteobacteria | Betaproteobacteria | Burkholderiales | Burkholderiales_incertae_sedis | Methylibium | Methylibium_petroleiphilum |
| Otu02129 | Bacteroidetes | Sphingobacteriia | Sphingobacteriales | Saprospiraceae | Portibacter | Portibacter_unclassified |
| Otu02165 | Bacteroidetes | Flavobacteriia | Flavobacteriales | Flavobacteriaceae | Flavobacterium | Flavobacterium_algicola |
| Otu02160 | Bacteroidetes | Flavobacteriia | Flavobacteriales | Flavobacteriaceae | Flavobacterium | Flavobacterium_unclassified |
| Otu02170 | Acidobacteria | Acidobacteria_Gp6 | Gp6 | Gp6 | Gp6 | Gp6_unclassified |
| Otu02169 | Bacteroidetes | Sphingobacteriia | Sphingobacteriales | Chitinophagaceae | Heliimonas | Heliimonas_unclassified |
| Otu02154 | Actinobacteria | Actinobacteria | Actinomycetales | Microbacteriaceae | Microbacterium | Microbacterium_foliorum |
| Otu02155 | Proteobacteria | Gammaproteobacteria | Oceanospirillales | Oceanospirillaceae | Oceanospirillum | Oceanospirillum_unclassified |
| Otu02164 | Bacteroidetes | Sphingobacteriia | Sphingobacteriales | Sphingobacteriaceae | Pedobacter | Pedobacter_alluvionis |
| Otu02159 | Candidatus Saccharibacteria | Saccharibacteria_genera_incertae_sedis | Saccharibacteria_genera_incertae_sedis | Saccharibacteria_genera_incertae_sedis | Saccharibacteria_genera_incertae_sedis | Saccharibacteria_genera_incertae_sedis_unclassified |
| Otu02181 | Actinobacteria | Actinobacteria | Actinomycetales | Nocardioidaceae | Aeromicrobium | Aeromicrobium_unclassified |
| Otu02186 | Bacteroidetes | Sphingobacteriia | Sphingobacteriales | Chitinophagaceae | Cnuella | Cnuella_unclassified |
| Otu02192 | Proteobacteria | Gammaproteobacteria | Methylococcales | Methylococcaceae | Methylomarinum | Methylomarinum_unclassified |
| Otu02182 | Proteobacteria | Betaproteobacteria | Burkholderiales | Oxalobacteraceae | Undibacterium | Undibacterium_jejuense |
| Otu02195 | Bacteroidetes | Flavobacteriia | Flavobacteriales | Flavobacteriaceae | Zhouia | Zhouia_unclassified |
| Otu02212 | Proteobacteria | Gammaproteobacteria | Pseudomonadales | Moraxellaceae | Acinetobacter | Acinetobacter_indicus |
| Otu02211 | Firmicutes | Clostridia | Clostridiales | Clostridiales_Incertae Sedis XIII | Anaerovorax | Anaerovorax_unclassified |
| Otu02217 | Bacteroidetes | Flavobacteriia | Flavobacteriales | Flavobacteriaceae | Chryseobacterium | Chryseobacterium_unclassified |
| Otu02220 | Actinobacteria | Actinobacteria | Actinomycetales | Corynebacteriaceae | Corynebacterium | Corynebacterium_casei |
| Otu02216 | Proteobacteria | Deltaproteobacteria | Myxococcales | Polyangiaceae | Jahnella | Jahnella_unclassified |
| Otu02209 | Proteobacteria | Betaproteobacteria | Burkholderiales | Burkholderiales_incertae_sedis | Methylibium | Methylibium_unclassified |
| Otu02215 | Bacteroidetes | Bacteroidia | Bacteroidales | Porphyromonadaceae | Paludibacter | Paludibacter_unclassified |
| Otu02218 | Proteobacteria | Alphaproteobacteria | Rhizobiales | Rhizobiaceae | Rhizobium | Rhizobium_giardinii |
| Otu02219 | Spirochaetes | Spirochaetia | Spirochaetales | Spirochaetaceae | Salinispira | Salinispira_unclassified |
| Otu02206 | Actinobacteria | Thermoleophilia | Thermoleophilales | Thermoleophilaceae | Thermoleophilum | Thermoleophilum_unclassified |
| Otu02228 | Proteobacteria | Gammaproteobacteria | Pseudomonadales | Moraxellaceae | Acinetobacter | Acinetobacter_kyonggiensis |
| Otu02223 | Firmicutes | Clostridia | Clostridiales | Clostridiaceae 1 | Clostridium sensu stricto | Clostridium_bowmanii |
| Otu02240 | Bacteroidetes | Flavobacteriia | Flavobacteriales | Flavobacteriaceae | Flavobacterium | Flavobacterium_fluvii |
| Otu02229 | Actinobacteria | Actinobacteria | Acidimicrobiales | Acidimicrobiaceae | Ilumatobacter | Ilumatobacter_unclassified |
| Otu02227 | Bacteroidetes | Sphingobacteriia | Sphingobacteriales | Saprospiraceae | Portibacter | Portibacter_unclassified |
| Otu02243 | Firmicutes | Clostridia | Clostridiales | Clostridiaceae 1 | Youngiibacter | Youngiibacter_unclassified |
| Otu02233 | Bacteroidetes | Flavobacteriia | Flavobacteriales | Flavobacteriaceae | Zhouia | Zhouia_unclassified |
| Otu02251 | Acetothermia | Acetothermia_genera_incertae_sedis | Acetothermia_genera_incertae_sedis | Acetothermia_genera_incertae_sedis | Acetothermia_genera_incertae_sedis | Acetothermia_genera_incertae_sedis_unclassified |
| Otu02263 | Actinobacteria | Actinobacteria | Actinomycetales | Micrococcaceae | Arthrobacter | Arthrobacter_antarcticus |
| Otu02260 | Bacteroidetes | Sphingobacteriia | Sphingobacteriales | Chitinophagaceae | Cnuella | Cnuella_unclassified |
| Otu02271 | Bacteroidetes | Flavobacteriia | Flavobacteriales | Flavobacteriaceae | Leptobacterium | Leptobacterium_unclassified |
| Otu02257 | Actinobacteria | Actinobacteria | Actinomycetales | Microbacteriaceae | Leucobacter | Leucobacter_unclassified |
| Otu02265 | Proteobacteria | Alphaproteobacteria | Sphingomonadales | Sphingomonadaceae | Rhizorhabdus | Rhizorhabdus_argentea |
| Otu02254 | Firmicutes | Bacilli | Bacillales | Planococcaceae | Sporosarcina | Sporosarcina_unclassified |
| Otu02276 | Proteobacteria | Alphaproteobacteria | Rhizobiales | Phyllobacteriaceae | Aminobacter | Aminobacter_anthyllidis |
| Otu02287 | Bacteroidetes | Flavobacteriia | Flavobacteriales | Flavobacteriaceae | Flavobacterium | Flavobacterium_psychrolimnae |
| Otu02301 | Bacteroidetes | Flavobacteriia | Flavobacteriales | Flavobacteriaceae | Flavobacterium | Flavobacterium_unclassified |
| Otu02293 | Proteobacteria | Betaproteobacteria | Burkholderiales | Comamonadaceae | Limnohabitans | Limnohabitans_parvus |
| Otu02289 | Proteobacteria | Gammaproteobacteria | Oceanospirillales | Oceanospirillaceae | Marinospirillum | Marinospirillum_megaterium |
| Otu02282 | Actinobacteria | Actinobacteria | Actinomycetales | Bogoriellaceae | Oceanitalea | Oceanitalea_unclassified |
| Otu02288 | Proteobacteria | Deltaproteobacteria | Myxococcales | Polyangiaceae | Sorangium | Sorangium_unclassified |
| Otu02330 | Proteobacteria | Gammaproteobacteria | Xanthomonadales | Xanthomonadaceae | Arenimonas | Arenimonas_unclassified |
| Otu02321 | Bacteroidetes | Sphingobacteriia | Sphingobacteriales | Chitinophagaceae | Cnuella | Cnuella_unclassified |
| Otu02315 | Actinobacteria | Actinobacteria | Actinomycetales | Propionibacteriaceae | Friedmanniella | Friedmanniella_spumicola |
| Otu02310 | Actinobacteria | Actinobacteria | Solirubrobacterales | Patulibacteraceae | Patulibacter | Patulibacter_unclassified |
| Otu02316 | Firmicutes | Negativicutes | Selenomonadales | Veillonellaceae | Psychrosinus | Psychrosinus_unclassified |
| Otu02328 | Proteobacteria | Betaproteobacteria | Neisseriales | Neisseriaceae | Rivicola | Rivicola_unclassified |
| Otu02335 | Bacteroidetes | Sphingobacteriia | Sphingobacteriales | Chitinophagaceae | Ferruginibacter | Ferruginibacter_unclassified |
| Otu02348 | Firmicutes | Clostridia | Clostridiales | Lachnospiraceae | Mobilitalea | Mobilitalea_unclassified |
| Otu02338 | Nitrospirae | Nitrospira | Nitrospirales | Nitrospiraceae | Nitrospira | Nitrospira_unclassified |
| Otu02359 | Proteobacteria | Deltaproteobacteria | Myxococcales | Polyangiaceae | Sorangium | Sorangium_unclassified |
| Otu02354 | Actinobacteria | Actinobacteria | Actinomycetales | Streptosporangiaceae | Thermocatellispora | Thermocatellispora_unclassified |
| Otu02350 | Firmicutes | Clostridia | Clostridiales | Clostridiaceae 1 | Youngiibacter | Youngiibacter_unclassified |
| Otu02367 | Actinobacteria | Actinobacteria | Actinomycetales | Microbacteriaceae | Agrococcus | Agrococcus_unclassified |
| Otu02366 | Proteobacteria | Deltaproteobacteria | Desulfobacterales | Desulfobacteraceae | Desulfatirhabdium | Desulfatirhabdium_unclassified |
| Otu02374 | Bacteroidetes | Flavobacteriia | Flavobacteriales | Flavobacteriaceae | Flavobacterium | Flavobacterium_rivuli |
| Otu02373 | Proteobacteria | Gammaproteobacteria | Oceanospirillales | Halomonadaceae | Halomonas | Halomonas_desiderata |
| Otu02363 | Proteobacteria | Betaproteobacteria | Burkholderiales | Comamonadaceae | Limnohabitans | Limnohabitans_unclassified |
| Otu02376 | Bacteroidetes | Sphingobacteriia | Sphingobacteriales | Sphingobacteriaceae | Mucilaginibacter | Mucilaginibacter_angelicae |
| Otu02385 | Actinobacteria | Actinobacteria | Solirubrobacterales | Patulibacteraceae | Patulibacter | Patulibacter_unclassified |
| Otu02379 | Firmicutes | Bacilli | Bacillales | Planococcaceae | Psychrobacillus | Psychrobacillus_unclassified |
| Otu02398 | Actinobacteria | Actinobacteria | Actinomycetales | Nocardioidaceae | Aeromicrobium | Aeromicrobium_flavum |
| Otu02407 | Actinobacteria | Actinobacteria | Acidimicrobiales | Iamiaceae | Aquihabitans | Aquihabitans_unclassified |
| Otu02404 | Firmicutes | Clostridia | Clostridiales | Lachnospiraceae | Catonella | Catonella_unclassified |
| Otu02415 | Proteobacteria | Alphaproteobacteria | Rhizobiales | Bradyrhizobiaceae | Tardiphaga | Tardiphaga_unclassified |
| Otu02421 | Actinobacteria | Actinobacteria | Actinomycetales | Micromonosporaceae | Actinoplanes | Actinoplanes_octamycinicus |
| Otu02435 | Bacteroidetes | Flavobacteriia | Flavobacteriales | Flavobacteriaceae | Chishuiella | Chishuiella_unclassified |
| Otu02433 | Proteobacteria | Alphaproteobacteria | Rhizobiales | Methylobacteriaceae | Methylobacterium | Methylobacterium_mesophilicum |
| Otu02418 | Actinobacteria | Actinobacteria | Coriobacteriales | Coriobacteriaceae | Olsenella | Olsenella_unclassified |
| Otu02442 | Firmicutes | Bacilli | Lactobacillales | Aerococcaceae | Abiotrophia | Abiotrophia_defectiva |
| Otu02455 | Bacteroidetes | Cytophagia | Cytophagales | Cytophagaceae | Emticicia | Emticicia_fontis |
| Otu02463 | Actinobacteria | Actinobacteria | Actinomycetales | Nakamurellaceae | Nakamurella | Nakamurella_unclassified |
| Otu02449 | Actinobacteria | Actinobacteria | Actinomycetales | Cellulomonadaceae | Oerskovia | Oerskovia_unclassified |
| Otu02457 | Proteobacteria | Alphaproteobacteria | Rhizobiales | Methylocystaceae | Pleomorphomonas | Pleomorphomonas_oryzae |
| Otu02444 | Bacteroidetes | Sphingobacteriia | Sphingobacteriales | Saprospiraceae | Portibacter | Portibacter_unclassified |
| Otu02441 | Actinobacteria | Actinobacteria | Actinomycetales | Propionibacteriaceae | Propioniciclava | Propioniciclava_unclassified |
| Otu02462 | Proteobacteria | Alphaproteobacteria | Sphingomonadales | Sphingomonadaceae | Sphingomonas | Sphingomonas_unclassified |
| Otu02476 | Proteobacteria | Alphaproteobacteria | Rhizobiales | Hyphomicrobiaceae | Devosia | Devosia_chinhatensis |
| Otu02503 | Bacteroidetes | Cytophagia | Cytophagales | Cytophagaceae | Dyadobacter | Dyadobacter_unclassified |
| Otu02498 | Actinobacteria | Actinobacteria | Actinomycetales | Beutenbergiaceae | Serinibacter | Serinibacter_unclassified |
| Otu02508 | Proteobacteria | Alphaproteobacteria | Caulobacterales | Hyphomonadaceae | Marinicauda | Marinicauda_unclassified |
| Otu02531 | Actinobacteria | Actinobacteria | Actinomycetales | Nocardioidaceae | Nocardioides | Nocardioides_unclassified |
| Otu02510 | Proteobacteria | Alphaproteobacteria | Rhodobacterales | Rhodobacteraceae | Paracoccus | Paracoccus_alcaliphilus |
| Otu02559 | Bacteroidetes | Flavobacteriia | Flavobacteriales | Flavobacteriaceae | Corallibacter | Corallibacter_unclassified |
| Otu02589 | Bacteroidetes | Cytophagia | Cytophagales | Cytophagaceae | Cytophaga | Cytophaga_unclassified |
| Otu02570 | Proteobacteria | Alphaproteobacteria | Rhodobacterales | Rhodobacteraceae | Gemmobacter | Gemmobacter_unclassified |
| Otu02563 | Acidobacteria | Acidobacteria_Gp6 | Gp6 | Gp6 | Gp6 | Gp6_unclassified |
| Otu02554 | Chloroflexi | Anaerolineae | Anaerolineales | Anaerolineaceae | Longilinea | Longilinea_unclassified |
| Otu02588 | Actinobacteria | Actinobacteria | Actinomycetales | Demequinaceae | Lysinimicrobium | Lysinimicrobium_unclassified |
| Otu02556 | Actinobacteria | Actinobacteria | Actinomycetales | Mycobacteriaceae | Mycobacterium | Mycobacterium_hodleri |
| Otu02561 | Proteobacteria | Alphaproteobacteria | Rickettsiales | Rickettsiaceae | Orientia | Orientia_unclassified |
| Otu02553 | Proteobacteria | Betaproteobacteria | Burkholderiales | Comamonadaceae | Polaromonas | Polaromonas_unclassified |
| Otu02562 | Proteobacteria | Alphaproteobacteria | Sphingomonadales | Sphingomonadaceae | Sphingorhabdus | Sphingomonas_jatrophae |
| Otu02592 | Proteobacteria | Deltaproteobacteria | Bdellovibrionales | Bacteriovoracaceae | Bacteriovorax | Bacteriovorax_unclassified |
| Otu02611 | Firmicutes | Bacilli | Lactobacillales | Aerococcaceae | Facklamia | Facklamia_unclassified |
| Otu02622 | Firmicutes | Erysipelotrichia | Erysipelotrichales | Erysipelotrichaceae | Holdemanella | Holdemanella_biformis |
| Otu02596 | Actinobacteria | Actinobacteria | Acidimicrobiales | Iamiaceae | Iamia | Iamia_unclassified |
| Otu02597 | Proteobacteria | Alphaproteobacteria | Rhodospirillales | Rhodospirillaceae | Magnetospira | Magnetospira_unclassified |
| Otu02608 | Proteobacteria | Betaproteobacteria | Burkholderiales | Burkholderiales_incertae_sedis | Methylibium | Methylibium_unclassified |
| Otu02594 | Proteobacteria | Alphaproteobacteria | Rhizobiales | Beijerinckiaceae | Methylocapsa | Methylocapsa_unclassified |
| Otu02617 | Bacteroidetes | Bacteroidia | Bacteroidales | Rikenellaceae | Mucinivorans | Mucinivorans_unclassified |
| Otu02602 | Proteobacteria | Deltaproteobacteria | Myxococcales | Polyangiaceae | Polyangium | Polyangium_unclassified |
| Otu02614 | Proteobacteria | Alphaproteobacteria | Sphingomonadales | Sphingomonadaceae | Sphingomonas | Sphingomonas_asaccharolytica |
| Otu02610 | Proteobacteria | Alphaproteobacteria | Sphingomonadales | Sphingomonadaceae | Sphingomonas | Sphingomonas_koreensis |
| Otu02595 | Firmicutes | Clostridia | Clostridiales | Clostridiales_Incertae Sedis XI | Tissierella | Tissierella_unclassified |
| Otu02642 | Bacteroidetes | Flavobacteriia | Flavobacteriales | Flavobacteriaceae | Chryseobacterium | Chryseobacterium_gregarium |
| Otu02645 | Acidobacteria | Acidobacteria_Gp6 | Gp6 | Gp6 | Gp6 | Gp6_unclassified |
| Otu02666 | Actinobacteria | Actinobacteria | Actinomycetales | Nocardioidaceae | Nocardioides | Nocardioides_iriomotensis |
| Otu02636 | Proteobacteria | Alphaproteobacteria | Rhodobacterales | Rhodobacteraceae | Pseudorhodobacter | Pseudorhodobacter_unclassified |
| Otu02673 | Proteobacteria | Alphaproteobacteria | Sphingomonadales | Sphingomonadaceae | Sphingomonas | Sphingomonas_aerolata |
| Otu02659 | Proteobacteria | Deltaproteobacteria | Myxococcales | Vulgatibacteraceae | Vulgatibacter | Vulgatibacter_unclassified |
| Otu02702 | Proteobacteria | Betaproteobacteria | Burkholderiales | Alcaligenaceae | Advenella | Advenella_faeciporci |
| Otu02722 | Firmicutes | Clostridia | Clostridiales | Lachnospiraceae | Blautia | Blautia_unclassified |
| Otu02687 | Firmicutes | Clostridia | Clostridiales | Clostridiaceae 1 | Clostridium sensu stricto | Clostridium sensu stricto_unclassified |
| Otu02682 | Actinobacteria | Actinobacteria | Actinomycetales | Demequinaceae | Demequina | Demequina_unclassified |
| Otu02711 | Bacteroidetes | Flavobacteriia | Flavobacteriales | Cryomorphaceae | Fluviicola | Fluviicola_unclassified |
| Otu02729 | Bacteroidetes | Bacteroidia | Bacteroidales | Marinilabiliaceae | Geofilum | Geofilum_unclassified |
| Otu02703 | Bacteroidetes | Sphingobacteriia | Sphingobacteriales | Saprospiraceae | Haliscomenobacter | Haliscomenobacter_unclassified |
| Otu02708 | Bacteroidetes | Sphingobacteriia | Sphingobacteriales | Sphingobacteriaceae | Pedobacter | Pedobacter_unclassified |
| Otu02681 | Firmicutes | Negativicutes | Selenomonadales | Veillonellaceae | Psychrosinus | Psychrosinus_unclassified |
| Otu02679 | Actinobacteria | Actinobacteria | Actinomycetales | Microbacteriaceae | Rathayibacter | Rathayibacter_tritici |
| Otu02691 | Bacteroidetes | Sphingobacteriia | Sphingobacteriales | Chitinophagaceae | Segetibacter | Segetibacter_unclassified |
| Otu02690 | Proteobacteria | Gammaproteobacteria | Pseudomonadales | Pseudomonadaceae | Serpens | Serpens_unclassified |
| Otu02747 | Bacteroidetes | Flavobacteriia | Flavobacteriales | Flavobacteriaceae | Flavobacterium | Flavobacterium_rivuli |
| Otu02753 | Bacteroidetes | Flavobacteriia | Flavobacteriales | Flavobacteriaceae | Flavobacterium | Flavobacterium_swingsii |
| Otu02746 | Bacteroidetes | Flavobacteriia | Flavobacteriales | Flavobacteriaceae | Flavobacterium | Flavobacterium_unclassified |
| Otu02763 | Bacteroidetes | Flavobacteriia | Flavobacteriales | Flavobacteriaceae | Flavobacterium | Flavobacterium_unclassified |
| Otu02776 | Actinobacteria | Actinobacteria | Acidimicrobiales | Acidimicrobiaceae | Ilumatobacter | Ilumatobacter_unclassified |
| Otu02777 | Actinobacteria | Actinobacteria | Actinomycetales | Microbacteriaceae | Microbacterium | Microbacterium_lacus |
| Otu02779 | Bacteroidetes | Sphingobacteriia | Sphingobacteriales | Sphingobacteriaceae | Mucilaginibacter | Mucilaginibacter_unclassified |
| Otu02756 | Firmicutes | Clostridia | Clostridiales | Ruminococcaceae | Oscillibacter | Oscillibacter_unclassified |
| Otu02770 | Firmicutes | Bacilli | Bacillales | Planococcaceae | Planomicrobium | Planomicrobium_flavidum |
| Otu02751 | Bacteroidetes | Sphingobacteriia | Sphingobacteriales | Saprospiraceae | Portibacter | Portibacter_unclassified |
| Otu02745 | Proteobacteria | Alphaproteobacteria | Rhodospirillales | Acetobacteraceae | Rhodopila | Rhodopila_unclassified |
| Otu02789 | Actinobacteria | Actinobacteria | Actinomycetales | Micromonosporaceae | Actinoplanes | Actinoplanes_durhamensis |
| Otu02810 | Acidobacteria | Acidobacteria_Gp4 | Blastocatella | Blastocatella | Blastocatella | Blastocatella_unclassified |
| Otu02811 | Firmicutes | Clostridia | Clostridiales | Lachnospiraceae | Blautia | Blautia_unclassified |
| Otu02803 | Firmicutes | Clostridia | Clostridiales | Clostridiaceae 1 | Clostridium sensu stricto | Clostridium_vincentii |
| Otu02790 | Actinobacteria | Actinobacteria | Actinomycetales | Microbacteriaceae | Leucobacter | Leucobacter_aerolatus |
| Otu02787 | Actinobacteria | Actinobacteria | Actinomycetales | Nakamurellaceae | Nakamurella | Nakamurella_multipartita |
| Otu02818 | Proteobacteria | Deltaproteobacteria | Myxococcales | Nannocystaceae | Pseudenhygromyxa | Pseudenhygromyxa_unclassified |
| Otu02791 | Firmicutes | Clostridia | Thermoanaerobacterales | Thermodesulfobiaceae | Thermodesulfobium | Thermodesulfobium_unclassified |
| Otu02834 | Acidobacteria | Acidobacteria_Gp4 | Blastocatella | Blastocatella | Blastocatella | Blastocatella_unclassified |
| Otu02849 | Caldiserica | Caldisericia | Caldisericales | Caldisericaceae | Caldisericum | Caldisericum_unclassified |
| Otu02832 | Proteobacteria | Alphaproteobacteria | Rhodobacterales | Rhodobacteraceae | Frigidibacter | Frigidibacter_unclassified |
| Otu02826 | Proteobacteria | Betaproteobacteria | Burkholderiales | Burkholderiales_incertae_sedis | Ideonella | Ideonella_unclassified |
| Otu02840 | Proteobacteria | Gammaproteobacteria | Pseudomonadales | Pseudomonadaceae | Serpens | Serpens_unclassified |
| Otu02825 | Proteobacteria | Alphaproteobacteria | Rhodobacterales | Rhodobacteraceae | Thiobacimonas | Thiobacimonas_unclassified |
| Otu02877 | Firmicutes | Clostridia | Clostridiales | Lachnospiraceae | Blautia | Blautia_unclassified |
| Otu02886 | Proteobacteria | Deltaproteobacteria | Desulfobacterales | Desulfobacteraceae | Desulfatirhabdium | Desulfatirhabdium_unclassified |
| Otu02875 | Firmicutes | Bacilli | Lactobacillales | Lactobacillaceae | Lactobacillus | Lactobacillus_coryniformis |
| Otu02912 | Actinobacteria | Actinobacteria | Actinomycetales | Microbacteriaceae | Leucobacter | Leucobacter_unclassified |
| Otu02879 | Proteobacteria | Betaproteobacteria | Burkholderiales | Comamonadaceae | Rhodoferax | Rhodoferax_saidenbachensis |
| Otu02917 | Actinobacteria | Actinobacteria | Solirubrobacterales | Solirubrobacteraceae | Solirubrobacter | Solirubrobacter_unclassified |
| Otu02894 | Spirochaetes | Spirochaetia | Spirochaetales | Spirochaetaceae | Spirochaeta | Spirochaeta_unclassified |
| Otu02970 | Bacteroidetes | Flavobacteriia | Flavobacteriales | Flavobacteriaceae | Flavobacterium | Flavobacterium_unclassified |
| Otu02944 | Gemmatimonadetes | Gemmatimonadetes | Gemmatimonadales | Gemmatimonadaceae | Gemmatimonas | Gemmatimonas_unclassified |
| Otu02928 | Proteobacteria | Deltaproteobacteria | Myxococcales | Labilitrichaceae | Labilithrix | Labilithrix_unclassified |
| Otu02951 | Bacteroidetes | Cytophagia | Cytophagales | Cytophagaceae | Lacihabitans | Lacihabitans_unclassified |
| Otu02957 | Proteobacteria | Alphaproteobacteria | Rhizobiales | Beijerinckiaceae | Methylorosula | Methylorosula_unclassified |
| Otu02977 | Actinobacteria | Actinobacteria | Actinomycetales | Microbacteriaceae | Microbacterium | Microbacterium_hydrothermale |
| Otu02969 | Bacteroidetes | Bacteroidia | Bacteroidales | Porphyromonadaceae | Paludibacter | Paludibacter_propionicigenes |
| Otu02943 | Proteobacteria | Alphaproteobacteria | Rhizobiales | Rhizobiales_incertae_sedis | Phreatobacter | Phreatobacter_oligotrophus |
| Otu02954 | Proteobacteria | Deltaproteobacteria | Myxococcales | Polyangiaceae | Sorangium | Sorangium_unclassified |
| Otu02987 | Proteobacteria | Betaproteobacteria | Burkholderiales | Burkholderiales_incertae_sedis | Aquabacterium | Aquabacterium_unclassified |
| Otu03042 | Bacteroidetes | Flavobacteriia | Flavobacteriales | Cryomorphaceae | Brumimicrobium | Brumimicrobium_unclassified |
| Otu02990 | Proteobacteria | Betaproteobacteria | Burkholderiales | Oxalobacteraceae | Duganella | Duganella_phyllosphaerae |
| Otu03017 | Firmicutes | Bacilli | Lactobacillales | Carnobacteriaceae | Jeotgalibaca | Jeotgalibaca_arthritidis |
| Otu03036 | Chloroflexi | Caldilineae | Caldilineales | Caldilineaceae | Litorilinea | Litorilinea_unclassified |
| Otu02998 | Proteobacteria | Betaproteobacteria | Burkholderiales | Comamonadaceae | Polaromonas | Polaromonas_aquatica |
| Otu02978 | Bacteroidetes | Sphingobacteriia | Sphingobacteriales | Saprospiraceae | Portibacter | Portibacter_unclassified |
| Otu03013 | Proteobacteria | Epsilonproteobacteria | Campylobacterales | Helicobacteraceae | Sulfuricurvum | Sulfuricurvum_unclassified |
| Otu03018 | Proteobacteria | Betaproteobacteria | Rhodocyclales | Rhodocyclaceae | Sulfuritalea | Sulfuritalea_unclassified |
| Otu03075 | Proteobacteria | Gammaproteobacteria | Xanthomonadales | Xanthomonadaceae | Aspromonas | Aspromonas_unclassified |
| Otu03096 | Firmicutes | Bacilli | Bacillales | Paenibacillaceae 1 | Fontibacillus | Fontibacillus_unclassified |
| Otu03053 | Proteobacteria | Betaproteobacteria | Burkholderiales | Oxalobacteraceae | Janthinobacterium | Janthinobacterium_unclassified |
| Otu03065 | Proteobacteria | Alphaproteobacteria | Rhizobiales | Phyllobacteriaceae | Lentilitoribacter | Lentilitoribacter_unclassified |
| Otu03047 | Verrucomicrobia | Opitutae | Opitutales | Opitutaceae | Opitutus | Opitutus_unclassified |
| Otu03091 | Proteobacteria | Betaproteobacteria | Burkholderiales | Oxalobacteraceae | Oxalobacter | Oxalobacter_unclassified |
| Otu03094 | Bacteroidetes | Bacteroidia | Bacteroidales | Porphyromonadaceae | Proteiniphilum | Proteiniphilum_unclassified |
| Otu03101 | Actinobacteria | Actinobacteria | Actinomycetales | Micromonosporaceae | Actinoplanes | Actinoplanes_unclassified |
| Otu03142 | Bacteroidetes | Flavobacteriia | Flavobacteriales | Flavobacteriaceae | Aequorivita | Aequorivita_unclassified |
| Otu03158 | Firmicutes | Clostridia | Clostridiales | Ruminococcaceae | Butyricicoccus | Butyricicoccus_unclassified |
| Otu03149 | Proteobacteria | Deltaproteobacteria | Desulfovibrionales | Desulfovibrionaceae | Desulfovibrio | Desulfovibrio_idahonensis |
| Otu03148 | Proteobacteria | Alphaproteobacteria | Magnetococcales | Magnetococcaceae | Magnetococcus | Magnetococcus_unclassified |
| Otu03105 | Proteobacteria | Deltaproteobacteria | Myxococcales | Nannocystaceae | Plesiocystis | Plesiocystis_pacifica |
| Otu03099 | Firmicutes | Bacilli | Bacillales | Planococcaceae | Sporosarcina | Sporosarcina_unclassified |
| Otu03232 | Proteobacteria | Epsilonproteobacteria | Campylobacterales | Campylobacteraceae | Arcobacter | Arcobacter_cibarius |
| Otu03172 | Actinobacteria | Actinobacteria | Actinomycetales | Micromonosporaceae | Dactylosporangium | Dactylosporangium_vinaceum |
| Otu03230 | Bacteroidetes | Flavobacteriia | Flavobacteriales | Flavobacteriaceae | Flavobacterium | Flavobacterium_dankookense |
| Otu03229 | Bacteroidetes | Flavobacteriia | Flavobacteriales | Flavobacteriaceae | Flavobacterium | Flavobacterium_unclassified |
| Otu03217 | Gemmatimonadetes | Gemmatimonadetes | Gemmatimonadales | Gemmatimonadaceae | Gemmatimonas | Gemmatimonas_unclassified |
| Otu03209 | Bacteroidetes | Sphingobacteriia | Sphingobacteriales | Sphingobacteriaceae | Mucilaginibacter | Mucilaginibacter_unclassified |
| Otu03210 | Proteobacteria | Oligoflexia | Oligoflexales | Oligoflexaceae | Oligoflexus | Oligoflexus_unclassified |
| Otu03206 | Proteobacteria | Betaproteobacteria | Burkholderiales | Burkholderiaceae | Paucimonas | Paucimonas_unclassified |
| Otu03174 | Spirochaetes | Spirochaetia | Spirochaetales | Spirochaetaceae | Salinispira | Salinispira_unclassified |
| Otu03199 | Proteobacteria | Betaproteobacteria | Burkholderiales | Burkholderiales_incertae_sedis | Sphaerotilus | Sphaerotilus_montanus |
| Otu03198 | Spirochaetes | Spirochaetia | Spirochaetales | Leptospiraceae | Turneriella | Turneriella_parva |
| Otu03177 | Proteobacteria | Alphaproteobacteria | Rhizobiales | Rhizobiales_incertae_sedis | Vasilyevaea | Vasilyevaea_enhydra |
| Otu03270 | Proteobacteria | Betaproteobacteria | Burkholderiales | Oxalobacteraceae | Duganella | Duganella_phyllosphaerae |
| Otu03253 | Proteobacteria | Betaproteobacteria | Burkholderiales | Oxalobacteraceae | Janthinobacterium | Janthinobacterium_unclassified |
| Otu03245 | Bacteroidetes | Sphingobacteriia | Sphingobacteriales | Sphingobacteriaceae | Pedobacter | Pedobacter_alluvionis |
| Otu03364 | Bacteroidetes | Cytophagia | Cytophagales | Cytophagaceae | Dyadobacter | Dyadobacter_hamtensis |
| Otu03343 | Actinobacteria | Actinobacteria | Actinomycetales | Micromonosporaceae | Luedemannella | Luedemannella_unclassified |
| Otu03323 | Proteobacteria | Gammaproteobacteria | Methylococcales | Methylococcaceae | Methylococcus | Methylococcus_unclassified |
| Otu03336 | Candidatus Saccharibacteria | Saccharibacteria_genera_incertae_sedis | Saccharibacteria_genera_incertae_sedis | Saccharibacteria_genera_incertae_sedis | Saccharibacteria_genera_incertae_sedis | Saccharibacteria_genera_incertae_sedis_unclassified |
| Otu03363 | Bacteroidetes | Cytophagia | Cytophagales | Cytophagaceae | Spirosoma | Spirosoma_spitsbergense |
| Otu03424 | Firmicutes | Clostridia | Clostridiales | Ruminococcaceae | Acetanaerobacterium | Acetanaerobacterium_unclassified |
| Otu03410 | Firmicutes | Clostridia | Clostridiales | Lachnospiraceae | Blautia | Blautia_wexlerae |
| Otu03388 | Bacteroidetes | Flavobacteriia | Flavobacteriales | Flavobacteriaceae | Flavobacterium | Flavobacterium_plurextorum |
| Otu03437 | Proteobacteria | Betaproteobacteria | Burkholderiales | Burkholderiales_incertae_sedis | Ideonella | Ideonella_sakaiensis |
| Otu03369 | Firmicutes | Bacilli | Lactobacillales | Carnobacteriaceae | Jeotgalibaca | Jeotgalibaca_unclassified |
| Otu03440 | Actinobacteria | Actinobacteria | Actinomycetales | Nocardioidaceae | Nocardioides | Nocardioides_conyzicola |
| Otu03472 | Bacteroidetes | Flavobacteriia | Flavobacteriales | Flavobacteriaceae | Flavobacterium | Flavobacterium_unclassified |
| Otu03491 | Acidobacteria | Acidobacteria_Gp16 | Gp16 | Gp16 | Gp16 | Gp16_unclassified |
| Otu03525 | Acidobacteria | Acidobacteria_Gp6 | Gp6 | Gp6 | Gp6 | Gp6_unclassified |
| Otu03534 | Firmicutes | Clostridia | Clostridiales | Ruminococcaceae | Ruminococcus | Ruminococcus_unclassified |
| Otu03596 | Proteobacteria | Gammaproteobacteria | Pseudomonadales | Moraxellaceae | Acinetobacter | Acinetobacter_junii |
| Otu03577 | Firmicutes | Clostridia | Clostridiales | Lachnospiraceae | Blautia | Blautia_unclassified |
| Otu03566 | Bacteroidetes | Cytophagia | Cytophagales | Flammeovirgaceae | Fabibacter | Fabibacter_unclassified |
| Otu03580 | Bacteroidetes | Sphingobacteriia | Sphingobacteriales | Sphingobacteriaceae | Pedobacter | Pedobacter_unclassified |
| Otu03602 | Firmicutes | Negativicutes | Selenomonadales | Veillonellaceae | Pelosinus | Pelosinus_unclassified |
| Otu03570 | Firmicutes | Clostridia | Clostridiales | Clostridiaceae 1 | Proteiniclasticum | Proteiniclasticum_unclassified |
| Otu03541 | Proteobacteria | Betaproteobacteria | Burkholderiales | Comamonadaceae | Rhodoferax | Rhodoferax_ferrireducens |
| Otu03563 | Actinobacteria | Actinobacteria | Actinomycetales | Microbacteriaceae | Salinibacterium | Salinibacterium_unclassified |
| Otu03599 | Proteobacteria | Alphaproteobacteria | Sphingomonadales | Sphingomonadaceae | Sphingopyxis | Sphingopyxis_unclassified |
| Otu03565 | Bacteroidetes | Sphingobacteriia | Sphingobacteriales | Chitinophagaceae | Taibaiella | Taibaiella_unclassified |
| Otu03638 | Firmicutes | Clostridia | Clostridiales | Ruminococcaceae | Anaerotruncus | Anaerotruncus_unclassified |
| Otu03680 | Bacteroidetes | Flavobacteriia | Flavobacteriales | Flavobacteriaceae | Flavobacterium | Flavobacterium_unclassified |
| Otu03688 | Fusobacteria | Fusobacteriia | Fusobacteriales | Fusobacteriaceae | Fusobacterium | Fusobacterium_unclassified |
| Otu03675 | Bacteroidetes | Flavobacteriia | Flavobacteriales | Flavobacteriaceae | Gelidibacter | Gelidibacter_unclassified |
| Otu03674 | Bacteroidetes | Sphingobacteriia | Sphingobacteriales | Saprospiraceae | Haliscomenobacter | Haliscomenobacter_unclassified |
| Otu03708 | Proteobacteria | Deltaproteobacteria | Desulfuromonadales | Desulfuromonadaceae | Malonomonas | Malonomonas_unclassified |
| Otu03670 | Proteobacteria | Gammaproteobacteria | Oceanospirillales | Oceanospirillaceae | Marinospirillum | Marinospirillum_unclassified |
| Otu03690 | Actinobacteria | Actinobacteria | Actinomycetales | Nocardioidaceae | Nocardioides | Nocardioides_gilvus |
| Otu03684 | Proteobacteria | Alphaproteobacteria | Sphingomonadales | Sphingomonadaceae | Novosphingobium | Novosphingobium_unclassified |
| Otu03695 | Proteobacteria | Alphaproteobacteria | Caulobacterales | Caulobacteraceae | Phenylobacterium | Phenylobacterium_unclassified |
| Otu03615 | Bacteroidetes | Bacteroidia | Bacteroidales | Porphyromonadaceae | Proteiniphilum | Proteiniphilum_unclassified |
| Otu03627 | Spirochaetes | Spirochaetia | Spirochaetales | Spirochaetaceae | Salinispira | Salinispira_unclassified |
| Otu03782 | Actinobacteria | Actinobacteria | Acidimicrobiales | Iamiaceae | Aquihabitans | Aquihabitans_unclassified |
| Otu03748 | Bacteroidetes | Flavobacteriia | Flavobacteriales | Flavobacteriaceae | Flavobacterium | Flavobacterium_algicola |
| Otu03755 | Bacteroidetes | Sphingobacteriia | Sphingobacteriales | Saprospiraceae | Haliscomenobacter | Haliscomenobacter_unclassified |
| Otu03794 | Chloroflexi | Chloroflexia | Kallotenuales | Kallotenuaceae | Kallotenue | Kallotenue_unclassified |
| Otu03769 | Proteobacteria | Deltaproteobacteria | Myxococcales | Polyangiaceae | Minicystis | Minicystis_unclassified |
| Otu03743 | Actinobacteria | Actinobacteria | Actinomycetales | Nakamurellaceae | Nakamurella | Nakamurella_unclassified |
| Otu03721 | Proteobacteria | Alphaproteobacteria | Rhodobacterales | Rhodobacteraceae | Roseicyclus | Roseicyclus_unclassified |
| Otu03716 | Proteobacteria | Gammaproteobacteria | Alteromonadales | Shewanellaceae | Shewanella | Shewanella_profunda |
| Otu03899 | Proteobacteria | Alphaproteobacteria | Sphingomonadales | Erythrobacteraceae | Altererythrobacter | Altererythrobacter_palmitatis |
| Otu03934 | Bacteroidetes | Bacteroidia | Bacteroidales | Rikenellaceae | Anaerocella | Anaerocella_unclassified |
| Otu03905 | Armatimonadetes | Armatimonadia | Armatimonadales | Armatimonadaceae | Armatimonas/Armatimonadetes_gp1 | Armatimonas/Armatimonadetes_gp1_unclassified |
| Otu03916 | Firmicutes | Bacilli | Lactobacillales | Carnobacteriaceae | Atopostipes | Atopostipes_unclassified |
| Otu03917 | Proteobacteria | Alphaproteobacteria | Rhodobacterales | Rhodobacteraceae | Gemmobacter | Gemmobacter_unclassified |
| Otu03825 | Actinobacteria | Actinobacteria | Actinomycetales | Microbacteriaceae | Herbiconiux | Herbiconiux_unclassified |
| Otu03891 | Proteobacteria | Deltaproteobacteria | Myxococcales | Polyangiaceae | Minicystis | Minicystis_unclassified |
| Otu03902 | Bacteroidetes | Sphingobacteriia | Sphingobacteriales | Sphingobacteriaceae | Mucilaginibacter | Mucilaginibacter_unclassified |
| Otu03820 | Bacteroidetes | Bacteroidia | Bacteroidales | Porphyromonadaceae | Paludibacter | Paludibacter_unclassified |
| Otu03896 | Bacteroidetes | Sphingobacteriia | Sphingobacteriales | Sphingobacteriaceae | Parapedobacter | Parapedobacter_unclassified |
| Otu03894 | Proteobacteria | Betaproteobacteria | Burkholderiales | Comamonadaceae | Polaromonas | Polaromonas_cryoconiti |
| Otu03892 | Proteobacteria | Alphaproteobacteria | Rhizobiales | Rhizobiaceae | Rhizobium | Rhizobium_cauense |
| Otu03851 | Firmicutes | Clostridia | Clostridiales | Clostridiales_Incertae Sedis XI | Tissierella | Tissierella_unclassified |
| Otu04023 | Acidobacteria | Acidobacteria_Gp4 | Aridibacter | Aridibacter | Aridibacter | Aridibacter_nitratireducens |
| Otu04043 | Bacteroidetes | Sphingobacteriia | Sphingobacteriales | Sphingobacteriaceae | Pedobacter | Pedobacter_terrae |
| Otu04029 | Firmicutes | Negativicutes | Selenomonadales | Veillonellaceae | Pelosinus | Pelosinus_unclassified |
| Otu03937 | Actinobacteria | Actinobacteria | Solirubrobacterales | Solirubrobacteraceae | Solirubrobacter | Solirubrobacter_unclassified |
| Otu04007 | Proteobacteria | Alphaproteobacteria | Sphingomonadales | Sphingomonadaceae | Sphingopyxis | Sphingopyxis_witflariensis |
| Otu03947 | Proteobacteria | Betaproteobacteria | Rhodocyclales | Rhodocyclaceae | Sulfuritalea | Sulfuritalea_unclassified |
| Otu04049 | Firmicutes | Clostridia | Clostridiales | Clostridiales_Incertae Sedis XI | Tissierella | Tissierella_unclassified |
| Otu04097 | Bacteroidetes | Cytophagia | Cytophagales | Cytophagaceae | Lacihabitans | Lacihabitans_unclassified |
| Otu04095 | Verrucomicrobia | Verrucomicrobiae | Verrucomicrobiales | Verrucomicrobiaceae | Prosthecobacter | Prosthecobacter_unclassified |
| Otu04119 | Proteobacteria | Gammaproteobacteria | Xanthomonadales | Xanthomonadaceae | Pseudoxanthomonas | Pseudoxanthomonas_spadix |
| Otu04066 | Firmicutes | Bacilli | Bacillales | Staphylococcaceae | Staphylococcus | Staphylococcus_vitulinus |
| Otu04281 | Proteobacteria | Betaproteobacteria | Neisseriales | Neisseriaceae | Aquaspirillum | Aquaspirillum_putridiconchylium |
| Otu04319 | Chloroflexi | Ardenticatenia | Ardenticatenales | Ardenticatenaceae | Ardenticatena | Ardenticatena_unclassified |
| Otu04253 | Cyanobacteria/Chloroplast | Chloroplast | Chloroplast | Chloroplast | Chlorarachniophyceae | Chlorarachniophyceae_unclassified |
| Otu04214 | Bacteroidetes | Sphingobacteriia | Sphingobacteriales | Chitinophagaceae | Cnuella | Cnuella_unclassified |
| Otu04235 | Bacteroidetes | Flavobacteriia | Flavobacteriales | Flavobacteriaceae | Flavobacterium | Flavobacterium_unclassified |
| Otu04268 | Actinobacteria | Actinobacteria | Acidimicrobiales | Acidimicrobiaceae | Ilumatobacter | Ilumatobacter_unclassified |
| Otu04256 | Nitrospirae | Nitrospira | Nitrospirales | Nitrospiraceae | Nitrospira | Nitrospira_unclassified |
| Otu04227 | Bacteroidetes | Sphingobacteriia | Sphingobacteriales | Sphingobacteriaceae | Pedobacter | Pedobacter_unclassified |
| Otu04237 | Proteobacteria | Deltaproteobacteria | Myxococcales | Nannocystaceae | Plesiocystis | Plesiocystis_unclassified |
| Otu04322 | Bacteroidetes | Sphingobacteriia | Sphingobacteriales | Saprospiraceae | Portibacter | Portibacter_unclassified |
| Otu04184 | Spirochaetes | Spirochaetia | Spirochaetales | Spirochaetaceae | Salinispira | Salinispira_unclassified |
| Otu04230 | Lentisphaerae | Lentisphaeria | Victivallales | Victivallaceae | Victivallis | Victivallis_unclassified |
| Otu04345 | Bacteroidetes | Cytophagia | Cytophagales | Cytophagaceae | Arcicella | Arcicella_unclassified |
| Otu04457 | Bacteroidetes | Bacteroidia | Bacteroidales | Porphyromonadaceae | Paludibacter | Paludibacter_unclassified |
| Otu04422 | Actinobacteria | Actinobacteria | Actinomycetales | Micromonosporaceae | Phytohabitans | Phytohabitans_unclassified |
| Otu04328 | Bacteroidetes | Sphingobacteriia | Sphingobacteriales | Saprospiraceae | Portibacter | Portibacter_unclassified |
| Otu04385 | Proteobacteria | Gammaproteobacteria | Pseudomonadales | Pseudomonadaceae | Rhizobacter | Rhizobacter_bergeniae |
| Otu04392 | Proteobacteria | Alphaproteobacteria | Rhizobiales | Rhizobiales_incertae_sedis | Vasilyevaea | Vasilyevaea_enhydra |
| Otu04559 | Proteobacteria | Deltaproteobacteria | Myxococcales | Polyangiaceae | Byssovorax | Byssovorax_unclassified |
| Otu04620 | Firmicutes | Clostridia | Clostridiales | Lachnospiraceae | Catonella | Catonella_unclassified |
| Otu04496 | Bacteroidetes | Sphingobacteriia | Sphingobacteriales | Saprospiraceae | Haliscomenobacter | Haliscomenobacter_unclassified |
| Otu04571 | Actinobacteria | Actinobacteria | Acidimicrobiales | Acidimicrobiaceae | Ilumatobacter | Ilumatobacter_unclassified |
| Otu04579 | Proteobacteria | Betaproteobacteria | Burkholderiales | Oxalobacteraceae | Oxalobacter | Oxalobacter_unclassified |
| Otu04599 | Bacteroidetes | Sphingobacteriia | Sphingobacteriales | Saprospiraceae | Phaeodactylibacter | Phaeodactylibacter_unclassified |
| Otu04701 | Actinobacteria | Actinobacteria | Acidimicrobiales | Iamiaceae | Aquihabitans | Aquihabitans_unclassified |
| Otu04757 | Cyanobacteria/Chloroplast | Chloroplast | Chloroplast | Chloroplast | Chlorophyta | Chlorophyta_unclassified |
| Otu04758 | Cyanobacteria/Chloroplast | Chloroplast | Chloroplast | Chloroplast | Chlorophyta | Chlorophyta_unclassified |
| Otu04651 | Bacteroidetes | Sphingobacteriia | Sphingobacteriales | Chitinophagaceae | Ferruginibacter | Ferruginibacter_alkalilentus |
| Otu04734 | Bacteroidetes | Flavobacteriia | Flavobacteriales | Flavobacteriaceae | Flavobacterium | Flavobacterium_unclassified |
| Otu04646 | Proteobacteria | Gammaproteobacteria | Legionellales | Legionellaceae | Legionella | Legionella_unclassified |
| Otu04759 | Proteobacteria | Alphaproteobacteria | Sphingomonadales | Sphingomonadaceae | Novosphingobium | Novosphingobium_rosa |
| Otu04756 | Firmicutes | Bacilli | Bacillales | Paenibacillaceae 1 | Paenibacillus | Paenibacillus_glacialis |
| Otu04726 | Proteobacteria | Betaproteobacteria | Burkholderiales | Comamonadaceae | Rhodoferax | Rhodoferax_ferrireducens |
| Otu04719 | Proteobacteria | Alphaproteobacteria | Sphingomonadales | Sphingomonadaceae | Sphingomonas | Sphingomonas_soli |
| Otu04864 | Bacteroidetes | Bacteroidia | Bacteroidales | Bacteroidaceae | Anaerorhabdus | Anaerorhabdus_unclassified |
| Otu04832 | Acidobacteria | Acidobacteria_Gp3 | Gp3 | Gp3 | Gp3 | Gp3_unclassified |
| Otu04948 | Proteobacteria | Alphaproteobacteria | Rhodobacterales | Rhodobacteraceae | Rhodobacter | Rhodobacter_unclassified |
| Otu05002 | Proteobacteria | Alphaproteobacteria | Rhodospirillales | Acetobacteraceae | Roseomonas | Roseomonas_unclassified |
| Otu05176 | Proteobacteria | Betaproteobacteria | Rhodocyclales | Rhodocyclaceae | Dechloromonas | Dechloromonas_denitrificans |
| Otu05161 | Proteobacteria | Betaproteobacteria | Rhodocyclales | Rhodocyclaceae | Propionivibrio | Propionivibrio_unclassified |
| Otu05103 | Proteobacteria | Deltaproteobacteria | Myxococcales | Sandaracinaceae | Sandaracinus | Sandaracinus_unclassified |
| Otu05156 | Proteobacteria | Deltaproteobacteria | Myxococcales | Polyangiaceae | Sorangium | Sorangium_unclassified |
| Otu05198 | Spirochaetes | Spirochaetia | Spirochaetales | Spirochaetaceae | Treponema | Treponema_unclassified |
| Otu05307 | Proteobacteria | Gammaproteobacteria | Pseudomonadales | Moraxellaceae | Alkanindiges | Alkanindiges_unclassified |
| Otu05287 | Bacteroidetes | Sphingobacteriia | Sphingobacteriales | Chitinophagaceae | Flavihumibacter | Flavihumibacter_unclassified |
| Otu05365 | Bacteroidetes | Sphingobacteriia | Sphingobacteriales | Chitinophagaceae | Flavitalea | Flavitalea_unclassified |
| Otu05460 | Bacteroidetes | Sphingobacteriia | Sphingobacteriales | Chitinophagaceae | Taibaiella | Taibaiella_unclassified |
| Otu05733 | Proteobacteria | Gammaproteobacteria | Pseudomonadales | Moraxellaceae | Acinetobacter | Acinetobacter_unclassified |
| Otu05570 | Actinobacteria | Actinobacteria | Actinomycetales | Micromonosporaceae | Actinoplanes | Actinoplanes_abujensis |
| Otu05639 | Bacteroidetes | Flavobacteriia | Flavobacteriales | Flavobacteriaceae | Flavobacterium | Flavobacterium_unclassified |
| Otu05535 | Actinobacteria | Actinobacteria | Actinomycetales | Streptomycetaceae | Streptomyces | Streptomyces_globisporus |
| Otu05953 | Proteobacteria | Deltaproteobacteria | Myxococcales | Polyangiaceae | Byssovorax | Byssovorax_unclassified |
| Otu05799 | Bacteroidetes | Bacteroidia | Bacteroidales | Porphyromonadaceae | Dysgonomonas | Dysgonomonas_unclassified |
| Otu05899 | Bacteroidetes | Sphingobacteriia | Sphingobacteriales | Sphingobacteriaceae | Pedobacter | Pedobacter_unclassified |
| Otu06011 | Spirochaetes | Spirochaetia | Spirochaetales | Spirochaetaceae | Salinispira | Salinispira_unclassified |
| Otu06169 | Proteobacteria | Betaproteobacteria | Burkholderiales | Oxalobacteraceae | Janthinobacterium | Janthinobacterium_svalbardensis |
| Otu06118 | Proteobacteria | Betaproteobacteria | Burkholderiales | Comamonadaceae | Pelomonas | Pelomonas_unclassified |
| Otu06615 | Proteobacteria | Alphaproteobacteria | Caulobacterales | Caulobacteraceae | Phenylobacterium | Phenylobacterium_aquaticum |
| Otu06785 | Proteobacteria | Betaproteobacteria | Neisseriales | Neisseriaceae | Rivicola | Rivicola_unclassified |
| Otu06606 | Bacteroidetes | Sphingobacteriia | Sphingobacteriales | Chitinophagaceae | Taibaiella | Taibaiella_unclassified |

**Table 3.** Samples with low read counts not included in analysis.

| **Sample ID** | **Read counts** | **Site** | **Benign/EC** |
| --- | --- | --- | --- |
| A109 | 30 | Vagina | EC |
| A297 | 53 | Vagina | EC |
| A281 | 235 | Vagina | EC |
| A285 | 238 | Vagina | EC |
| A143 | 748 | Vagina | EC |
| A286 | 57 | Cervix | EC |
| A282 | 74 | Cervix | EC |
| A310 | 80 | Cervix | EC |
| A39 | 137 | Cervix | Benign |
| A278 | 138 | Cervix | EC |
| A148 | 191 | Cervix | EC |
| A111 | 468 | Cervix | EC |
| A144 | 496 | Cervix | EC |
| A123 | 511 | Cervix | EC |
| A38 | 636 | Cervix | Benign |
| A315 | 31 | Endometrium | EC |
| A284 | 67 | Endometrium | EC |
| A299 | 67 | Endometrium | EC |
| A283 | 85 | Endometrium | EC |
| A280 | 87 | Endometrium | EC |
| A287 | 88 | Endometrium | EC |
| A316 | 94 | Endometrium | EC |
| A288 | 101 | Endometrium | EC |
| A300 | 121 | Endometrium | EC |
| A113 | 134 | Endometrium | EC |
| A124 | 134 | Endometrium | EC |
| A154 | 134 | Endometrium | EC |
| A320 | 147 | Endometrium | EC |
| A149 | 157 | Endometrium | EC |
| A312 | 169 | Endometrium | EC |
| A125 | 182 | Endometrium | EC |
| E78 | 183 | Endometrium | Benign |
| A262 | 190 | Endometrium | EC |
| A150 | 202 | Endometrium | EC |
| A136 | 238 | Endometrium | EC |
| A135 | 252 | Endometrium | EC |
| A145 | 259 | Endometrium | EC |
| A311 | 266 | Endometrium | EC |
| A112 | 278 | Endometrium | EC |
| A153 | 290 | Endometrium | EC |

**EC: Endometrial cancer**

| **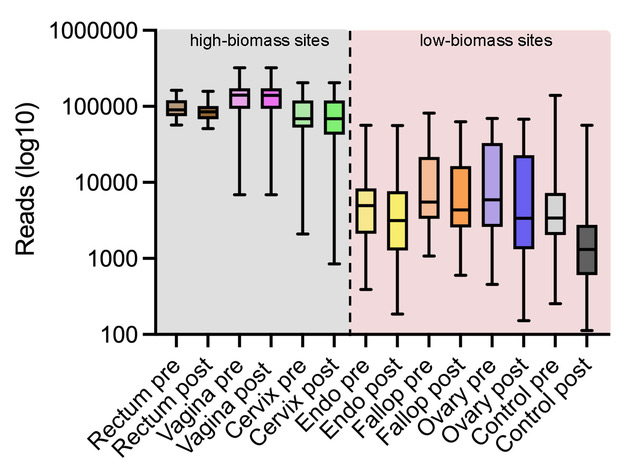**  **Benign** | **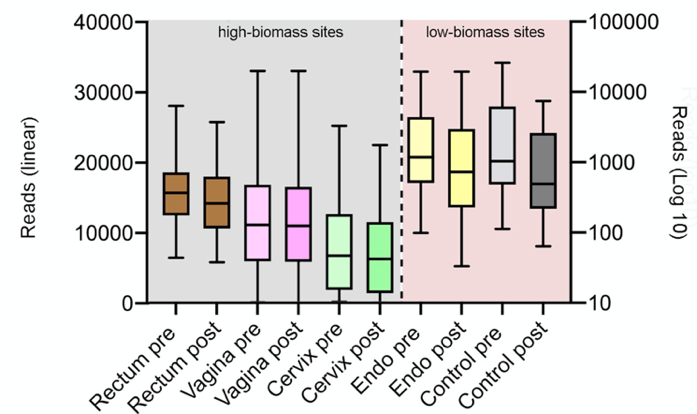**  **Endometrial ca** |
| --- | --- |

**Figure 1.** 16S rRNA gene sequence read counts pre- and post-removal of contaminant sequence reads. Lines represent mean with SD.

Relative abundance

| **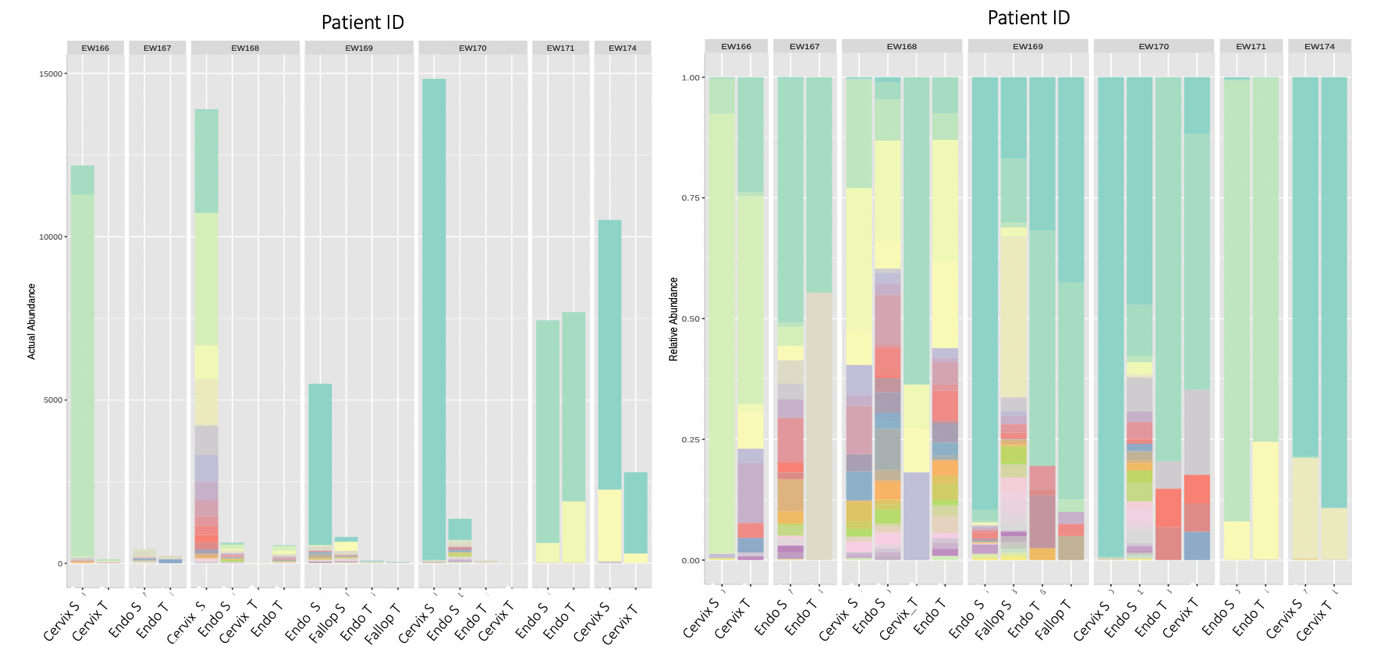**  **A**  Actual abundance | |
| --- | --- |
| **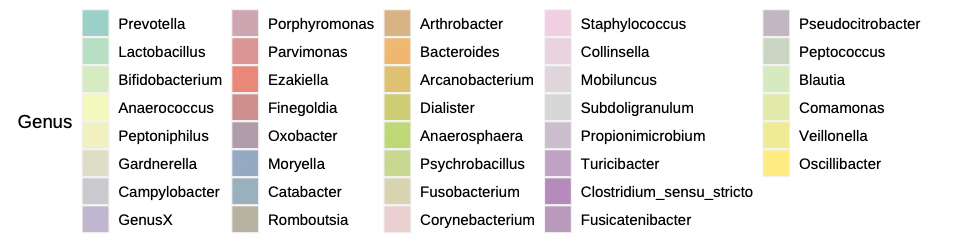** | |
| ****  **B**  p value 1 | ****  p value< 0.109 |

**Figure 2. Swab-tissue pairwise comparison of microbial yield and composition in different anatomic sites of benign and endometrial cancer patients (genera). A.** Tissue actual abundance of microbes is considerably lower than swabs and composition differs in most samples. **B.** Shannon α-diversity and β-diversity did not reveal any significant differences between the two different sampling methods. *S: swab; T: tissue; Endo: Endometrium; Fallop: Fallopian tube.*

**
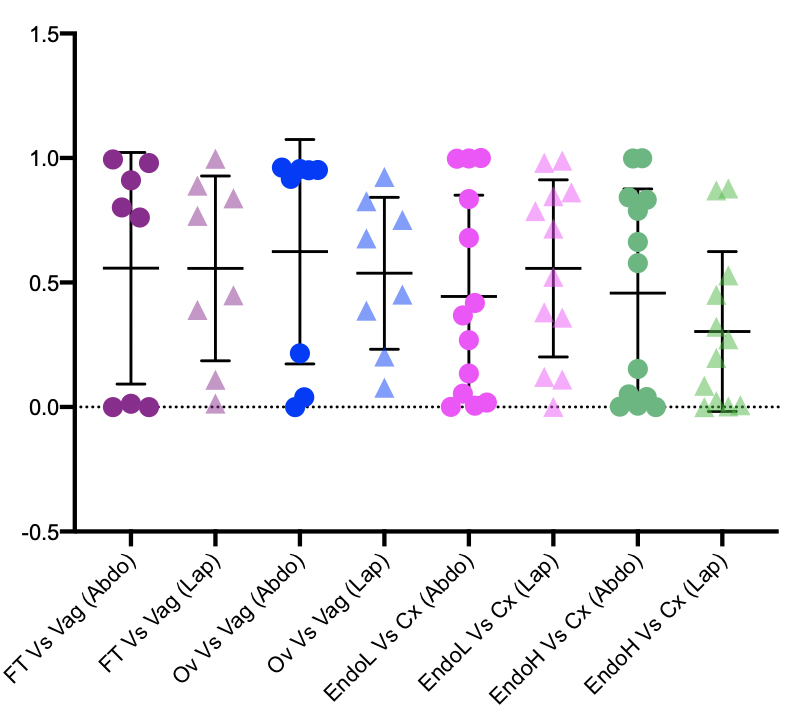
**

Correlation co-efficient (R^2^)

**Figure 3.** **Comparison between laparoscopic and transabdominal procedures to determine potential contamination of low-biomass sites in laparoscopy during transcervical insertion of uterine manipulator and vaginal retrieval of surgical specimen.** Intraindividual calculation of microbial correlation co-efficient (R^2^) at species level between fallopian tubes/ovaries and vagina or lower/higher endometrium and cervix in transabdominal and laparoscopic hysterectomies. Dots and triangles represent individual R^2^ values for each patient. Lines represent mean with SD. *FT: Fallopian tube; Vag: Vagina; Ov: Ovary; EndoL: Endometrium Lower; EndoH: Endometrium Higher; Cx: Cervix; Abdo: Abdominally; Lap: Laparoscopically.*

**
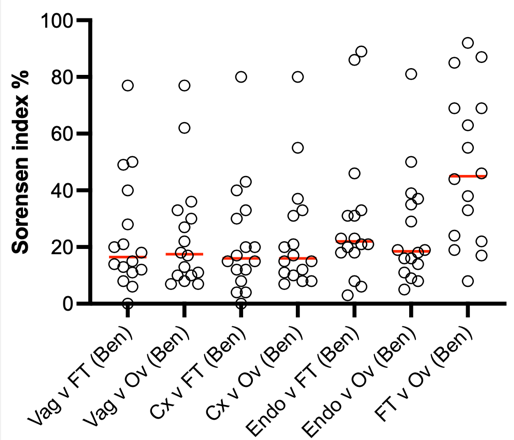
**

**B**

**A**

**C**

n=36


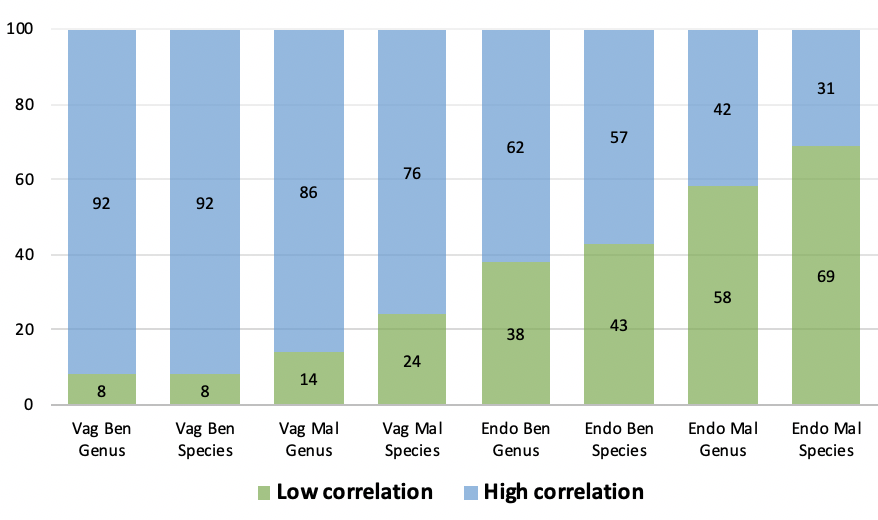


n=13

n=13

n=21

n=21

n=21

n=21

n=36

Correlation co-efficient (R^2^)

| **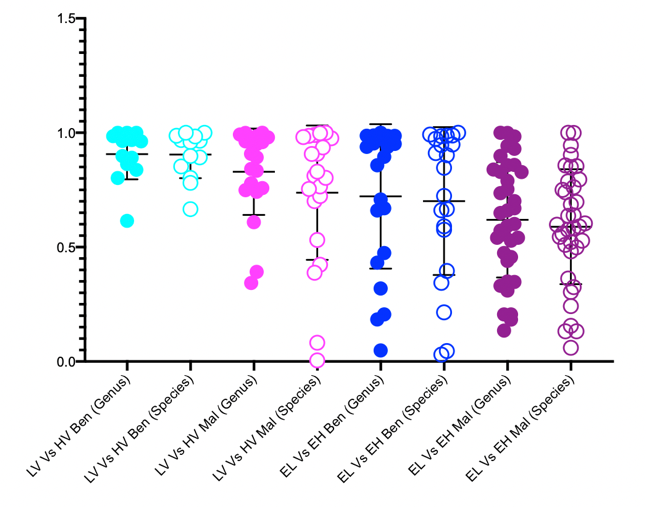**  Percentage (%) |  |
| --- | --- |

**Figure 4. Microbiota continuum in lower and upper female genital tract.** **A.** Similarity analysis at species level between different body sites in benign patients without endometrial malignancy. Dots and circles represent individual values for each patient. Red lines denote median. *Vag: Vagina, FT: Fallopian tube, Ov: Ovary, v: versus*. **B.** Intra-patient correlation of lower versus higher vagina/endometrium at genera and species level in benign and endometrial cancer patients. Lines represent mean with SD. *LV: Lower Vagina; HV: Higher Vagina; EL: Endometrium Lower; EH: Endometrium Higher; Ben: Benign; Mal: Malignant.* **C.** Stacked bar chart illustrating microbial correlation along vaginal or endometrial length in benign and endometrial cancer patients. High correlation defined as R^2^≥ 0.7.

**
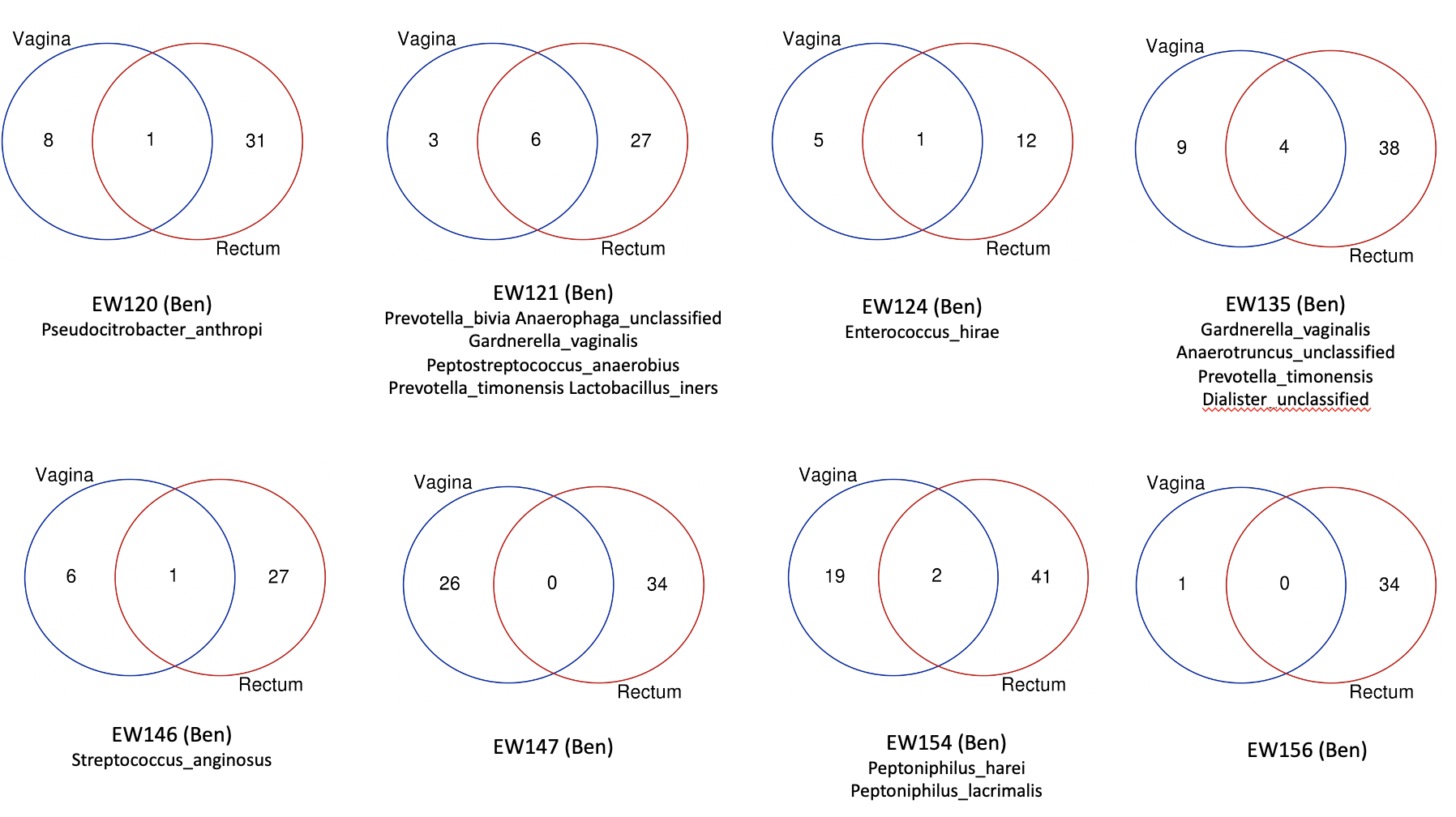
**

**A**

**
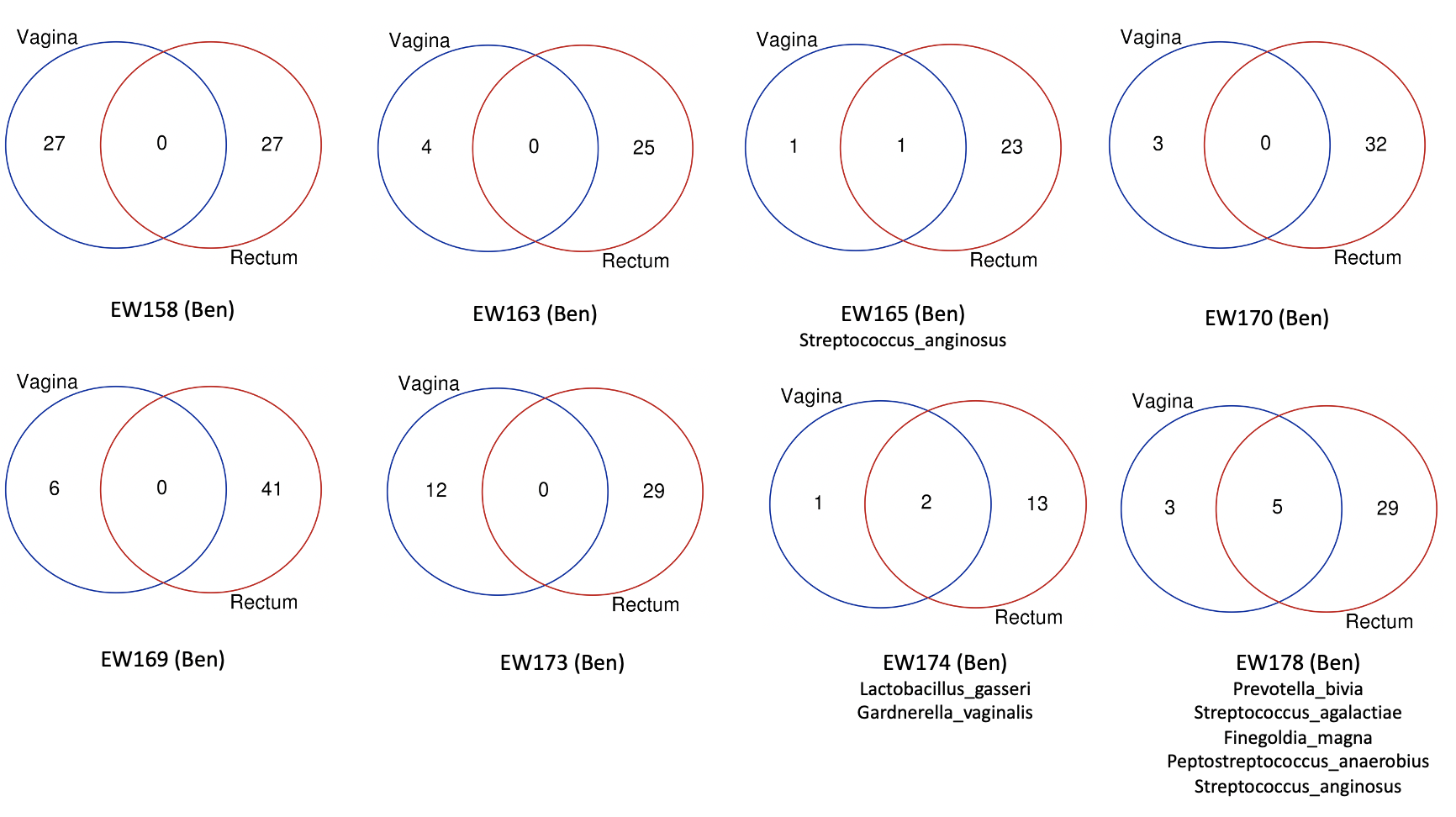
**

**
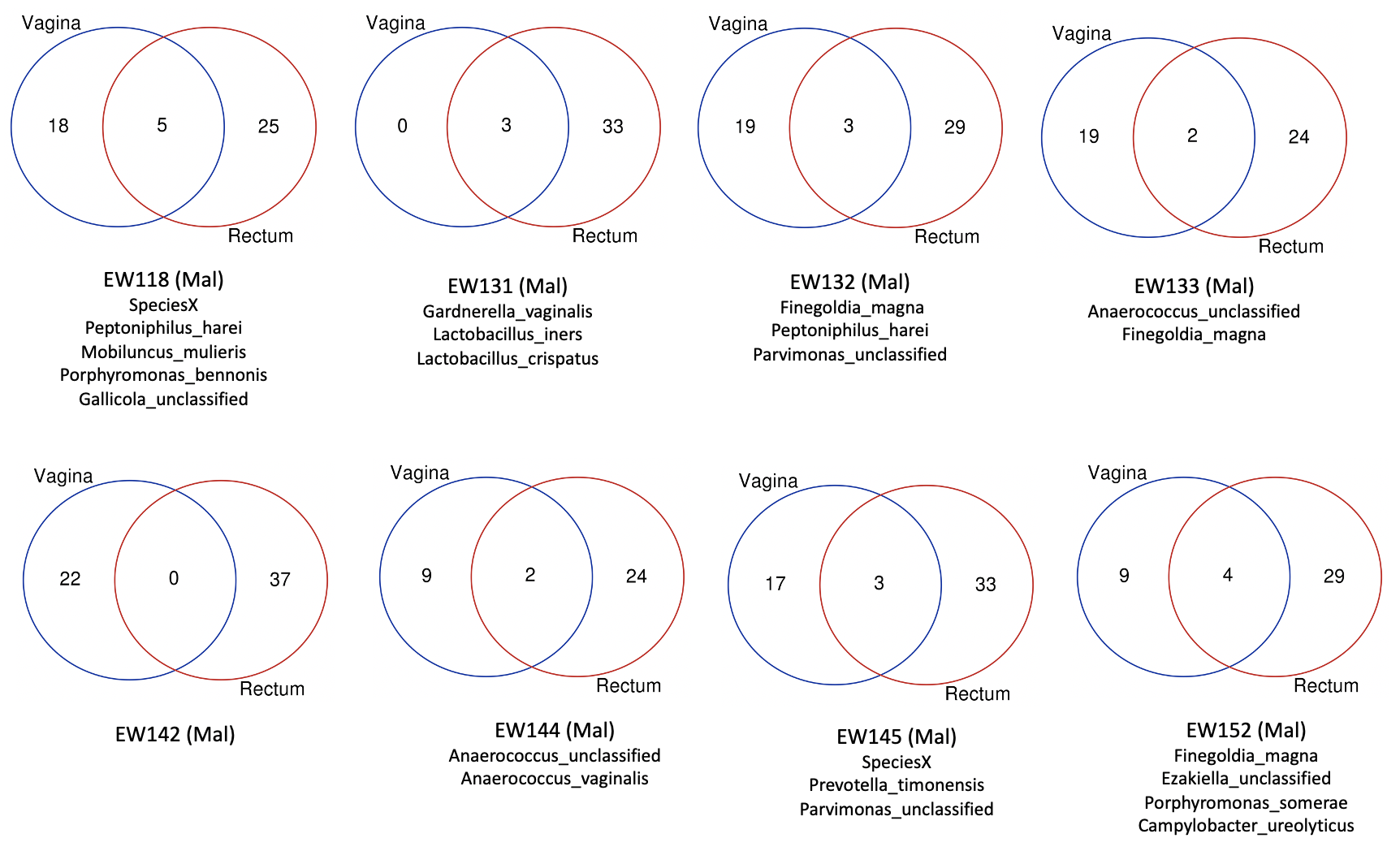
**

**B**

**
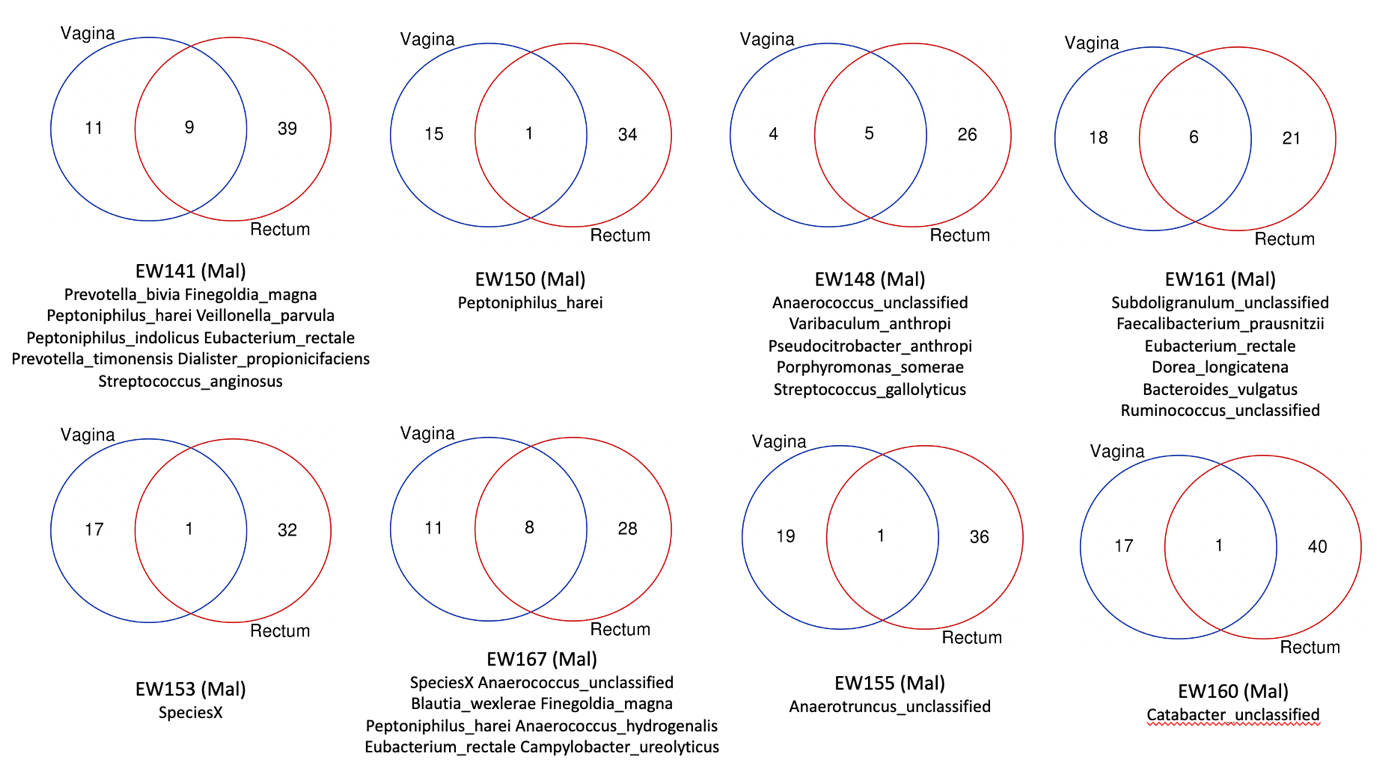
**

**
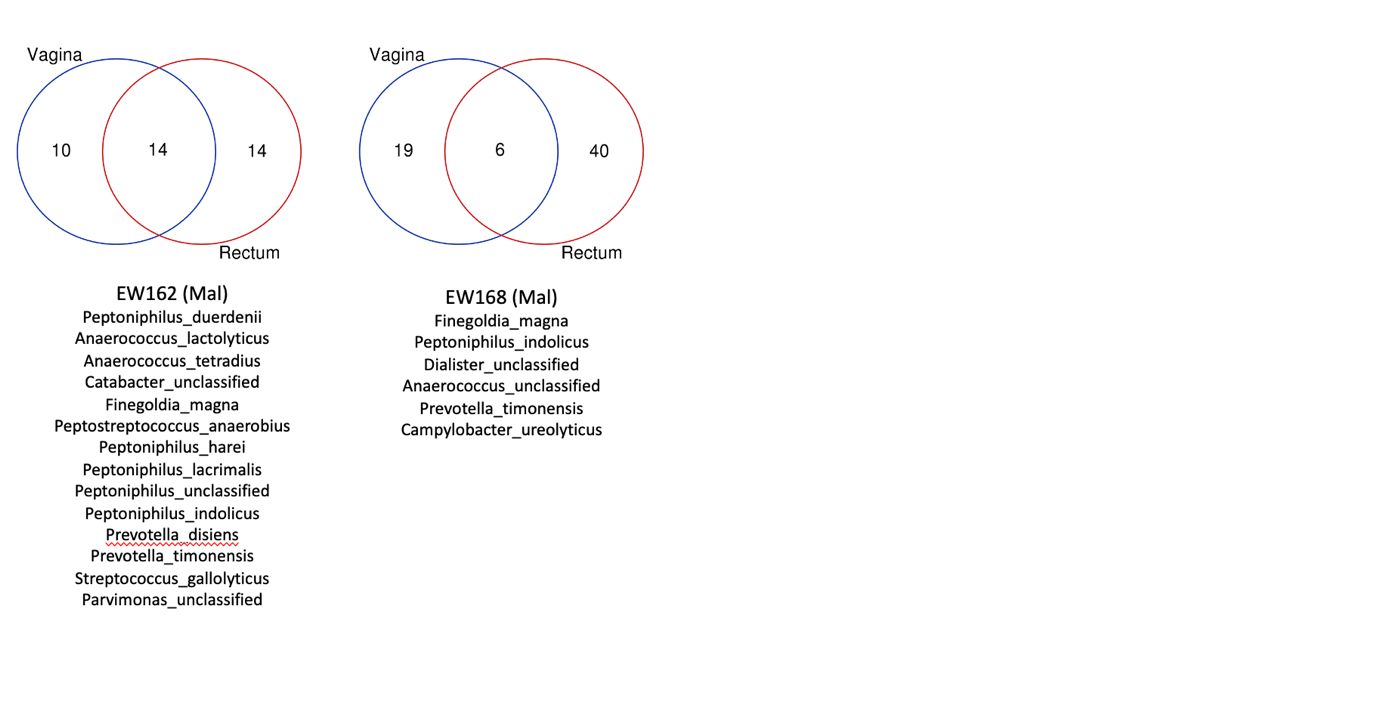

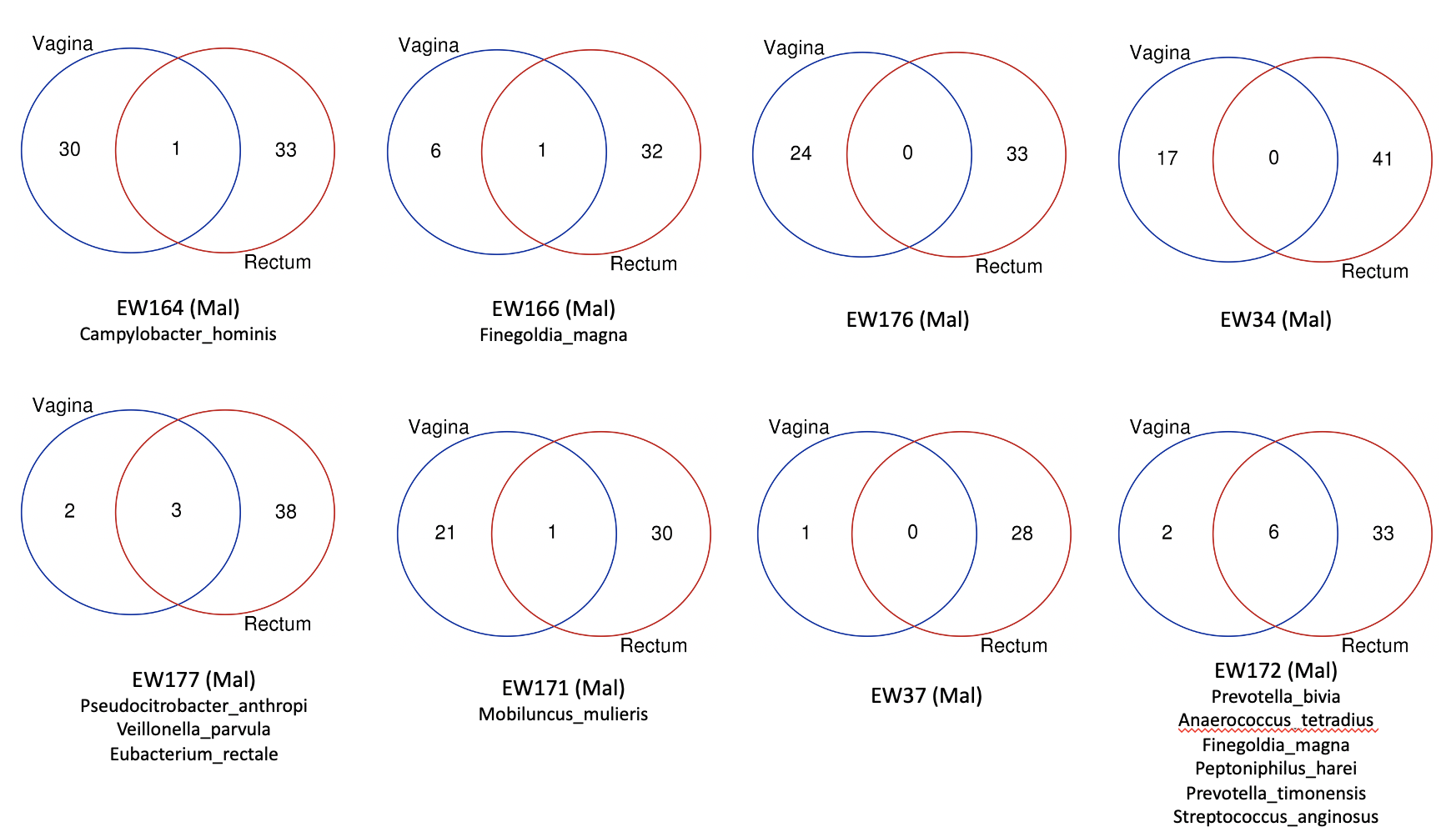
**

**Figure 5. Intra-individual correlation between vaginal-rectal microbiota.** Venn diagrams illustrating microbial species shared by the lower third of vagina and rectum in 16 women with benign conditions and 26 women with endometrial cancer. Only species with an at least 0.5% relative abundance were included. *Ben: Benign; Mal: Malignant.*


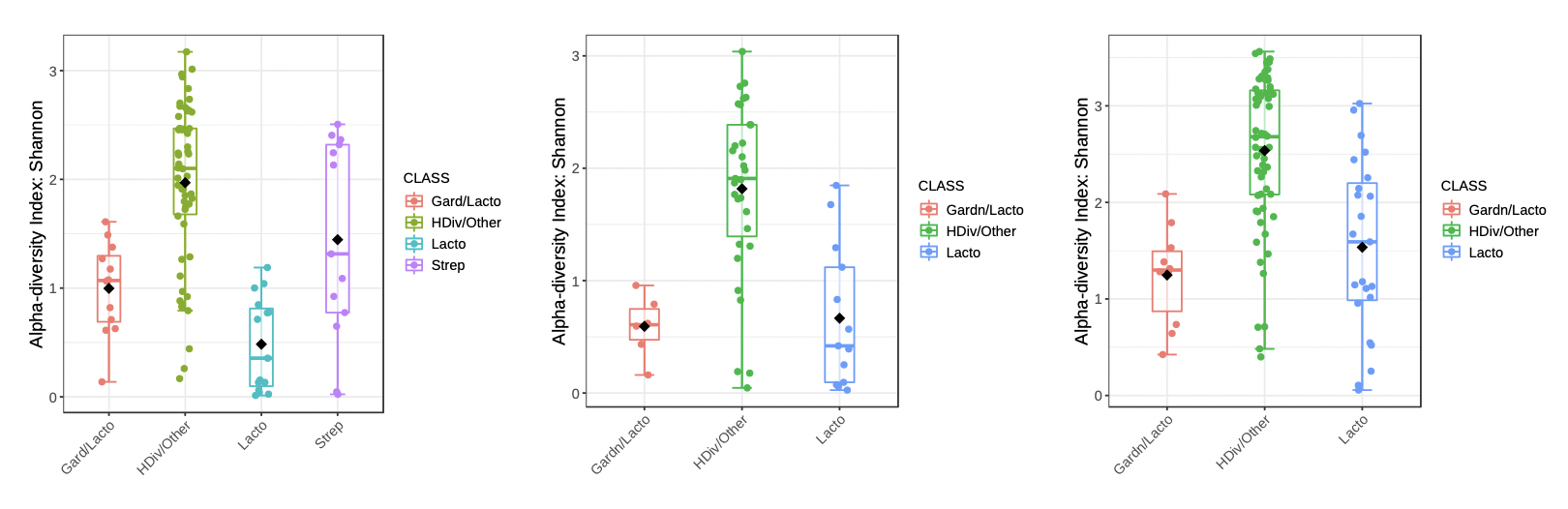


**Cervix**

**Vagina**

**Endometrium**

p-value: 4.4971e-08

p-value: 0.00021524

p-value: 6.0177e-07

**Figure 6. Shannon α-diversity among microbial clusters identified in different anatomical sites (species).** Lacto: *Lactobacillus*; Gardn/Lacto: *Gardnerella/Lactobacillus*; Strep: *Streptococcus*; HDiv/Other: High diversity & Other.

**
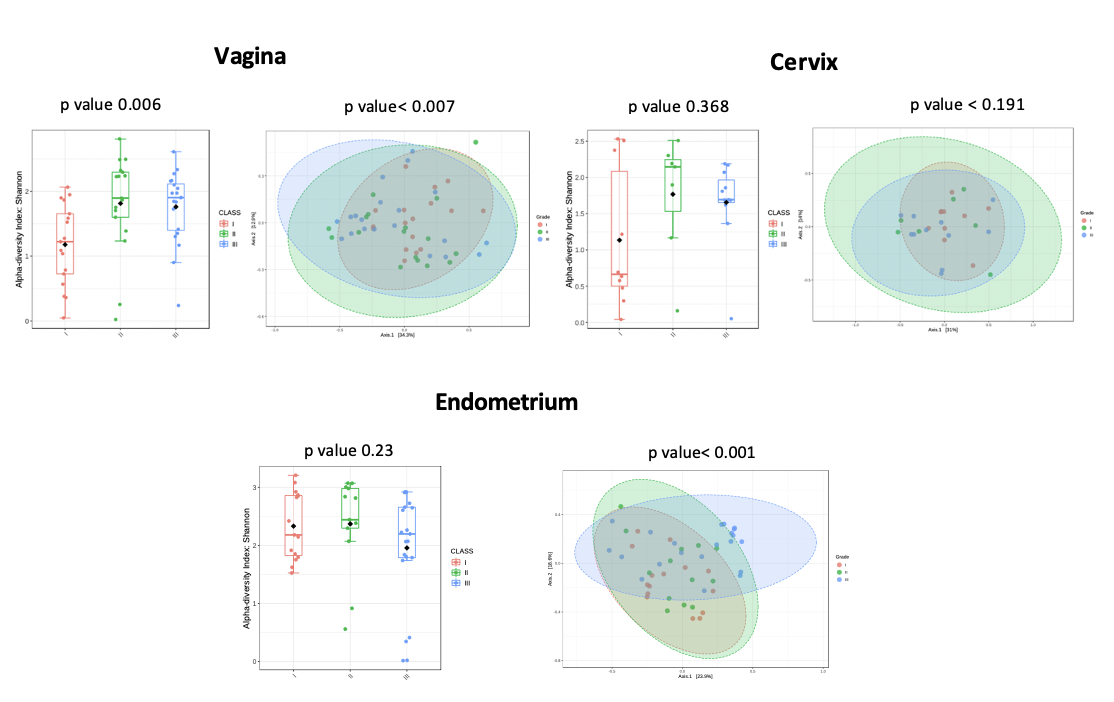

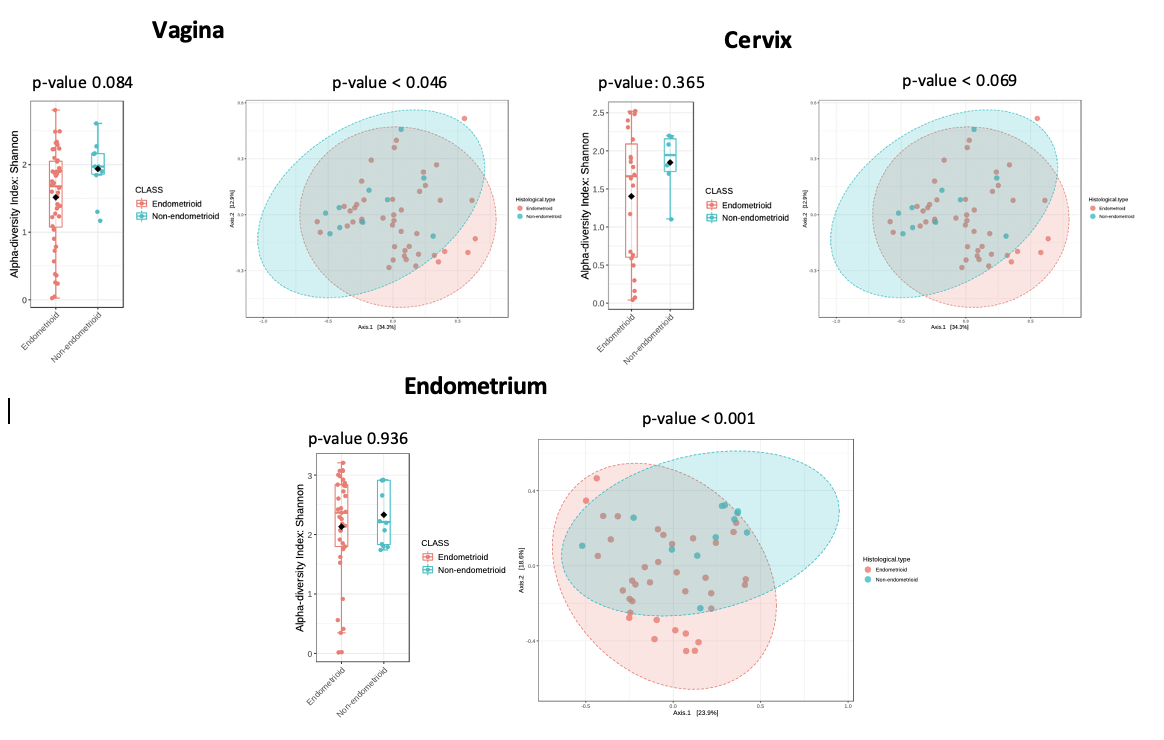
**

**A**

**B**

**Figure 7.** **Microbiome Shannon α- and β-diversity according to histological type and grade of endometrial cancer per anatomical site (genera)**. Microbiome Shannon α- and β-diversity **A.** in endometrioid and non-endometrioid endometrial cancer patients and **B.** in different grades of endometrial cancer.

p value 0.002

p value 0.002

p value 0.007

p value 0.002


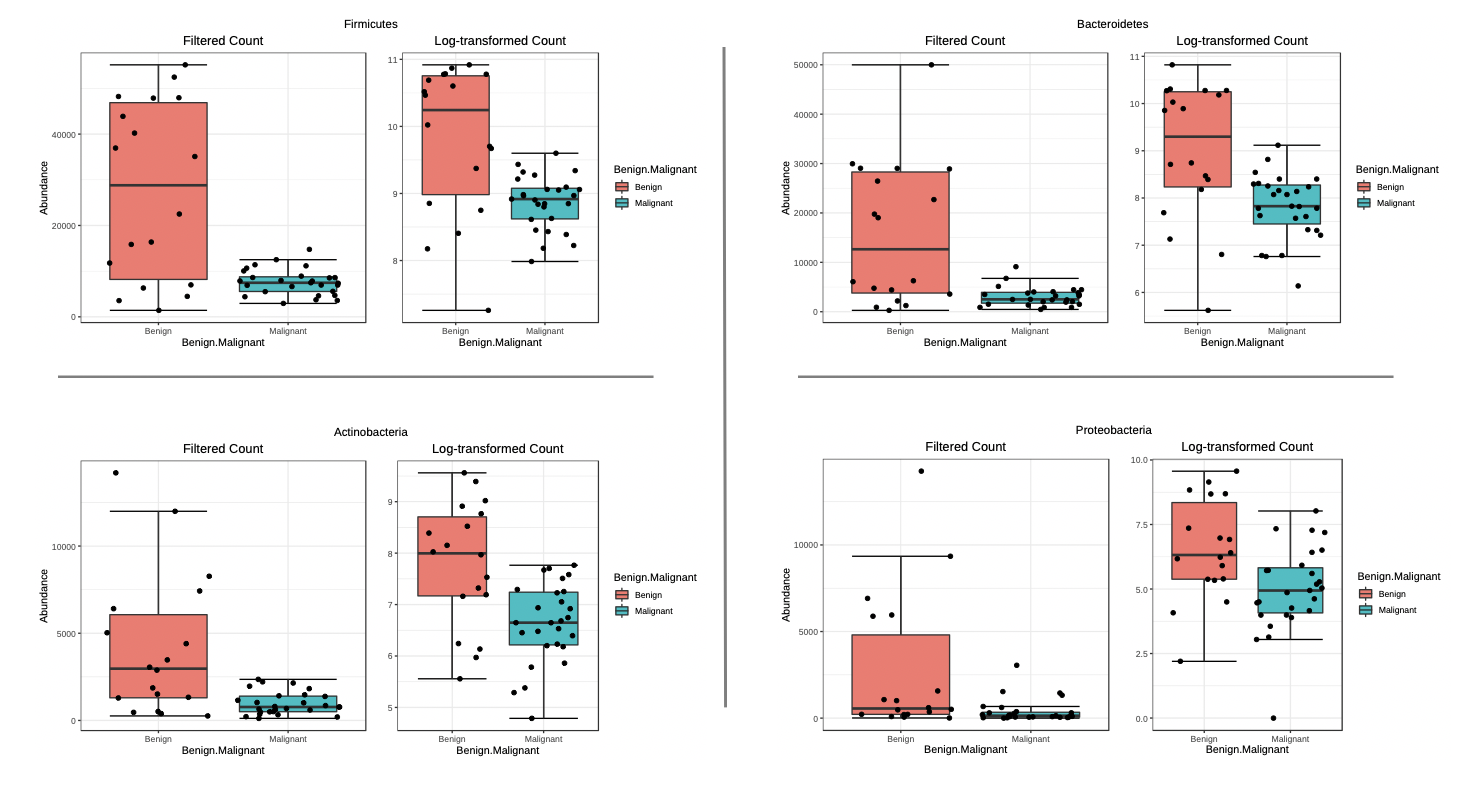


**Firmicutes**

**Bacteroidetes**

**Actinobacteria**

**Proteobacteria**

**A**

**B**

p value < 0.359

p value 0.455

**Figure 8. Comparison of microbial composition in the rectum of women with and without endometrial cancer. A.** The rectum of women with endometrial cancer displays decreased colonisation with *Firmicutes* (*p*= 0.002), *Bacteroidetes* (*p*= 0.002), *Actinobacteria* (*p*= 0.002) and *Proteobacteria* (*p*= 0.007) compared to benign controls. **B.** Microbiome Shannon α- and β- diversity in rectum shows no difference at genera level between benign and endometrial cancer patients.

**Table 4.** Recruits for organoid experiments

**
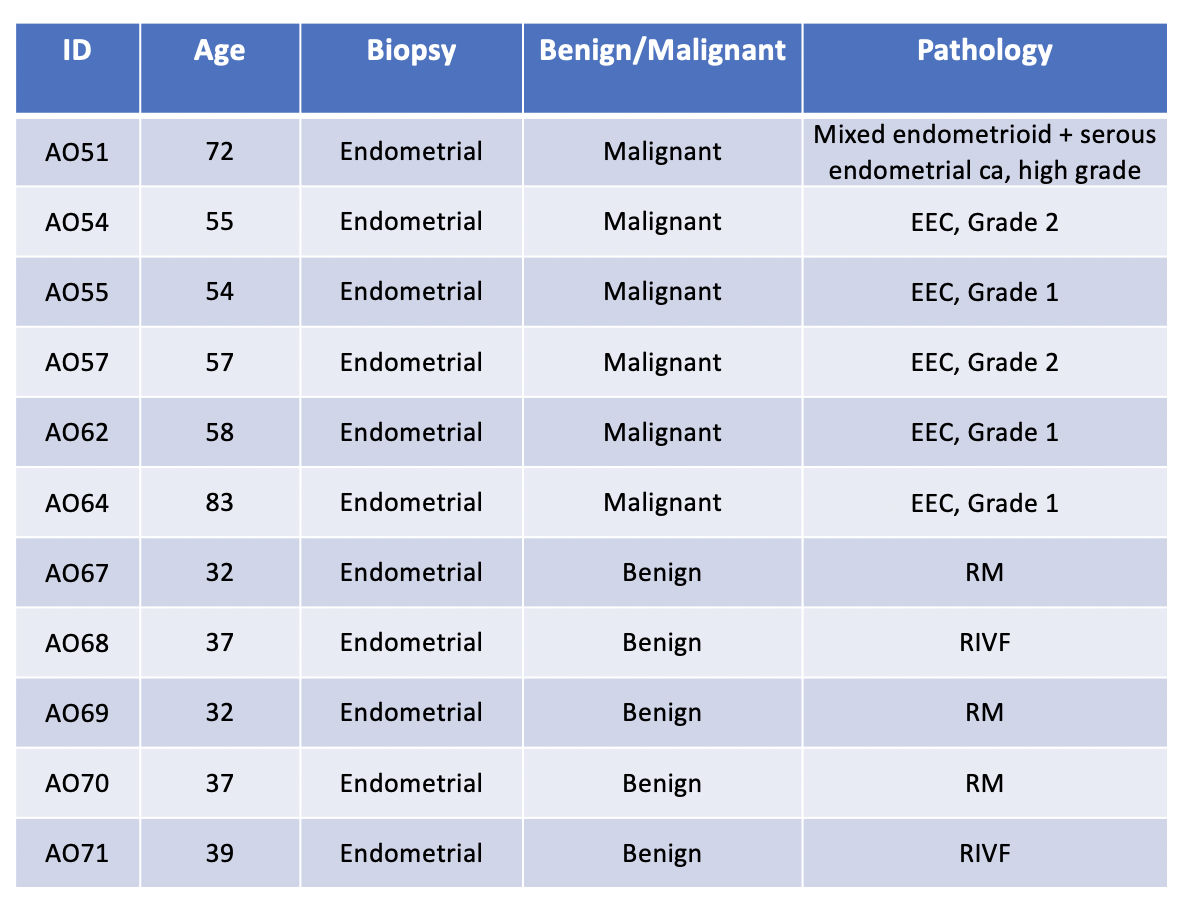
**

***EEC:*** *Endometrioid endometrial cancer,* ***RM:*** *Recurrent miscarriage,* ***RIVF:*** *Recurrent In Vitro Fertilisation failure*

**
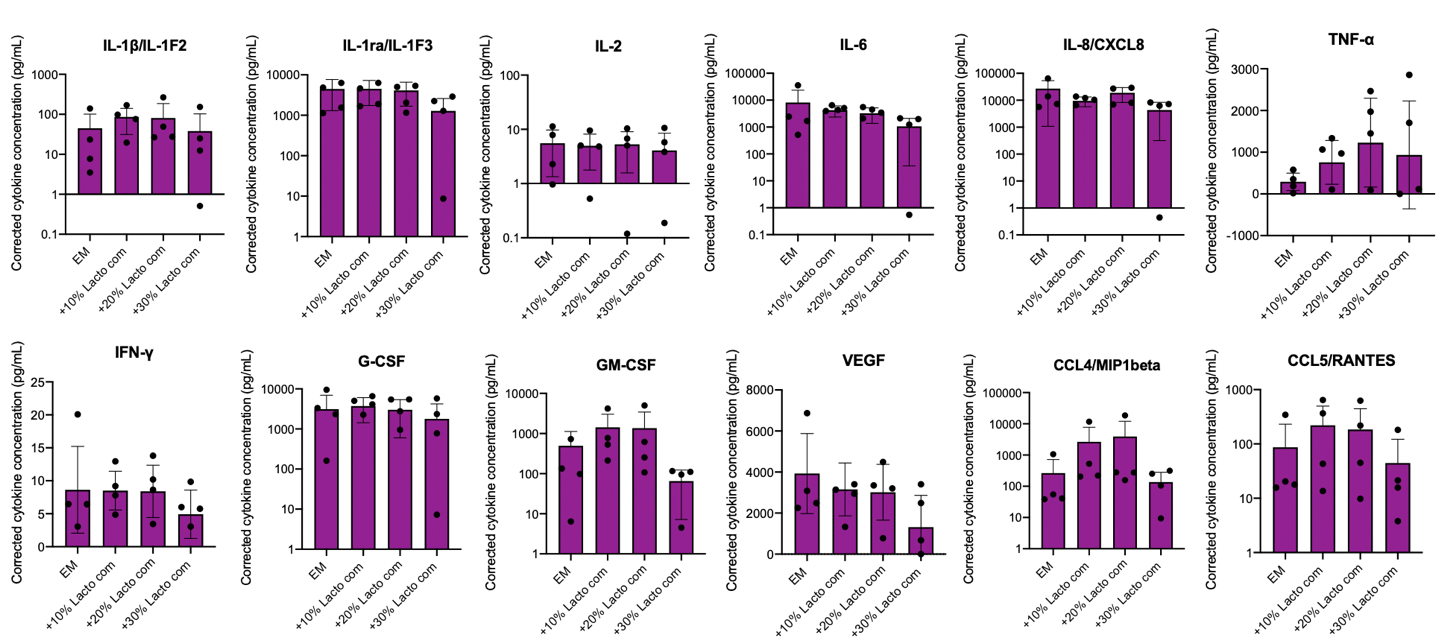
**

**A**

**B**

**C**

**Figure 9. A.** Benign organoid viability in response to LPS (1μg/mL, *E. coli* O111:B4) and *L. crispatus*- conditioned media for 24h or MRS broth for 48h. Organoid proliferation was significantly reduced in co-incubation of LPS and 30% LCC (p= 0.0097) but unaltered when LPS alone was used or combined with other LCC concentrations (10%, 20% v/v). MRS broth alone significantly decreased proliferation in the 30% v/v concentration (p= 0.027). **B.** Comparison of basal cytokine secretion by benign and endometrial cancer organoids after 48h of culture. **C.** Cytokine secretion by endometrial cancer organoids in response to increasing *L. crispatus*- conditioned media concentrations for 48h. Assay LoD: 1-10pg/ml. *RLU: Relative Light Unit;* *Lacto com: commercial L. crispatus; LPS: Lipopolysaccharide; MRS: MRS broth; Ben: Benign; Mal: Malignant.* * p-value < 0.05, ** p-value < 0.01; adjusted p values calculated by Dunn’s multiple comparisons test.
